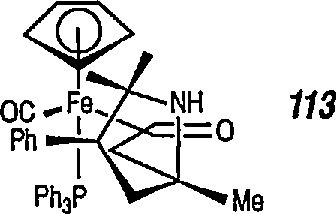# New Chemical and Stereochemical Applications of Organoiron Complexes

**DOI:** 10.6028/jres.096.002

**Published:** 1991

**Authors:** Alexander J. Fatiadi

**Affiliations:** National Institute of Standards and Technology, Gaithersburg, MD 20899

**Keywords:** applications, asymmetric synthesis, chemistry, chiral auxiliaries, enantiomers, iron-carbene complexes, organoiron complexes, overview, iron in biological systems, stereoselective reactions

## Abstract

The objective of this review is to provide a current overview of the rapidly developing chemistry of organometallic complexes and particularly organoiron complexes useful in asymmetric and stereoselective reactions. Also covered are stereoselective reactions of α, β-unsaturated acyl ligands bound to the chiral auxiliary [(η^5^-C_5_H_5_) Fe(CO)(PPh_3_)] and new applications of organoiron complexes in the synthesis of natural products. The mechanistic aspects and stabilizing effects of the Fe(CO)_3_ group for alkenes or conjugated dienes are discussed. A brief summary of recent work on the special role of iron in biological reactions is also included.

## 1. Introduction

Transition-metal organometallic chemistry has been one of the most active areas of chemical research for the past 35 years. A significant part of this research has been concerned with the use of transition-metal organometallics in organic synthesis [1].

Transition metals play an important role in both organic and inorganic chemistry. Transition-metal-mediated organic synthesis [1,2], catalysis [3–5], biomolecular synthesis [6], and organometallic conductors [7] are all current subjects of intense interest. Of particular note are the applications of organotransition-metal chemistry to the problems of constructing carbocyclic rings [8–9].

This review is primarily concerned with recent synthetic applications of organoiron complexes, with only brief comparison to their strong competitors namely, organochromium complexes. Metal carbonyl complexes are readily available, and serve as starting materials for the preparation of many other organometallic compounds. In the case of organoiron complexes, the synthesis begins with the use of such stable iron carbonyl reagents as pentacarbonyliron Fe(CO)_5_ (b.p. 103 °C/760 Torr), the dimer Fe_2_(CO)_9_ or the solid trimer Fe_3_(CO)_12_ [9] in their reactions with alkenes, dienes, or aromatic compounds. The nitrogen (or argon)-protected reaction is conducted either by heating an unsaturated substrate with the reagent, e.g., Fe(CO)_5_ without solvent, or using the high-boiling dibutyl ether, tetrahydrofuran or hydrocarbons as the solvent. The carbonyl reagents are regarded as highly toxic materials and all operations should be handled with care in a hood having an efficient exhaust. A vast number of tricarbonyliron complexes (comprising the Fe(CO)_3_ group) have been prepared and studied, and basic aspects of pentacarbonyliron reactions with a large number of organic functional groups have been reviewed [10–14].

Generally, iron carbonyls obey the 18-electron rule configuration in the formation of their organoiron complexes. For example, the mechanism by which Fe(CO)_5_ reacts with alkene is by initial dissociation of a molecule of CO, to give, at first, the 16-electron (coordinatively unsaturated) Fe(CO)_4_ complex, which then coordinates with the alkene to give an 18-electron complex, i.e., (alkene) Fe(CO)_4_; all these are π-bonded complexes with Fe(zero-valent). In such σ-bonded complexes as [(η^5^-C_5_H_5_)Fe(CO)_2_C_2_H_5_], the iron is probably best regarded as Fe(II) with a 6-electron (η^5^-C_5_H_5_)^−^ donor, a 2-electron ethyl (C_2_H_5_) anion donor, and 2-electron CO donors [14,15]. However, existence of a 19-electron intermediate [(η^5^-C_5_H_5_)Fe(CO)(PPh_3_) (Nu)CH_3_^+^] (where Nu is a pyridine nucleophile) has recently been reported [16].

The π-acid nature of carbonyl ligands makes anionic carbonyl complexes quite common, and the bonding capabilities of transition-metal clusters, including anions [Fe(CO)_4_]^2−^ or [Fe_2_(CO)_8_]^2−^, are reasonably well understood [17]. Substitution by tertiary phosphine (i.e., the PR3 groups) frequently converts unbridged polynuclear carbonyls into bridged structures, because bridging carbonyls are better π acids and are better able to handle the increased electron density. The physical and chemical properties of ironcarbonyl reagents, and their handling and use in synthesis of a variety of organoiron complexes (especially Fe(CO)_3_-substituted complexes), have been thoroughly reviewed and discussed [10–15], and the reader is referred to these comprehensive treatises.

In contrast to the electron-withdrawing effect of tricarbonylchromium, e.g., Cr(CO)_3_ in (arene) Cr(CO)_3_ complexes [18,19], tricarbonyliron, e.g., Fe(CO)_3_ in (diene)Fe(CO)_3_ complexes behaves as a net inductive electron donor [15–20]. Metal stabilization of intermediates [21,22] and fluxionality in polyeneiron complexes [23] have recently been discussed [24]. In regard to double bonds, a metal can activate, deactivate, or protect the double bonds for electrophilic or nucleophilic attack; also, it can resolve geometric and optical isomers, direct attack stereospecifically, and aromatize or dearomatize appropriate systems, and these achievements are often difficult or impossible to reach by standard organic-type reaction alone [14]. Some examples of metal action on double bonds that includes metal σ-bonds will be presented in this review.

Removal of the Fe(CO)_3_ group [14,20] is normally achieved by treating the complex with an oxidizing agent such as eerie ammonium nitrate (CH_3_OH, 0 °C) [25], ferric chloride, cupric chloride, or hydrogen peroxide usually between 0 °C and room temperature, in a solvent such as aqueous acetone or ethanol, or dilute acetic acid. The Fe(CO)_3_ group can also be removed by treatment with bromine or iodine in dichloromethane at low temperature (−40 to −78 °C), or treatment with trimethylamine *N*-oxide [(CH_3_)_3_N→O] in N,N-dimethylacetamide at 0°, or in acetone at room temperature [26]. Although trimethylamine *N*-oxide is a mild oxidant and extensively used for the cleavage of Fe(CO)_3_ groups without disturbing the stereochemistry, in certain cases the reagent is not specific, because it will also oxidize primary alcohols to aldehydes [27]. A recently introduced active manganese dioxide as a selective oxidant for removal of the Fe(CO)_3_ group and oxidative cyclization in one step could be an important reagent in the natural products synthesis [28]. The Fe(CO)_3_ protecting group may also influence the chemistry at sites remote from those of coordination.

## 2. Scope of the Review

Reactions of ironcarbonyl-coordinated alkenes and alkynes with tetracyanoethylene (TCNE) have recently been reviewed [29]; among the topics covered were reactions of main group metal complexes with TCNE, reactions of metallocenes with TCNE, and reactions of transition-metal complexes, including platinum-family complexes with TCNE. The objective of this review is to provide a current overview of the rapidly developing chemistry of organometallic complexes and particularly organoiron complexes useful in stereoselective synthesis. Several important topics in applications of organoiron complexes, to effect the regio- and stereospecific construction of organic molecules are covered and discussed in this review. The first involves diastereoselective reactions involving a complex in which a chiral auxiliary has been incorporated. Here the chiral auxiliary may either be the organic ligand of interest or other ligands on the metal (e.g., a chiral phosphine). The next topic involves the enantioselective addition of a nucleophile to an organoiron complex where the alkene, by having the metal complexed to one face of the π system, becomes the center of chirality. And in a new addition to this series, a chiral nucleophile can be added diastereoselectively to an achiral organometallic complex. Alternatively, the iron itself may be considered chiral by virtue of having different ligands attached. Thus, optically active metal-alkene and -alkenyl complexes, in which the coordinated ligand is the center of asymmetry, constitute reagents with significant potential in asymmetric synthesis, since nucleophilic addition to such ligands provides a means for creating one or more asymmetric saturated carbon centers, with high enantioselectivity. Also covered are new applications of iron carbenes in synthesis, applications of organometallics in the synthesis of natural products, and the Fe(CO)_3_− complexes from cyclic derivatives such as cyclopenta-, hexa- or heptadienes. Ferrocenes, bridged ferrocenes, iron porphyrins, capped iron porphyrins, iron pyrroles, and related organoiron compounds, and the unique role of iron in biological reactions will not be covered herein; however, a brief summary of work discussed in the recent literature on these subjects will be included. The application of acyclic butadiene iron-tricarbonyl complexes in organic synthesis has recently been reviewed at length by Grée [30]; also synthetic applications of enantioselective organo-transition metal mediated reactions have recently been surveyed by Blystone [31]. Because of space limitations, the literature cited and the topics discussed are highly selective. The literature from Chemical Abstracts covers approximately the period from 1970 through October 1990.

The credit for the development and application of organoiron complexes in organic and organometallic chemistry can be ascribed to brilliant contributions by Fischer [32,33], Pettit [34], Müller [35], Alper [10], King [36], Rosenblum [37–40], Brunner [41], Birch [14], Collman [42], Gompper [43], Sarel [44], Noyori [45,46], Pearson [20,21], Casey [47], Hegedus [13], Wojcicki [48], Reger [49], Helquist [50], Gladysz [51], Kerber [52], Wulff [53], Ojima [54], Negishi [55], Backvall [56], Herndon [57], von Gustorf [58], Grée [30], Seyferth [59], Davies [60–63], Liebeskind [64], Semmelhack [65], Brookhart [66], and others [31,67–73]. Credit is also due Fischer [24], Dötz [74], Jaouen [18], Semmelhack [19], Wulff [53,75] Hegedus [76], Yamashita [77], and others [77a,77b] for their studies and synthetic evaluations of the related organochromium complexes. A number of excellent, recent books and monographs covering general synthesis that are pertinent to this review of organometallic chemistry have been published [20,22,24,53,78–85].

New catalytic transition-metal-mediated carbon-carbon bond-forming reactions have found exceptional utility in organic synthesis [1,5,31,46,79,84–87] and numerous examples are illustrative of the recent synthetic usefulness of organoiron complexes [20,30,31,39,60,64,88–93].

## 3. Tricarbonyliron Fe(CO)_3_ Group Stabilization of Diene Systems

The incorporation of organometallic moieties into biologically important, unsaturated molecules is a field of increasing interest. Among many transition-metal carbonyls, the tricarbonyliron group [Fe(CO)_3_] has been found useful as a stabilizing group for alkenes, dienes, and related unsaturated systems, and the topic has been exhaustively studied and reviewed [14,20–22]. The Fe(CO)_3_ group attached to an organic ligand possesses a number of useful properties that may be exploited for synthetic transformations [14,20,21,65].

However, one of the important properties of the Fe(CO)_3_ group is protection of an alkenic (or dienyl) function during synthetic interconversions. The Fe(CO)_3_ group has been used to protect a B ring Δ^5,6^ diene during hydrogenation of a side chain Δ^22^ double bond in a steroid [94], and to protect a diene group during osmylation (OsO_4_-pyridine, 20 °C, 24 h) of a sesquiterpene intermediate [95]; the use of steroidal hormones labelled with metal carbonyls to assay receptor sites has also been described [96]. A recent use of the Fe(CO)_3_ group was in protection of stereochemistry of the (2*E*,4*E*)-dienoate unit of a polyene during homogeneous tritiation [97], regioselective reduction of allylic alcohols [98], or in stabilization of bicyclotetraenes [99]; also regio- and stereoselectivity of Bu_4_N[Fe(CO)_3_NO]-catalyzed allylic alkylation has been described [100]. Other examples illustrating the potential use of the Fe(CO)_3_ group as a diene protector during interconversion of the alkenic group have been reported [101–104]. Activation of alkane C-H bonds by organometallics (including organoiron complexes) has been reviewed [105,106] and bonding in metal-CO chemisorption, e.g., ironcarbonyls has been discussed [107]; also the electron affinity of (η^4^-1,3-butadiene) irontricarbonyl, e.g., η^4^–Bd·Fe(CO)_3_ has recently been determined [108].

### 3.1 Iron-Stabilized Cyclic Dienes in Regio- and Stereocontrolled Synthesis

Synthetic application of organometallic complexes is both an exciting and a challenging area of research: exciting because of the enormous areas of chemistry still awaiting exploration, and challenging because, to a large extent, the behavior of such complexes has still to be put on a firm, mechanistic basis [1,20,79,85]. Attachment of a transition-metal moiety (e.g., Fe(CO)_3_ group) to an alkenic ligand presents the organic chemist with unequalled opportunities to control the regio- and stereoselectivity of bond formation. The iron-carbonyl unit may direct the regio- and stereochemistry of nucleophilic addition, important in the synthesis of natural products.

**Scheme 1:**
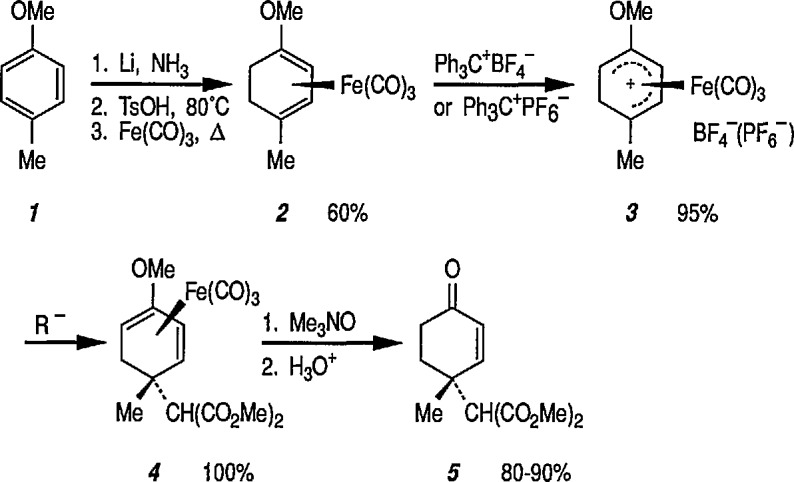


**Scheme 2:**
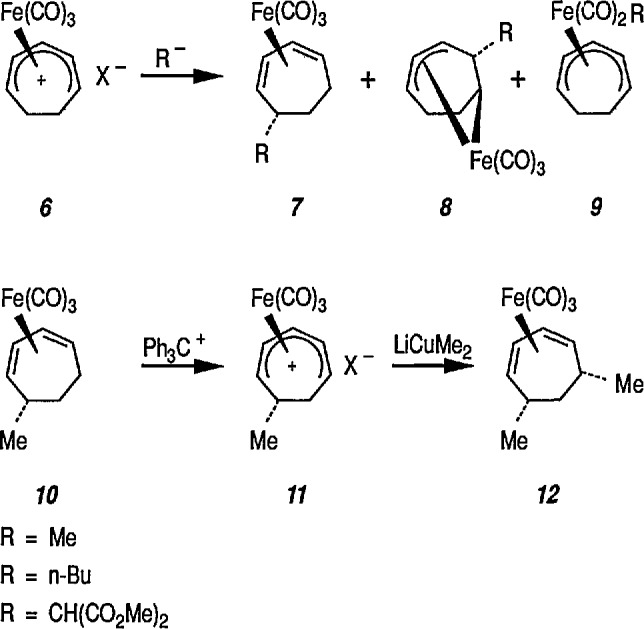


Reaction of 1,4-cycIohexanediene (readily available from the Birch reduction of benzene or its derivatives) [109] or 1,3-cyclohexanediene with pentacarbonyliron [Fe(CO)_5_] gives tricarbonyl (1,4- or 1,3-cyclo-hexanediene) iron complexes [14,20,109–112]. These complexes readily undergo hydride abstraction by treatment with triphenylmethyl (trityl) hexafluorophosphate $\left({\text{Ph}}_{3}C^{+}{\text{PF}}_{6}^{-}\right)$ or tetrafluoroborate $\left({\text{Ph}}_{3}C^{+}{\text{BF}}_{4}^{-}\right)$ in dichloromethane, to give tricarbonylcyclohexadienyliron hexafluorophosphate (or tetrafluoroborate) as stable, dull-yellow salts [32], e.g., metal-stabilized cyclohexadienyl cations [110–112]. These iron-stabilized carbocations are asymmetric due to the introduction of the Fe(CO)_3_ unit onto an achiral diene; they are highly reactive toward nucleophiles, leading to products that are also regiospecific and stereospecific, thus confirming the potential for asymmetric synthesis. In addition to its ability to stabilize dienyl cations, which led to useful, regiocontrolled nucleophile addition, the Fe(CO)_2_L group [L=CO, PPh_3_, or P(O Pha)] shows a potential stereochemical directing effect, and can be employed for synthesis of natural-product intermediates [113]. Pearson [89,114,115] made a thorough study of these complexes and, having established the rigid stereocontrol exercised by a tricarbonyliron group attached to either cyclohexanedienyl cations, conversion (1→ 5) [89] (Scheme 1), or to related cycloheptadienyliron complexes, conversions (6 → 9 and 10 → 12 [114,115] (Scheme 2) were achieved. The synthesis of cyclohexenone derivatives (e.g., 5) is summarized in Scheme 1, and cycloheptadiene derivatives (e.g., 7) (Scheme 2) show *cis* stereochemistry, as defined during the introduction of substituents at vicinal positions. Thus, the necessary stabilization and regio- and stere- ocontrolled functionalization of cyclohexadiene or cycloheptadiene systems using organoiron chemistry, can be accomplished. However, because of the fact that the cyclohexanedienyliron cation 3 possesses a plane of symmetry, the cation 3 and the nucleophile adduct 4 are in the racemic form. By the same reasoning, cyclohexanedienyl complexes of chromium (or molybdenum), or (arene)-Cr(CO)3 complexes [77a,77b] are (±) racemic mixtures. Same argument can also be applied to the cycloheptadienyl-iron derivatives (e.g., complexes 6–8 and 10–12). Generally, optical resolution of these complexes is required when used as intermediates for synthesis of natural products. Indeed, classical organoiron chemistry has acquired a new dimension, namely, stereochemistry.

Today, dienyl-Fe(CO)_3_ cations occupy a prominent place as emerging synthetic intermediates [14,110–112,116], largely due to their ready availability and low cost, and their reactivity toward nucleophiles that allows the preparation of substituted dienes which can be further transformed into a variety of natural products. To date, using the Pearson methodology [117,118] most of the synthetic applications have involved iron-stabilized cyclohexadienyl carbocations [89,117–120]. These applications show how the iron-carbonyl unit directs the regio- and stereochemistry of nucleophile addition. They also show how the iron-carbonyl unit can be used to stabilize otherwise inaccessible carbocations, thereby making them readily available as synthetic intermediates that can be further applied to a range of natural-product syntheses. Indeed, an application of (cycloheptadienyl)Fe(CO)L cations to the stereocontrolled construction of acyclic fragments of the macrolide antibodies, for example, mangnamycin B, has also been advanced [121] (see also sec. 7). Recently, Pearson et al. [122] reported an asymmetric induction as high as 90% *ee* during the reaction of enolates derived from optically pure sulfoximinyl esters with cyclohexadiene- or cycloheptadiene- Mo(CO)_2_Cp and cyclohexadienyl-Fe(CO)_3_ or cycloheptadienyl-Fe(CO)_2_P(OPh)_3_ complexes. Renewed interest [114,122] in methods for the preparation of homochiral organometallic π-complexes reflects new developments [123,124] employing their fully stereocontrolled alkylation reactions [125] in organic enantiomer synthesis. Recent work defined [126] strategic advantages available from the use of chiral organoiron complexes as intermediates in asymmetric synthesis. Recent extension to homochiral tricarbonyliron complexes in the 1,3-cyclohexadienyl series has been described. Also homochiral 6-methoxy substituted cyclohexadienyl series has been described. Thus homochiral 6-methoxy substituted cyclohexadienyltricarbonyliron complexes of high stereoisomeric purity have been prepared by complexation of the dimethyl ether of 1-methoxycyclohexa-1,3-diene-5,6-diol (available via microbial oxidation of toluene) with Fe_2_(CO)_9_ followed by demethoxylation with triphenylcarbenium tetrafluoroborate $\left({\text{Ph}}_{3}C^{+}{\text{BF}}_{4}^{-}\right)$ [127]. The attachment of substituents to six- and seven-membered rings, with transition-metal moiety (e.g., Fe(CO)_3_) as a stereodirecting template is a new technology for the construction of subunits of potential value in natural product synthesis or drug synthesis.

**Scheme 3:**
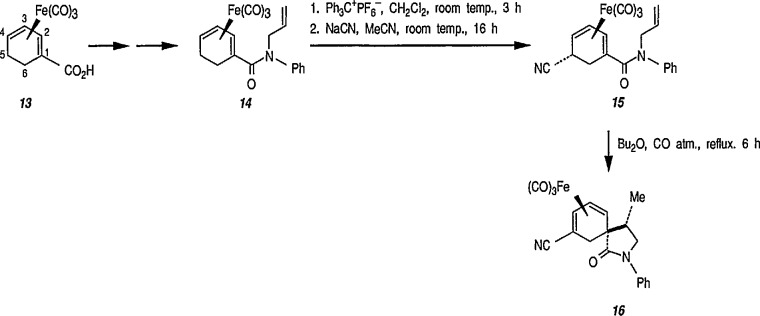


Cationic dienyl-Fe(CO)_3_ complexes are very reactive toward nucleophiles, and show considerable promise as synthetic intermediates [117]; however, there are some problems associated with their use. For example, traditional hydride abstraction by ${\text{Ph}}_{3}C^{+}{\text{PF}}_{6}^{-}$ (to yield a dienyl cation) fails, or is not regioselective in many cases [128,129], and a number of dienyl complexes do not undergo nucleophile addition with the most desirable regiochemistry [130]. Thermal and photochemical cyclization of diene-Fe(CO)_3_ complexes with electron-deficient alkenes (to yield spiro compounds) has been suggested as an alternative [131],

The stereocontrolled construction of quaternary [132] or spiro carbon [133,134] centers remains a challenging problem in organic synthesis. Recent extension [135] of the study [131] describes a unique, iron-mediated, intra-molecular ene-type reaction, leading to asymmetric, diastereospecific construction of quaternary carbon centers, with formation of spirolactones and spirolactams in enantiomerically pure form. Excellent stereocontrol during the ene-type coupling between diene- Fe(CO)_3_ groups and alkene in thermally induced spirocyclization can be achieved by appropriate substitution at C-5 of the diene ring; here, an electron-withdrawing cyano group yields a product without racemization, and an electron-donating phenyl group gives rearranged products, also optically active. For example, hydride abstraction from *N*-allyl-*N*-phenylamide derivative 14 (prepared from the enantiomerically pure acid 13) [136], followed by addition of cyanide, gave the optically pure nitrile 15, which yielded enantiomerically pure spirolactam 16 $\left({\left[\alpha \right]}_{D}^{25}+96.70,\;\text{acetone},\;1.5\right)$ in 87% yield under thermal conditions, e.g., thermal spirocyclization (Scheme 3). Similarly, optically pure spirolactones can be prepared [135]. This methodology permits application of asymmetric synthesis to a variety of spirocyclic, natural products and some new approaches to synthesis of natural products will be discussed later in the text.

## 4. Iron-Stabilized Carbenes in Organic Synthesis

**Scheme 4:**
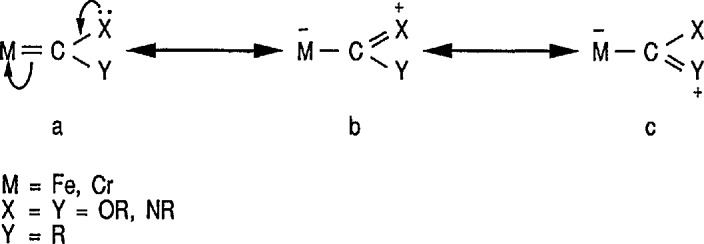


One of the most outstanding properties of transition metals is their ability to stabilize short-lived molecules as ligands in coordination compounds, and this is partially observed in metal-stabilized carbenes [137]. Ligands bound through a disubstituted carbon atom are known collectively as carbenes (e.g., in metal carbenes) even though they neither give rise to, nor are made from, free carbenes [138]. Several theoretical studies have been carried out in attempts to evaluate the strength of, as well as the barrier to, rotation about the expected metal-carbon double bond in transition-metal carbene complexes [139]. The structures of metal-carbene complexes may be understood in terms of various limiting forms that contribute to the stabilzation of the formally electron-deficient carbene carbon atom [74,137]. Three resonance structures, a |arwlr| b |arwlr| c, are shown in Scheme 4; structure a is stabilized by IT donation from the metal M (this structure makes the carbene nucleophllic); the most stabilization to the carbene comes from structures b and c, where substituents X and Y each serve as a π-donor. For heteroatom-substituted carbenes, there is thus a significant positive charge on the heteroatom X or Y (OR,NR), and substantial double-bond character between that heteroatom and the carbene atom.

The complexes that contain metal-stabilized carbenes are known for almost all transition elements; the development of the metal carbene chemistry is credited to the brilliant research of Fischer and his students [32,33,137]. The various transition-metal complexes can be divided into two groups on the basis of the chemical reactivity of the carbene atom [140–142]. The reactivity of carbene ligands is principally determined by the π-donor ability of the substituents on carbon. Carbene ligands with heteroatom substituents (e.g., O,N,C1), or other substituents capable of π interaction with the carbene atom, are called “electrophilic” or Fischer-type carbenes, for example, of chromium 17 [32,33,137,143–145] or iron 18 [146–147], and are usually subject to nucleophilic attack at the carbene carbon atom. Carbene ligands, without such substituents (for example, methylene or alkylidene ligands) require substantial π donation from the electron-rich metal and are called “nucleophilic” or Schrock-type carbenes [142, 148] and are usually subject to electrophilic attack at the carbene carbon atom. Thus, in the Schrock-type alkylidene complexes the metal-coordinated *sp*^2^-carbon atom is nucleophilic in character and displays an ylide-type reactivity. Electrophilic carbene ligands, including Fischer-type carbene complexes [32,143], may be viewed as singlet carbenes donating a pair of electrons via an *sp^2^* hybrid orbital, while receiving back-donation from the metal into an empty/j orbital [144,145].

However, the reactivity of any given carbene ligand varies considerably from complex to complex; for example, methylene ligands are usually nucleophilic, but can become electrophilic for complexes bearing a positive charge. Thus, the discovery of heteroatom-stabilized carbene complexes (L_n_M = CR(OR)) by Fischer [33] and of heteroatom-free metal alkylidene complexes (L_n_M = CR_2_) by Schrock [142, 148] laid the foundation for the recognition of reactive carbene complexes as decisive intermediates by many metal-catalyzed transformations of organic substrates [74,78,87,137].

**Figure:**
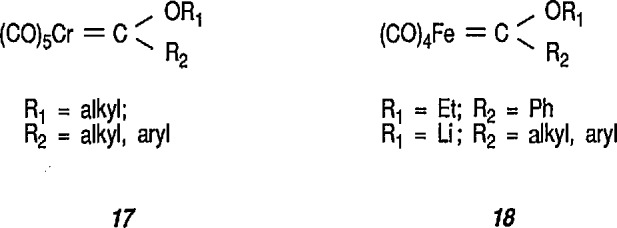


Although carbene complexes have been studied intensively for more than 2 decades, it is only very recently that useful applications of these complexes to organic synthesis have been developed [137,139,149,150–154].

Transition-metal carbene complexes are generally recognized as important, reactive intermediates in organometallic chemistry, in particular in certain catalytic reactions, e.g., alkene metathesis [31,155], alkene polymerization [156], the Fischer-Tropsch synthesis [157,158], and important cyclopropanation reactions [159]. Carbenes and carbene precursors are known to react with transition-metal complexes to produce alkyl [160] and alkylidene [161] complexes, as well as a host of compounds in which the intact precursor molecule is coordinated to the metal center [162, 163].

As synthetically useful reagents, carbene complexes are not only suitable as carbene-transfer reagents but also undergo interesting cycloaddition with other ligands in the CO-ligand sphere. Their treatment requires techniques no more complicated than those used for Grignard reactions. However, recently developed, stabilized carbene complexes are handleable in air, can endure temperatures of 100 °C or more, are stable in mild aqueous acids and bases, and are soluble in organic solvents to the point where most can be rapidly eluted from silica gel columns with hexane [150–152]. Thus, carbene complexes can also be used in the synthesis of natural products; a few recent examples include synthesis of naturally occurring furochromones [153], (β-lactams (penicillin analogs) [164], and antibiotics [151,165,166].

In comparison to the wide application of chromium carbene complexes in organic synthesis [18,19,74,137,151,165–170], the Fischer-type carbene complexes of iron (e.g., 18) have been but little explored [147]; indeed, many of the iron carbene complexes are of proven synthetic utility [66a,^171^–^175^].

### 4.1 Preparation and Some Reactions of Iron-Carbene Complexes

Synthesis of neutral and cationic metal-carbene complexes has been discussed [176]. Neutral iron-carbene complexes of type 21 are readily available by the method of Fischer [137,143] by treatment of iron pentacarbonyl 19 with lithium or a Grignard reagent, followed by alkylation of acyl complexes on oxygen [147,177] (conversion 19 → 20 → 21). Carbene complexes of type 21 have also been prepared by photochemical exchange of a CO ligand in Fe(CO)_5_ with alkylidene [178].

**Figure:**
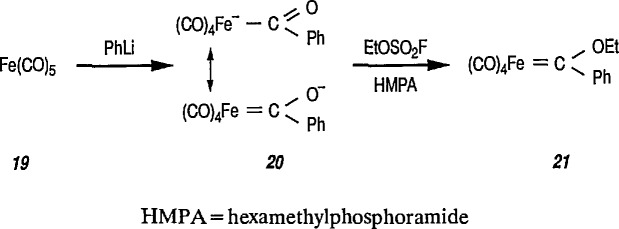


A few additional routes to 0-substituted, Fischer-type carbene complexes are described next. For example, methoxycarbene complexes 24 are readily prepared by treatment of the bromide (22) with the appropriate lithium acetylide, to give (23), followed by protonation of the corresponding vinylidene complexes, and subsequent addition of methanol [179–181]. Alternatively, 24 may be obtained from the corresponding acyl complexes 25 [61,64] following treatment with trimethyloxonium tetrafluoroborate [182].

**Figure:**
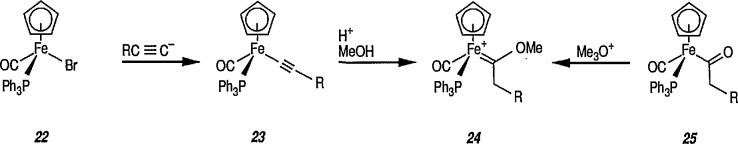


Cationic carbene complexes can also be prepared by the alkylation of neutral acyl complexes [183]. There are two efficient, general syntheses of electrophilic (cationic) metal-carbene complexes: the addition of (1) an electrophile to a M-CHR-X (X = OR, SR, Cl) derivative [184], and (2) acids to vinylmetal complexes [184–186]. For example, synthesis of important cyclopropanic reagent, e.g., the cationic iron-carbene complex 24 is based on the addition of an electrophile to FeCH = CH–C(CH_3_)_2_X system [185]. Thus, addition of CH_3_Li to the ketone group of the acyliron complexes 26 produced tertiary alcohol 27 (62% yield). Addition of HBF_4_ in diethyl ether to an ether solution of 27 at −23 °C gave the iron-carbene complex 28 as a red-orange solid.

**Figure:**
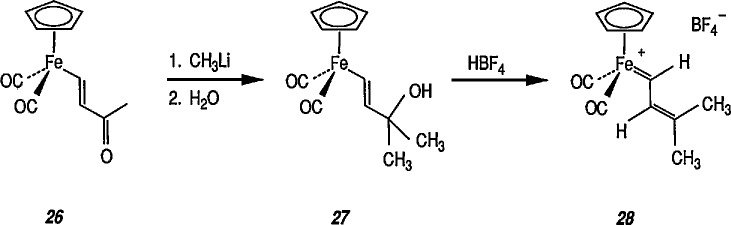


### 4.2 Reactions of Electrophilic Iron-Carbene Complexes

**Scheme 5:**
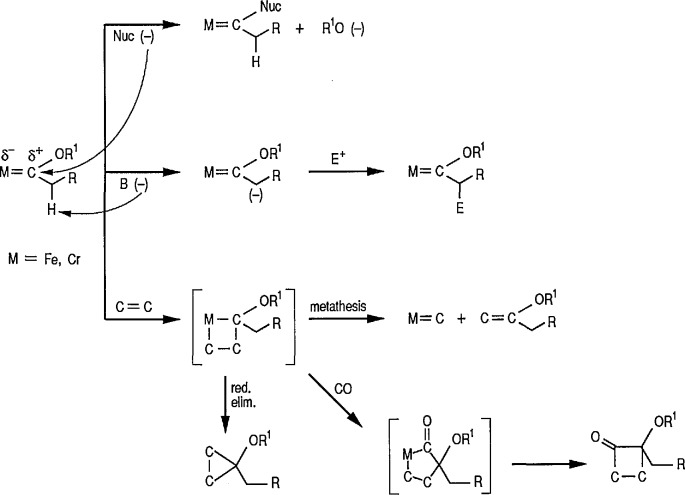


Electrophilic or Fischer-type iron-carbenes are usually subject to a two-fold nucleophilic attack, resulting in either exchange of the heteroatom substituents on the carbene carbon, or ligand substitution at the metal. The general patterns of reactivity of electrophilic carbene complexes are summarized in Scheme 5.

Generally, interaction of carbene complexes with alkynes, metathesis, and cyclopropanation reactions are important reactions of metal carbenes. However, it is the carbene-transfer reactions, and particularly, cyclopropanation reactions, which probably proceed through metallic intermediates, that are of the most importance in organic synthesis.

### 4.3 Additional Reactions of Iron-Carbene Complexes

**Scheme 6:**
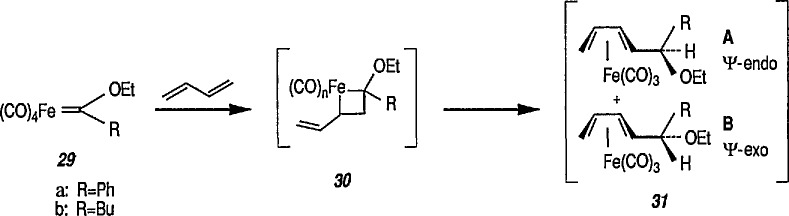


New coupling reactions of conjugated 1,3-dienes with iron-carbene complexes to give (1,3-diene) Fe(CO)_3_ derivatives have recently been reported by the Semmelhack group [187]. Thus, coupling of the iron-carbene 29 (R=Ph) with 1,3-butadiene gave via the ferracyclobutene intermediate 30, a new way for the preparation of (η^4^-1,3-diene)-Fe(CO)_3_ complexes, e.g, 31A + 31B, bearing an allylic ethoxyl group (Scheme 6). Complexes of 1,3-dienes with Fe(CO)_3_ provide starting points for useful synthesis methodology via direct nucleophile addition [188], and by conversion into (η^5^-pentadienyl) Fe(CO)_3_ cationic complexes which are powerful electrophiles [189,190].

**Scheme 7:**
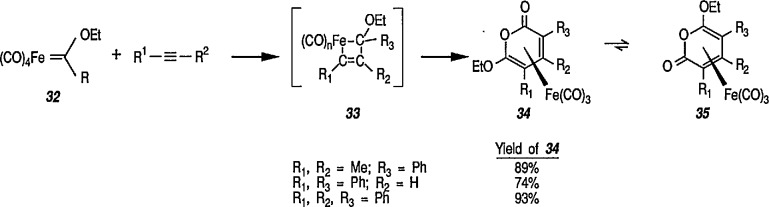


**Scheme 8:**
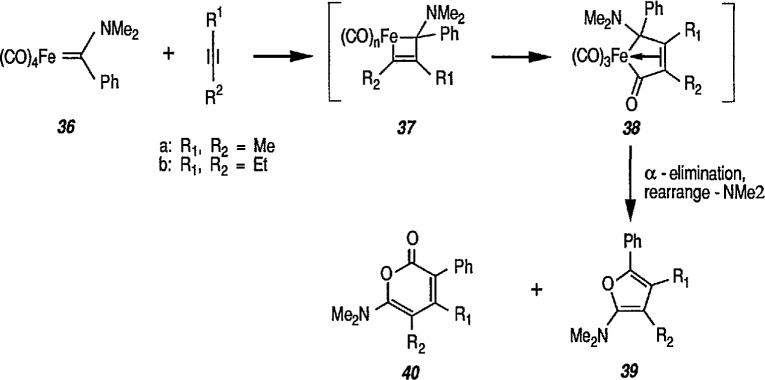


Another synthetically interesting new reaction of iron-carbene complexes with alkynes has been reported by the same group [17,191]. It has been demonstrated [17] that iron-carbene complexes, e.g., 32 react with alkynes to form, also via the ferracyclobutene intermediate (33), pyrone complexes 34↔ 35 (Scheme 7). Similar reaction of (aminocarbene)-iron complexes with alkynes [191] can lead to relatively little studied 5-aminofurans (the major product) and 6-amino-α-pyrones (the minor product), e.g., conversion 36 → 37 → 38 → 39 → 40 (Scheme 8). This is a remarkable reaction, with a strong potential for future synthesis; however, the mechanism by which it occurs is not yet known, although a somewhat complex proposal can explain the products formed [17,191].

**Scheme 9:**
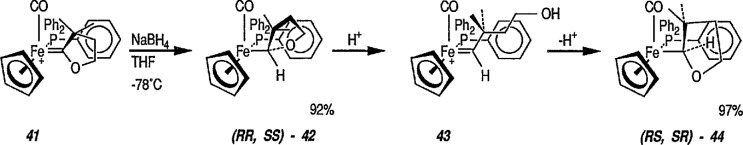


Recently, Ayscough and Davies reported [192] a stereoselective hydride reduction of a cationic iron-carbene complex [(η^5^-C_5_H_5_)Fe(CO)(PPh_3_) = COCH_2_CH_2_C^−^Me_2_]^+^ (41). This study demonstrates the remarkable stereocontrol exerted by the chiral auxiliary (η^5^-C_5_H_5_)Fe(CO)(PPh_3_) in reactions on the α-carbon atom, where reduction of (41) proceeds completely stereoselectively to give the kinetic product (42), which then epimerizes (43), also completely stereoselectively, to the thermodynamic product (44) (Scheme 9). These results are readily explicable in terms of a simple conformational model, and the phenomenon can be expected to be general for all cases where there is a very bulky Cα substituent (e.g., at carbon centers directly attached to the chiral auxiliary) [192].

### 4.4 New Iron-Nitrene Complexes for Synthesis

The chemistry of metal-carbene complexes (including those of iron and chromium) has been extensively investigated, and many such complexes have found impressive synthetic utility [137,150–153,193–195]. In contrast, the chemistry of nitrene ligands to iron or chromium, e.g., [(CO)_5_M = NPh (M = Fe, Cr) (metal-imides)], remains relatively unexplored [196], partially because of their instability [197]. Like that of many reactive organic ligands, stabilization of nitrenes by organometallics can be achieved through coordination of the ligand to adjacent metal in cluster compounds, with the μ_3_-NR coordination mode being the most common [140,141,193–195].

Recently, formation of imidates, amides, amines, carbamates, and ureas from the (μ_3_-NPh ligands of the complex Fe_3_(η_3_-NPh)_2_(CO)_9_ has been reported [198]. The bis(nitrene) cluster Fe_3_(η_3_-NPh)_2_(CO)_9_ reacts with Li(HBEt_3_), MeLi, PhLi, or NaOMe, to form formyl and acyl clusters of type [Fe_3_(η_3_-NPh)_2_(CO)_8_C(O)R]^−^ (R=H; Ph; Me; OMe). On further treatment with EtOTf (EtOSO_2_CF_3_), the benzoyl cluster (R = Ph) thus obtained yields the nitrene-carbene cluster [Fe_3_(η_3_-NPh)_2_(CO)_8_ C(OEt)Ph], and this mixed cluster is used for the preparation of such useful derivatives as imidates, amides, or amines by utilizing the η^3^-NPh ligands of Fe_3_(η_3_-NPh)(CO)_9_. This is a new synthesis adventure, and no doubt more studies in this direction will be forthcoming.

## 5. Iron-Catalyzed Synthesis and Some Reactions of Cyclopropanes. Cyclopropanation Reaction

The limits of stability of strained hydrocarbons, including cyclopropanes, have been substantially clarified over the course of the past 30 years [199,200]. The chemistry of the cyclopropyl group has recently been discussed at length [201]. The torsional strain in the three-membered ring imparts a high degree of reactivity, which can lead to the fragmentation of cyclopropanes; this process is apparently regio- and stereo-chemically controlled [202]. Because of the various cleavage reactions and rearrangements in which they participate, cyclopropanes frequently serve as valuable synthetic intermediates leading to other ring systems, or to acyclic products. The cyclopropane ring system is also seen as an important structural feature among many classes of naturally occurring compounds [66]. Fused, and bridged, polycyclic systems containing a cyclopropane ring have been shown to be useful intermediates in organic synthesis [203,204]. New synthetic approaches to carbocycles via intramolecular cyclopropanation reaction have recently been discussed [202], and a recent summary on cyclopropanes as building blocks for organic synthesis has appeared [205]. The reaction of Fischer-type transition-metal carbene complexes with alkenes are known to occur under the proper conditions to give cyclopropane products in a formal [2 + 1] cycloaddition [66]. [4 + 2] cycloaddition of Fischer carbene complexes with 1,3-dienes to give cyclopropanes and involving a zwitterionic intermediate has recently been observed [206]. However, the reaction of Fischer carbene complexes with enyne substrates yields bi- and tricyclic cyclopropane-containing carbon skeletons [207]. The paper also discusses various modes of metal carbene-alkene and metal carbene-alkyne couplings, including intra- and intermolecular cyclopropanation reactions.

### 5.1 Synthesis of Cyclopropanes via Carbene-Transfer Reaction

A cyclopropanation reaction involving transition-metal carbene complexes has recently been reviewed [66,208]. Among the large number of methods developed for the synthesis of cyclopropanes, the majority of them may be placed in two broad categories: (1) addition of carbenes, carbenoids, or related species to alkenes (to yield intermediates for carbene-transfer reactions) and (2) intramolecular coupling, or alkylation reaction [209,210].

**Scheme 10:**
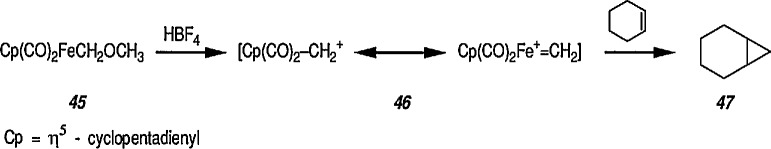


Electrophilic, cationic complexes of iron are efficient cyclopropanating agents, particularly for unfunctionalized alkenes. These complexes are unstable, and are usually generated and used *in situ.* As observed earlier by Pettit et al. [211], and others [212], when methoxymethyliron complex 45 was treated with acid, it underwent cleavage to a short-lived, cationic methylidene complex 46, as evidenced by the formation of norcarane 47 when the cleavage was performed in the presence of cyclohexene (Scheme 10). Several methods have been developed for the preparation of more-stable metal carbenes suitable for cyclopropanation [213]; some of these require replacement of the simple methylidene group by an ethylidene [172], isopropylidene [159,214], allyhdene [185,215], or benzylidene [216] group.

**Scheme 11:**
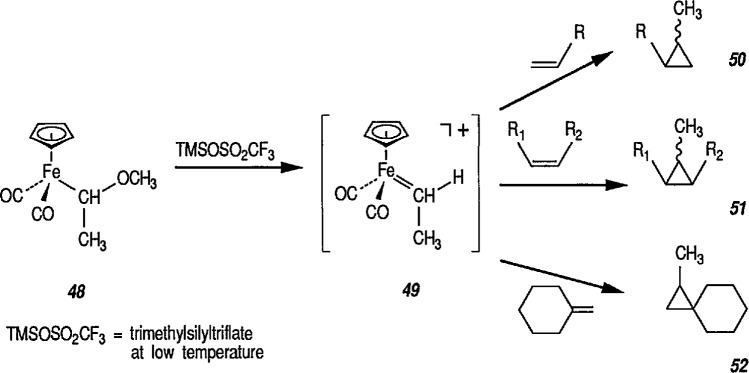


Brookhart has used methoxyalkyliron complexes (Scheme 11) to generate relatively stable ethylidene [217,218], benzylidene [66a,^174^,^219^], and cyclopropylidene [220] transfer reagents. Note the generation of the required cationic iron complex 49, following treatment of the methoxyalkyliron complex 48 with trimethylsilyl triflate at low temperature, and then with alkenes, to give the corresponding cyclopropanes 50, 51 and 52. The most synthetically efficient and best studied cyclopropanating reagents [141] are the cationic carbene complexes of the general structure [(η^5^-C_5_H_5_)(CO)_2_Fe-CRR^1^]* (R = R^1^ = H) [147]; R = H, R^1^ = aryl [174]; R = H,R^1^ = CH_3_[166,172,175], R = R^1^ = CH_3_ [184], and their various phosphine derivatives [147,159,221].

**Scheme 12:**
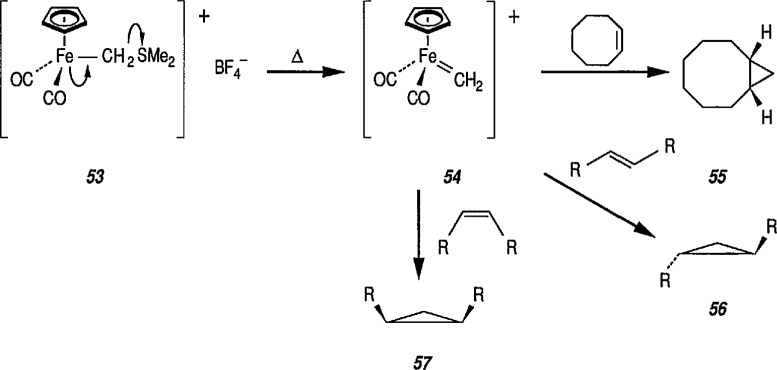


Recently, more-stable metal carbenes have been synthesized, and applied as cyclopropanation reagents in synthetic organic chemistry. The new procedure employs sulfonium derivatives of the structure [(η^5^-C_5_H_5_)FeCH_2_SR_2_]^+^ (metal-bonded sulfonium ylides) in which a neutral dialkyl sulfide serves as the leaving group. Thus, Helquist et al. [171,172,209] found that treatment of the stable (dimethylsulfonium) methyliron complex 53 with alkenes in refluxing 1,4-dioxane produced, via the carbene intermediate 54, and methylidene group transfer, cyclopropanes 55, 56 and 57 in excellent yields (Scheme 12) (table 1). The reaction is stereospecific, giving *cis*-cyclopropanes with *cis*-alkenes, and *trans*-cyclopropanes with *trans*-alkenes. The ethylidene group is similarly transferred when the related (methylphenylsulfonium) ethyliron complex is used; in this case also, the stereochemistry of the alkene is maintained [172].

The precise mechanism of the cyclopropanation reaction is yet to be determined. It has been clearly established that, in the transfer reaction, the electrophilic carbene complex attacks the nucleophilic alkene with substantial charge development in the transition state. A transition state involving unsymmetrical attack, with partial charge build-up on only C-2 and not a more-symmetrical model where equal charge builds up to C-1 and C-2 of the alkene, has been advanced [66]. The future challenging chemistry of, for example, metallo-bis (methylene)phosphoranes, e.g., (η^5^-C_5_H_5_)Fe(CO)_2_{P[ = C(SiMe_3_)_2_]_2_} [222] or other metal-carbene-type complexes, comprising such highly reactive structures as Fe = N,S,P or Fe = Si,Ga,B,As, etc., still await exploration. Recently, reactions of iron ω-haloalkyls with silver (I) to form cyclopropanes have been described, e.g., (η^5^-C_5_H_5_)(CO)_2_Fe(CH_2_)_3_Br + Ag BF_4_ → cyclopropane (73% yield). The mechanism of this reaction and its relevance to the cyclopropanation reactions of cationic metal carbenes and alkenes has been discussed [223].

General Procedure for Cyclopropanation of Alkenes with the Sulfonium Salts (53), 1,1-Diphenylcyclopro-nane [209]:

The required ${\text{CpFeCH}}_{2}S^{+}{\left({\text{CH}}_{3}\right)}_{2}{\text{BF}}_{4}^{-}$ (53), Cp = (η-C_5_H_5_) was readily prepared by the reaction of the ferrate Na^+^[(η^5^-C_5_H_5_)Fe]^−^ with chloromethyl methyl sulfide to give the alkylation product, which was then treated with a methylating agent as shown.

$$\begin{array}{c} {\text{Na}}^{+}\left[\text{Cp}{\left(\text{CO}\right)}_{2}\text{Fe}\right]\overset{{\text{ClCH}}_{2}{\text{SCH}}_{3}}{\to }\left[\text{Cp}{\left(\text{CO}\right)}_{2}{\text{FeCH}}_{2}{\text{SCH}}_{3}\right]\overset{{\left({\text{CH}}_{3}O\right)}_{2}{\text{CH}}^{+}{\text{BF}}_{4}^{-}}{\to }\\ \left[\text{Cp}{\left(\text{CO}\right)}_{2}{\text{FeCH}}_{2}S^{+}{\left({\text{CH}}_{3}\right)}_{2}\right]{\text{BF}}_{4}^{-}\left(53\right) \end{array}$$

The unrecrystallized 53 (35g, 100 mmol) as a yellow powder was placed in a 200-mL, round-bottomed flask equipped with a magnetic stirring bar. 1,1-diphenylethylene (9.3 g, 9.1 mL, 52 mmol) and 1,4-dioxane (25 mL) were added, the flask was equipped with a reflux condenser, and the mixture was stirred while it was boiled at reflux for 12–14 h under a nitrogen atmosphere. After the brown mixture had cooled some-what, hexane (75 mL) was added, and the mixture was stirred in the air as it cooled to 25 °C, filtered, and the retained solid washed with additional hexane. The combined filtrates were concentrated by rotary evaporation, and the crude product was purified by flash chromatography (silica gel, hexane). The colorless oil was distilled through a short-path apparatus to give 8.76 g (88%) of 1,1-diphenylcyclopropane as a clear, colorless liquid bp 89 °C (0.8 Torr).

### 5.2 Stereoselective Synthesis of Cyclopropanes

**Scheme 13:**
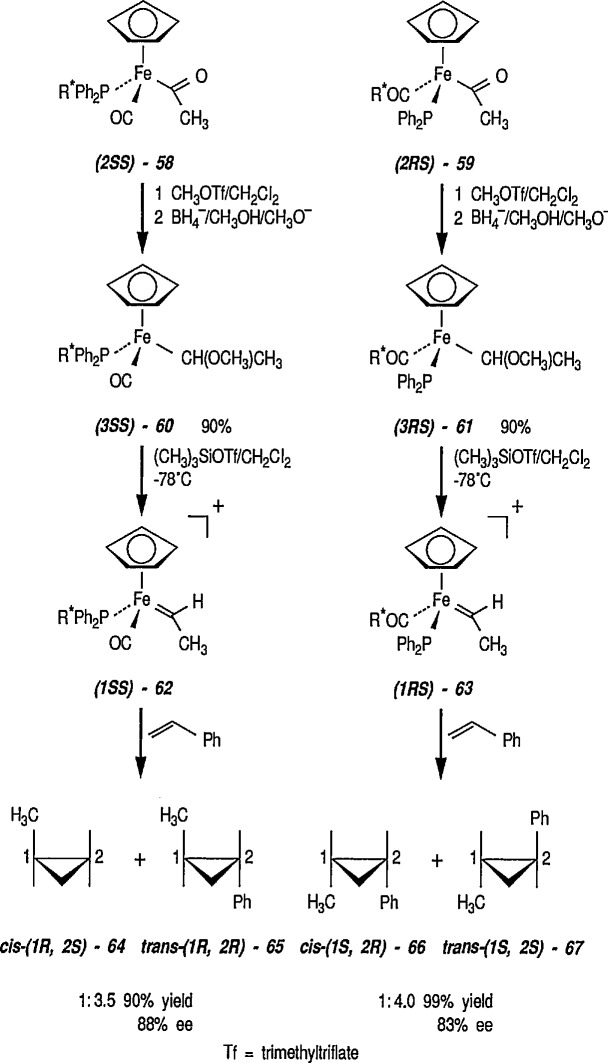


Transfer of the carbene ligand from optically active transition-metal-carbene complexes to alkenes represents a potentially useful and general method for the enantioselective synthesis of cyclopropanes [224], and this task was successfully accomplished by the Brookhart group [225]. The starting complexes were obtained as a pair of iron acyl diastereoisomers, e.g., 58 (2SS) and 59 (2RS), differing in configuration at iron. These were converted into the desired cationic iron-carbene complexes 62 and 63 via intermediates 60 and 61, as shown in Scheme 13. The efficient transfer of the ethylidene group from these two diastereoisomeric ethylidene complexes, e.g., [(S_Fe_S_c_) – (η^5^-C_5_H_5_)(CO) (PPh_2_R*)Fe = CHCH_3_]^+^ (62) (ISS) and (63) (IRS) [R* = (S)-2-methylbutyl)], differing only in the configuration at iron, to styrene, gave respectively *cis*- and *trans*-1-methyl-2-phenylcyclopropanes 64 and 65, and 66 and 67, with the high enantiomeric excesses shown. Since two diastereoisomers, e.g., 62 and 63 gave cyclopropanes having the opposite configuration in practically the same enantiomeric excesses, optical induction must be due to chirality at the metal. A study [225] discussed at length the role of the metal (e.g., iron) vs. ligand chirality in the optical induction. With iron-ethylidene complexes having a chiral phosphine in place of one CO ligand, high asymmetry induction was expected (and observed) [224].

A recent related study by the same group [226] discussed kinetic and thermodynamic diastereoselectivities of precursors to optically pure chiral-at-iron carbene complexes of the type (η^5^-C_5_H_5_)(CO)(PR_3_) Fe* = CHR^+^ (R = Ph, Et); the study reports photosubstitution reactions, and hydride or methoxide addition to a series of carbene complexes. These complexes transfer the carbene moiety to alkenes to give cyclopropanes often with high enantioselectivity [225].

Additional syntheses of optically active cyclopropanes by the use of chiral butadiene-tricarbonyliron complexes have been reported [227–229]; particularly, preparation in this way of formylcyclopropanes of high enantiomeric purity provided the key intermediate for the synthesis of low toxicity insecticide pyrethroids [228,229].

### 5.3 Metal-Induced Rearrangements of Cyclopropyl Alkenes

A brief overview of reactions of cyclopropane derivatives in the presence of iron pentacarbonyl, Fe(CO)_5_, or its dimer Fe_2_(CO)_9_, is included for continuity. This exciting chemistry has been studied by Sarel, and many metal-induced rearrangements of, and insertions into, cyclopropyl alkenes have been reviewed by that author [44].

The polarizability of the cyclopropane σ bonds, resulting in a tendency to undergo electrophilic attack by coordinatively unsaturated transition-metal complexes has been described [230,231]. The cyclopropane bonds are known to be weaker than normal σ bonds, and they are consequently susceptible to attack by reagents that attack double bonds.

In analogy to dienes, vinylcyclopropanes (VCP) can be induced to form vinylcyclopropane-iron π complexes by such zerovalent transition-metals as Fe(CO)_5_. Studies [44] led to discovery of at least five distinctly different modes of metal-mediated reactions of vinylcyclopropanes, depending on the substrate and the reaction parameters. These modes are as follows.

**Scheme 14:**
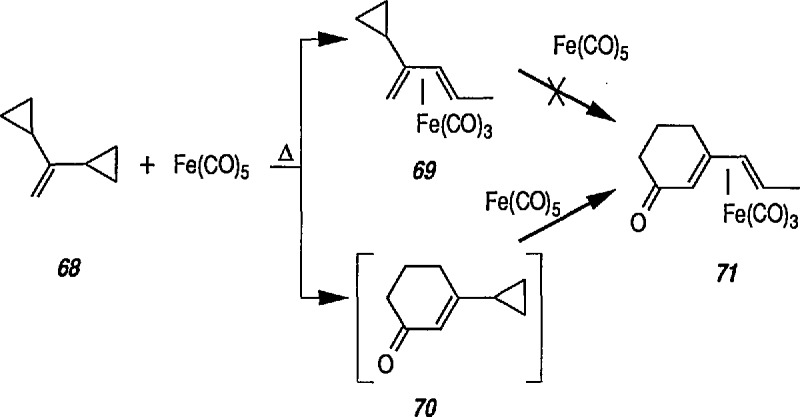


1. Heat-induced rearrangement of vinylcyclopropanes to diene π complexes. For example, the Fe(CO)_5_-mediated thermolysis of 1,1-dicyclopropylethylene (68) in boiling dibutyl ether led to the unexpected product 71 (via possible intermediate 70), together with the respective 1,3-diene tricarbonyliron π complex 69 (an example of vinylcyclopropane-diene rearrangement) [232,233] (Scheme 14).
2. Photoinduced carbonyl insertions across the VCP system, to afford cyclohexenones, conversion 72 → 73 + 74 + 75 [232,234].

**Figure:**
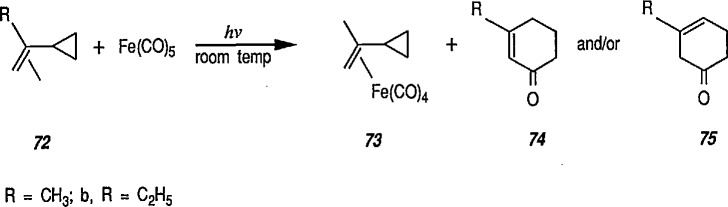
3. Metal insertion into the VCP system to give σ,π-allyl complex, conversion 76 → 11 [235].

**Figure:**
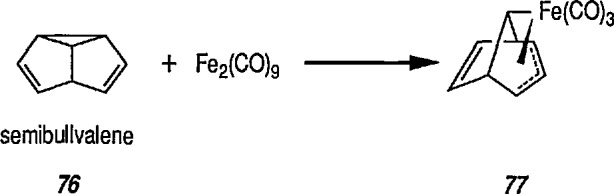
4. Photoinduced acyl-metal insertions into the vinylcyclopropane system. For example, photoreaction of α-thujene 78 with Fe(CO)_5_ occurs in both a stereospecific and a regiospecific manner, conversion 78 → 79 [44]. More examples of acyl-metal insertions into vinylcyclopropane systems have been reported [236].

**Figure:**
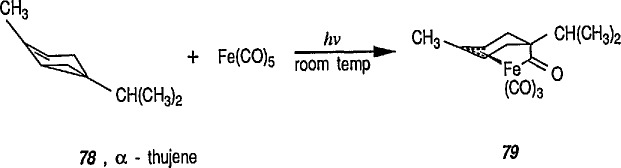
5. Dicyclopropylacetylene as a multi-π-electron ligand. Adding extra unsaturation to the original C-C π-linkage was shown [237,238] to render the cyclopropyl group inert to attack by iron carbonyl. For example, photoreaction of cyclopropylacetylene 80 with Fe(CO)_5_ gave rise to benzoquinones 81 and 82 originating from the insertion of carbonyls between the molecules of acetylene while leaving the cyclopropane ring itself intact [237].

**Figure:**
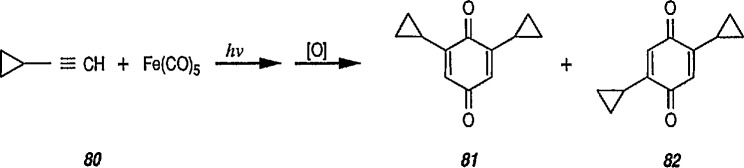

Thus, iron pentacarbonyl emerged as an efficient “homodienophile” having remarkable stereospecific and regiospecific characteristics. Vinylcyclopropanes and divinylcyclopropanes, on the other hand, emerge as novel sources of four and six π electrons suitable for metal coordination. The special features characterizing the interaction between zerovalent transition metals (e.g., Fe(CO)_5_ or Fe_2_(CO)_9_) and multi-σ, π-electron systems open up a new vista of chemical research of great synthetic interest [44].

Recently, Goldschmidt and Crammer [239] discussed vinylcyclopropane photorearrangements in the presence of Fe(CO)_5_ and extended the original Sard’s studies [232,233,240]. The reaction mechanism was elucidated by low-temperature (−50 °C) irradiation of vinylcylopropane and Fe(CO)_5_ giving two unstable Fe(CO)_4_-coordinated isomers [241].

### 5.4 A New Class of σ-Cyclopropenyliron Complexes

Whereas π-complexes of cyclopropenyl ligands with symmetrical as well as unsymmetrical coordination [2,242] are today an extensive class of compounds, σ-cyclopropenyl complexes have thus far not been generally accessible [243]. An earlier report described the synthesis of σ-cyclopropenyl derivatives of μ^5^-cyclopentadiene dicarbonyliron 83 in which the metal is bonded to the methylene C atom of the cyclopropane. Such α-cyclopropenyl metal complexes as 83 can be viewed as derivatives of antiaromatic cyclopropenide ions. Recently, Gompper and Bartmann [43] extended this series, and reported the preparation of complexes, e.g., 85, in which the metal is bonded to the double bond of the three-membered ring. The complexes of type 85 are simply prepared by reaction of the reported [244,245] salt 84 with nucleophiles (Nu^−^); these complexes are appreciably more stable than compounds of type 83.

A recent report [246] on a related work described the reaction of l,2,3-triphenyl-3-trifluorovinyl-cyclopropene with [Fe_2_(CO)_9_] to give a Fe(CO)_4_-complex containing a coordinated η^2^-vinylcyclopropene; on subsequent irradiation a novel ring expansion occurred to give an air stable Fe(CO)_3_-η^4^-cyclobutadiene complex.

**Figure:**
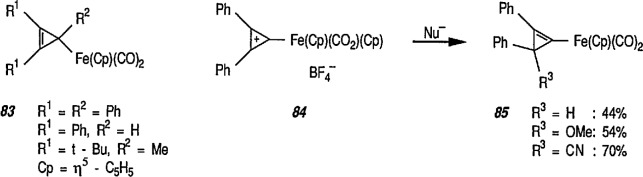


## 6. Stereochemical Control of Organic Reactions by Use of Chiral Organoiron Reagents

Asymmetric synthesis is a widely pursued goal in modern synthetic chemistry [247–250]. A number of systems that feature C_2_ symmetry elements in their ligands, such as the Sharpless epoxidation reagent [249,251,252], the Noyori catalyst [253,254], Masamune’s boranes [255], Davies’ chiral acyliron auxiliary [60,63], or Liebeskind’s chiral iron complexes [64] have proved so successful that much confidence is now placed in the use of such symmetry elements in the design of reagents and catalysts [256]. Moreover, enantioselective catalysis with transition-metal complexes [31,257], enantioselective catalysis with enzymes [258–260], inorganic “enzymes” [261], chiral ligands for asymmetric synthesis [251], and asymmetric natural products as sources of chiral synthons [262–264] are now well established chiral synthetic methods [265–267]. Also, the importance of the spatial arrangement of molecular groups for asymmetric recognition has recently been emphasized [268]. Chiral synthesis is currently among the most exciting areas of organic chemistry [269–285]. Many syntheses, and very often the most elegant, have made use of the natural “chiral pool.”

Organotransition-metal complexes form a class of very important compounds, not only because of their inherently interesting physical and chemical properties but also because of the significant impact they are having on synthetic organic chemistry [79,286–288]. Many novel synthetic applications of organotransition-metal complexes have been reported in the recent literature, which provides methods for synthetic transformations difficult or impossible to achieve by more conventional routes. Among those, such organoiron complexes as [(η^5^-C_5_H_5_)Fe(CO)(PPh_3_)] [79,286,289] or [η^5^-C_5_H_5_)Fe (CO)_2_] [209,290–292] have been studied extensively as effective reagents for selective organic transformations. Triphenylphosphine-substituted analogues of the [(η^5^-C_5_H_5_)Fe(CO)_2_] group, however, have emerged as very versatile intermediates for organic synthesis with potential applications in the area of asymmetric induction [41,286,287,293]. In regard to triphenylphosphine-substituted organoiron complexes including iron-carbene complexes such as [(η^5^-C_5_H_5_)Fe(CO)(PPh_3_) = CHR^+^] it was established [61,63,64,192] that the phosphine ligand (PPh_3_) shields one face of the carbene moiety. Consequently reactions of iron-carbene complexes with nucleophiles can form two diastereomers. The pyramidal nature of the phosphorous atom in organophosphine complexes comprising the PPh_3_ ligand has been reported [293].

Recently Gladysz et al. [294–299] using [η^5^-C_5_H_5_)Fe(CO)(PPh_3_)] or [(η^5^-C_5_H_5_)Re(NO)(PPh_3_)] as chiral auxiliaries carried out numerous diastereoselective and enantioselective transformations which could be of importance in stereoselective synthesis.

### 6.1 Stereoselective Synthesis via Chiral Auxiliary [η^5^-C_5_H_5_)Fe(CO)(PPh_3_)]. Stereoselective Alkylation Reactions

Introduction of the Fe(CO)_3_ group onto a substituted cyclohexadiene makes the molecule asymmetric. Should these complexes be produced in optically active form, any synthesis utilizing them would be asymmetric [300]. Since the demonstration by Brunner [41,301] that the iron acetyl complex [(η^5^-C_5_H_5_)Fe(CO)(PPh_3_) COCH_3_] could be prepared in optically active form and was configurationally stable under ambient conditions, much work has focused on using optically active substrates to gain information about organometallic reaction mechanisms [302,303]. However, the main recent interest in iron acetyl complexes and related metal acyl complexes lies in their novel synthetic applications. Many organometallic reactions proceed through acyl-metal complexes, many of which are quite stable, and readily isolated and handled. With appropriate metal-acyl complexes, protons α to the carbonyl group are acidic, and can be removed by base, to generate the corresponding metal-acyl enolates, i.e., “chiral enolate equivalents” [304]. For example, deprotonation of the stable iron acetyl complex [(η^5^-C_5_H_5_)Fe(CO)(PPh_3_)COCH_3_] with lithium diisopropylamide (LDA) or butyllithium in tetrahydrofuran (THF) at −42 °C generates a deep red-brown solution of a stable iron acyl enolate [305,306], Solutions of this enolate have been shown to undergo a variety of highly stereoselective carbon-carbon bond-forming reactions such as alkylation [307–312], aldol reactions [305,311–317], imine condensation [64,318], Michael addition [319], and synthesis of β-lactams [64].

**Scheme 15:**
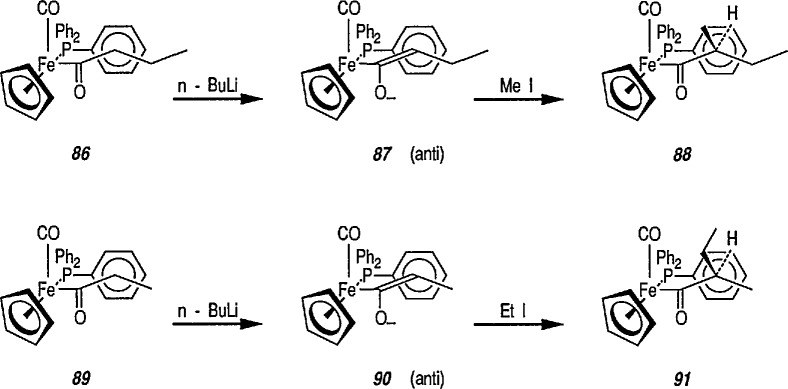


Enolates 87 and 90 derived from acyl ligands attached to the chiral auxiliary [(η^5^-C_5_H_5_)Fe(CO)(PPh_3_)], e.g., complexes 86 and 89, undergo highly stereoselective alkylation reactions [307,308] to give corresponding alkylation products 88 and 91, conversions 86 → 87 → 88 and 89 → 90 → 91 (Scheme 15). The stereochemical control observed in these reactions is consistent with preferential formation of *E*-enolates exclusively (> 200:1) and their subsequent alkylation in the *anti* orientation (O^−^ to CO) from the unhindered face; the other face being completely shielded by the bulky triphenylphosphine ligand [308,309]. The resulting, overall elaboration of a new chiral center via carbon-carbon bond formation also occurs with extremely high stereoselectivity (> 200:1) [310]. Factors controlling the stereoselective alkylation reactions of iron acyl enolates have been discussed [310], and methylation and ethylation of *E*-enolates are depicted in Scheme 15.

A regioselective synthesis of a terminal alkene involving $\text{alkene}-[{\left(\eta^{5}{-C}_{5}H_{5}\right)}^{+}\text{Fe}{\left(\text{CO}\right)}_{2}]{\text{BF}}_{4}^{-}$ complex has been described [320].

A chiral iron acyl of the type (η^5^-C_5_H_5_)Fe(CO)[PPh_2_(C_6_F_5_)]-CO-Me has recently been reported [321] as an effective chiral reagent for stereoselective reactions such as aldol condensations, alkylations or β-lactam synthesis.

**Scheme 16:**
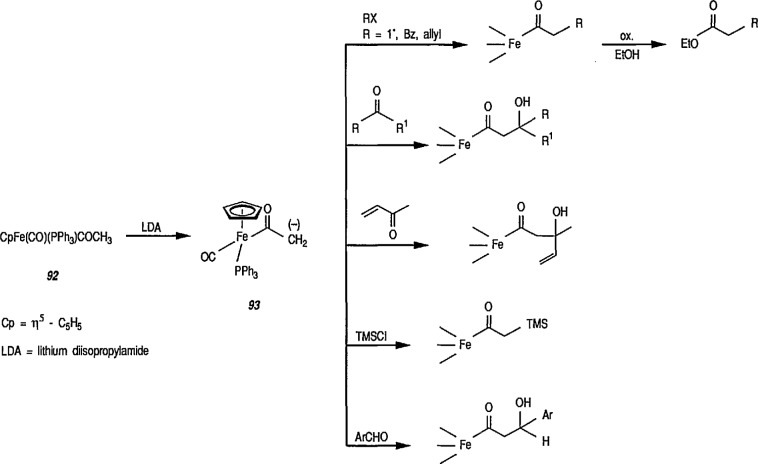


The enolate 93 (from 92) reacts with a wide range of electrophiles to give a series of C-alkylation products [307–317] (Scheme 16). Because acyliron species are oxidatively cleaved to esters, this affords a method of homologation. The stereoselective elaboration of chiral acyliron complexes by addition of electrophiles to the anions combined with known procedures for the resolution [322] and decomplexation of acyl ligands without racemization [323], will allow the development of efficient asymmetric syntheses.

### 6.2 Diastereocontrol of Aldol Reactions via Chiral Iron Acyl Complexes. Stereochemical Effect of Metal Counterions

**Scheme 17:**
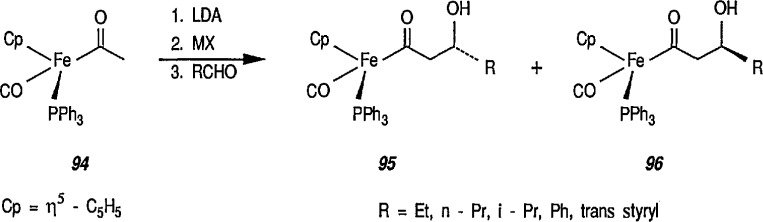


The profound influence of the chiral group [(η^5^-C_5_H_5_)Fe(CO)(PPh_3_)] on the stereoselectivity in a series of conjugate addition reactions has recently been demonstrated. Conjugate additions and conjugate addition-alkylations usually proceed with very high stereoselectivity to α, β-unsaturated acyls of [(η^5^-C_5_H_5_)Fe(CO)(PPh_3_)]. Here, Liebeskind [64,314] and Davies [61,62,311,324] are independently credited for the development of novel chiral reactions of the acetyl iron complex (94). It has been shown [64,314] that the enolate from (94), e.g., [(η^5^-C_5_H_5_)Fe(CO)(PPh_3_)COCH_2_], reacts with aldehydes to afford aldol products 95 and 96 with high stereoselectivity (Scheme 17). In the presence of a Lewis acid catalyst, e.g., *i*-Bu_2_Al*^+^*, as the counterion diastereoisomer 95 preponderated with a 95:96 ratio of up to 8:2 being obtained. With SnCl^+^ as the counterion, the opposite diastereoisomer 96 preponderated with a maximum 95:96 ratio of 1:12.

Davies et al. [311,324] studied exactly this same system, and with the Et_2_Al^+^ counterion, they obtain > 100:1 diastereoselectivity. The origin of this disparity appears to be the use of an excess of alkylaluminum [311]. If Cu(I) is used as the counterion, the opposite stereochemistry is obtained. From these examples it can be seen that the nature of the metal counterion has a profound effect on the stereochemical outcome of the reaction. By changing from an aluminum to a copper or tin enolate, opposite stereoselectivity is obtained. Models to explain these observations have been proposed [64,309].

**Scheme 18:**
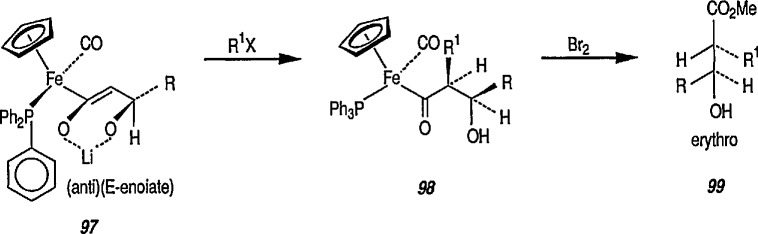


The copper enolate is also reactive toward ketones, again giving aldol products [317]. By generating the dianion 97 of the initial aldol product, and alkylating this enolate (to give 98), followed by oxidative removal of the iron moiety, it was found possible to prepare *erythro* β-hydroxy acid 99 with very high stereoselectivity. Here again the relative configuration of the observed product results from alkylation of the *E*-enolate (97) in the *anti* (O^−^ to CO) orientation from the unhindered face of the complex (Scheme 18).

**Scheme 19:**
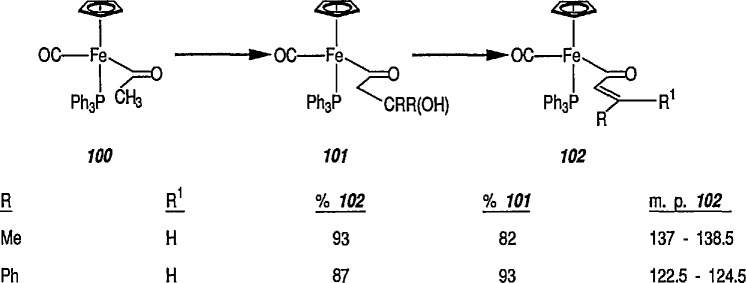


Attempted dehydration of aldol products under a variety of acidic and basic conditions was not successful; however, acetylation of the hydroxyl group of 101 followed by acetate elimination with potassium *tert*-butoxide provided a practical method for preparing quantities of useful α,β-unsaturated iron acyls, e.g., 102, conversion 100 → 101 → 102 [325] (Scheme 19). These intermediates were needed in order to probe the possibility of chiral iron-controlled diastereoselectivity in conjugate addition reactions [326] or Michael additions [326–328].

**Scheme 20:**
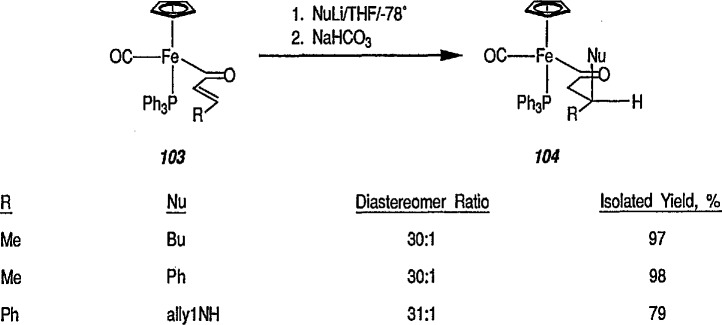


This study has been extended [326] to show that conjugate additions and conjugate addition-alkylations proceed with very high stereoselectivity to α,β-unsaturated acyls of [(η^5^-C_5_H_5_)Fe(CO)(PPh_3_)]. Thus, treatment of *E*-unsaturated acyls 103 (R = CH_3_) or (R = Ph) with lithium nucleophiles NuLi (Nu = Ph, Bu, PhCH_2_NH, PrNH, PhNH, allyl NH, CH_3_OCH_2_CH_2_NH) resulted in an extremely selective conjugate addition reaction, to provide in high yield, practically only one product diastereomer 104, after low-temperature protonation of the intermediate enolate, conversion 103 → 104 [326]. Some stereoselective conjugate addition reactions are shown in Scheme 20.

### 6.3 Stereoselective Synthesis of β-Lactams via Chiral Iron Acyl Complexes

**Scheme 21:**
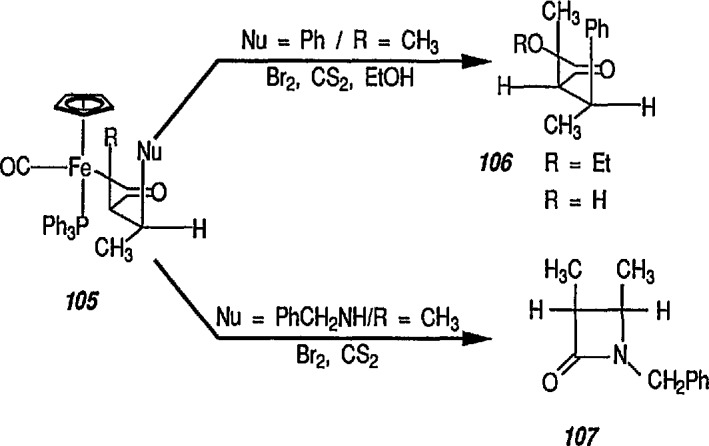


Optically active β-lactams are important antibiotics, and have long been a target of organic synthesis. In all cases, however, a chiral source is essential for enantiometric control, and this can apparently be achieved by using iron acyl complexes. Thus, oxidative cleavage of 105 provides high yields of organic acid derivatives (esters, β-lactams) with almost complete control of the relative stereochemistry. For example, treatment of 105 (Nu = Ph, R = CH_3_) with 1.1 eq of bromine at −78 °C in EtOH/CS_2_ gave the ethyl ester 106a in 83% yield; however, oxidative decomposition (Br_2_/CS_2_/−78°C) of the benzylimine derivative 105 (Nu = PhCH_2_NH, R = CH_3_) without ethanol gave β-lactam 107 in 78% yield. The high-yield, stereospecific formation of β-lactam 107 suggested that the conjugate addition of amine anion nucleophiles to α,β-unsaturated iron acyls followed by alkylation would provide a simple stereospecific route to 2,3-disubstituted β-lactams [64] (Scheme 21).

Both Davies [327,328] and Liebeskind [329] have used this chemistry in a stereoselective synthesis of a series of β-lactams [330]. Davies began with racemic iron acyl complex 108 and its lithium enolate, and condensed it with the phenylimine of benzaldehyde. Only one diastereoisomer 109 is produced, which upon oxidative cleavage (CuCb), produces the β-lactam 110. Similarly, Liebeskind used both the lithium and the aluminum enolates and condensed them with a number of imines. In all cases, mixtures of diastereoisomers are obtained, which upon oxidation (Br_2_ or I_2_/CS_2_) produce the β-lactam in 60–80%yield (table 2). Because the initial chiral iron-acyl complex can be resolved, this in principle, provides an asymmetric synthesis of β-lactams. Bulky β-ainino ironacyls 111, 112 and 113 were also converted into β-lactams (table 2).

**Figure:**
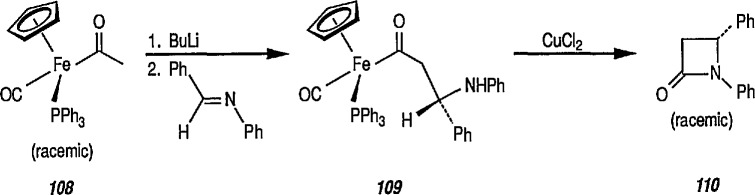


**Scheme 22:**
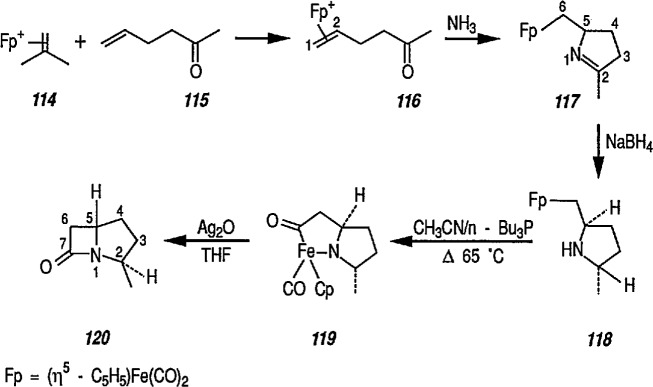


Still another approach to the stereoselective synthesis of β-lactams employing organoiron complexes as intermediates has been described by Rosenblum [331,332]. Thus, allylacetone 115 is converted in high yield into the corresponding π-complex 116 [(η^5^-C_5_H_5_)Fe(CO)_2_(alkene)BF_4_] by exchange with [(η^5^-C_5_H_5_)Fe(CO)_2_ (isobutylene)BF_4_] (114). No competitive complexation of the [(η^5^-C_5_H_5_)Fe(CO)_2_] group with the carbonyl function is observed in this reaction, as the oxygen atom is not sufficiently basic to interfere in the exchange reaction. On treatment with ammonia (CH_2_Cl_2_, room temperature) complex 116 is converted into the pyrroline complex 117. Reduction with sodium borohydride in ethanol affords a 1:1 mixture of stereoisomeric pyrrolidine complexes; only one of which (118) is transformed into chelate complex 119 via a thermal rearrangement (CH_3_CN, three drops of tri-butylphosphine, 65 °C, 7.5 h). Oxidation of the chelate 119 with silver oxide in THF afforded the bicyclic lactam, 2-methylcarbopenam 120 in 72% yield (Scheme 22); oxidation with air can also be used [332].

An alternative new procedure for synthesis of β-lactams has recently been described [292]. Thus, Michael-type additions of amines and thiols to α,β-enoyl-FeCp(CO)_2_,(C_p_=η^5^-C_5_H_5_) (122) (prepared as shown 121 → 122) were carried out without solvent at ambient temperature to give the corresponding new β-aminoalkanoyl-FeCp(CO)_2_ (123) and β-thioalkanoyl-FeCp(CO)_2_ (124) complexes in 60–90% yields (conversion 122 → 123 → 124). These intermediates were then selectively oxidized to β-lactams, e.g., 125. The lactam 125 apparently is a racemate; note the absence of the bulky, electron-donating (asymmetric) triphenylphosphine group in the starting material. Other syntheses of β-lactams are described in section 7 (Natural Products).

**Figure:**
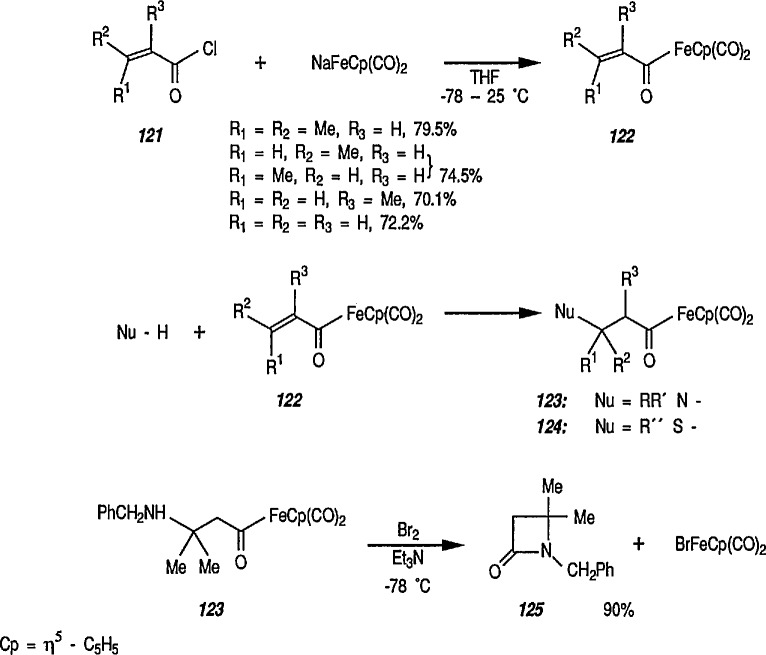


### 6.4 Stereoselective Michael Addition via Chiral Iron Acyl Complexes

**Scheme 23:**
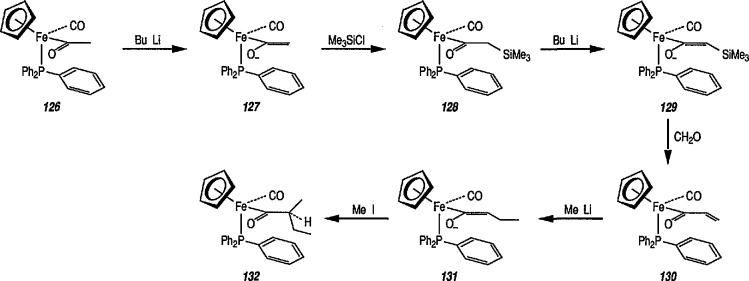


The tandem stereocontrolled formation of two chiral centers via Michael addition of carbanions to iron α,β-unsaturated carbonyl compounds and subsequent alkylation of the enolate thus formed has been recognized for some time [311,319]. It has recently been found [319,328] that α,β-unsaturated complexes of [(η^5^-C_5_H_5_)Fe(CO)(PPh_3_)] undergo tandem stereoselective Michael addition, and subsequent methylation results in the stereocontrolled synthesis of α- and β-substituted iron acyl complexes. Thus, deprotonation of the acetyl complex 126 at −78 °C with butyllithium (BuLi) generated the enolate 127 that, on addition of chlorotrimethylsilane underwent exclusive *C*-silylation to generate the α-trimethylsilyl complex 128 (86% yield). Treatment of 128 in tetrahydrofuran at −78 °C with butyllithium produced the enolate 129 which after addition of a freshly prepared solution of formaldehyde in THF gave the α,β-unsaturated acyl complex 130. Addition of methyllithium to a solution of (130) in THF at −78 °C in the presence of methyliodide stereoselectively (>30:1) generated the (*RS, SR*) diastereoisomer (132) of the (*S*)-butyl acyl complex. The observed preferential formation of (132) is consistent with methyllithium attacking (130) in the *cisoid* orientation to generate the *E*-enolate (131) which, as reported previously, is methylated with high facial stereoselectivity to give (132) [316,317] (Scheme 23).

Thus, α-alkyl- α,β-unsaturated acyl groups attached directly to the chiral iron center (e.g., 126), undergo asymmetric Michael additions to yield *E*-enolates (e.g., 131) which can be trapped to yield quaternary carbon centers highly stereoselectively [319,327]. In case amines are used as nucleophiles then chiral β-aminoacids are formed which can be cyclized to give stereodefined 2,3-disubstituted β-lactams in good yield [328].

Recently, the effect of phosphine substitution on nucleophilic addition to α,β-unsaturated acyliron complexes has been studied by the Herndon group [57]; particularly Michael addition reactions of α,β-unsaturated acyliron complexes where the iron atom is chiral have been examined. The paper concludes [57] that the reaction of organolithium reagents (e.g., R′Li) with α,β-unsaturated acyliron complexes of the type C_p_-(CO)(PR_3_)FeCOCH=CHR′ [C_p_ = (η^5^-C_5_H_5_)] is highly diastereoselective. This high diastereoselectivity can be obtained regardless which phosphine ligand is present at iron; a triphenylphosphine ligand is not required to obtain high diastereoselectivity. Contrary to earlier suggestions conformational preferences of the acyl group in these complexes is clearly due to more than steric interactions between the acyl oxygen and the aryl groups of a triphenylphosphine ligand. More work on this conformational preference is, evidently, in order.

### 6.5 Tricarbonyliron Complexes in Synthesis of 1,4-Diketones

**Scheme 24:**
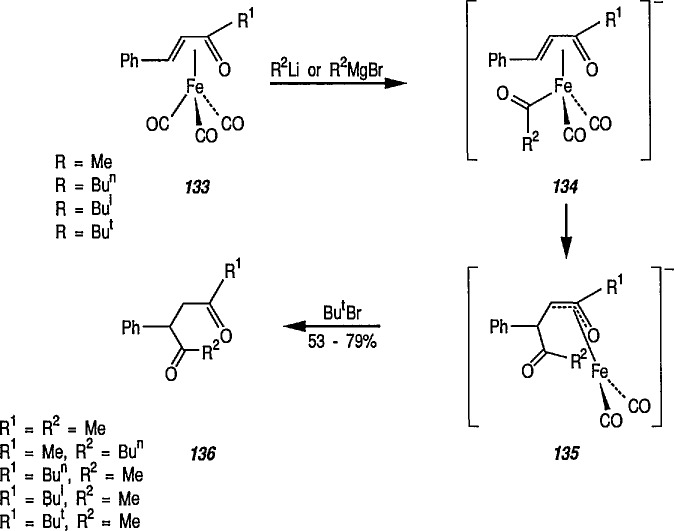


Although tricarbonyliron complexes of α,β-unsaturated ketones were first synthesized more than 20 years ago [333], the reactivity of the α,β-unsaturated ketone fragments of these compounds has received little attention. Electrophilic addition to the complexes has been reported [334,335], but the reaction of tricarbonyliron complexes of α,β-unsaturated ketones with nucleophiles has not yet been investigated. A recent paper [336] fills this gap; the reaction of Grignard and organolithium reagents with iron tricarbonyl complexes of α,β-unsaturated ketones leads to 1,4-diketones in a reaction controlled by the transition-metal center. Thus, tricarbonyl (benzylideneacetone) iron complex 133 was treated with methylmagnesium bromide at −78 °C, and the reaction quenched with *tert*-butyl bromide as a proton source. Removal of the iron residue by filtration through alumina and column chromatography led to isolation of the 1,4-diketone 136 (53–79% yield). The reaction probably proceeds intermolecularly through metal acyl anion intermediates 134 and 135 (Scheme 24). Acyl transfer to the α,β-unsaturated ketone and protonation presumably occur while the α,β-unsaturated ketone is attached to the metal ion. The use of iron tricarbonyl complexes for acyl addition to α,β-unsaturated ketones should have wide synthetic applicability, A recently similar study [337] reports the reaction of tricarbonyliron complexes of α,β-unsaturated ketones with organolithium reagents under an atmosphere of carbon monoxide, to yield α,β-unsaturated ketone-tricarbonyliron complexes in 35–83% yield.

This synthetic reaction can apparently be extended to include iron tricarbonyl complexes of such other Michael acceptors as α,β-unsaturated carboxylic acids and esters, amides, and nitriles.

Recently, the Helquist group [338] published a comprehensive study on highly diastereofacial selective chelation of a phosphite-containing α,β-unsaturated ketone system to the Fe(CO)_2_ group. Conjugate addition to one of these complexes has demonstrated the potential utility of these systems in asymmetric synthesis.

Recently, Brookhart et al. [339] examined the reactions of the (η^3^-allyl) iron tricarbonyl anion, e.g., $\eta^{3}{-C}_{3}H_{5}\text{Fe}{\left(\text{CO}\right)}_{3}^{-}$ with carbon electrophiles such as CH_3_I or PhCH_3_Br and then with PPh_3_, to give α,β- or β-γ-unsaturated ketones. As found for certain substituted allyl systems, e.g., [η^3^-(CH_2_–CH = CHCH_3_) Fe(CO_3_)^−^Na^+^] acyl migration occurred regioselectively to the more hindered methyl-substituted carbon of the π-allyl moiety.

### 6.6. Davies’ Chiral Auxiliary Reagent for Synthesis of Unsaturated Iron Acyls

**Scheme 25:**
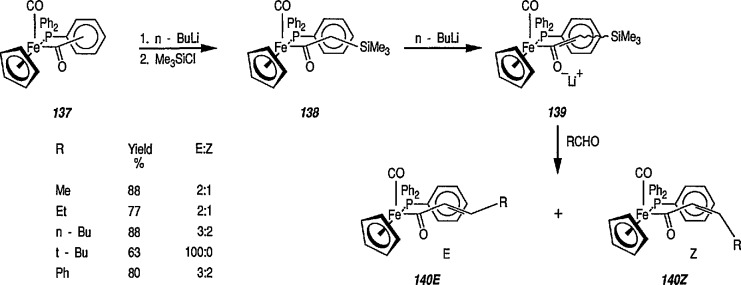


To illustrate the generality of the new methodology for the synthesis of carbon-carbon double bonds [340], the Davies group [61–63, 341] recently expanded the chiral auxiliary Michael-type reaction to include a series of aldehydes. Thus, starting with the chiral acetyl complex [(η^5^-C_5_H_5_)Fe(CO) (PPh_2_)COCH_3_], the authors [341] generated, in high yields, *E* and Z α,β-unsaturated acyl complexes [(η^5^-C_5_H_5_)Fe (CO)(PPh_3_) COCH = CHCH_3_] by using the Peterson alkene synthesis reaction [342] between trimethylsilyl derivatives [(η^5^-C_5_H_5_)Fe(CO) (PPh_3_)COCH_2_Si(CH_3_)_3_] and aldehydes RCHO (R = H, CH_3_, Et, n-Bu, t-Bu, Ph, vinyl, 2-furyl). The procedure involves deprotonation of 137 with butyllithium (THE, −78 °C) followed by trapping of the resulting enolate with chlorotrimethylsilane, to generate the complex 138. Deprotonation of the orange complex 138 with butyllithium (THF, −78 °C) generates the corresponding α-trimethylsilyl enolate 139 which is treated with aldehyde RCHO. Workup by evaporation, extraction with dichloromethane, and filtration through alumina gave a mixture of isomers *E*- and Z-[(η^5^-C_5_H_5_)Fe(CO) (PPh_3_)(COCH = CHCH_3_)] (140*E +* 140Z). The *E* and *Z* isomers are readily separable by chromatography on alumina. Elution with dichloromethane gave the pure *Z* isomer and subsequent elution with 2:3 dichloromethane-ethyl acetate gave the pure *E* isomer (Scheme 25). The ratio of the *E* and Z diastereoisomers was deduced from integration of the methyl doublets and alkene protons in the NMR spectrum. Thus, the stereospecific *syn*-elimination of the Si-O moiety in the base-catalyzed Peterson reaction is an important synthetic procedure for the preparation of α,β-unsaturated acyl complexes.

**Scheme 26:**
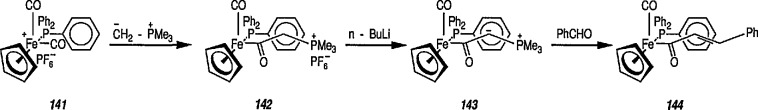


**Scheme 27:**
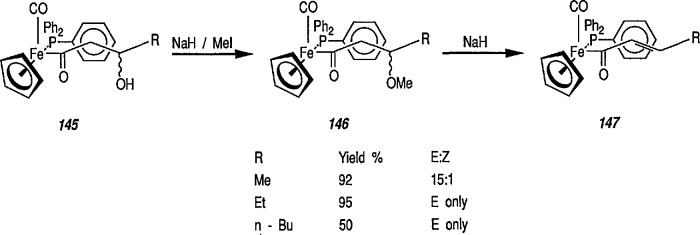


**Scheme 28:**
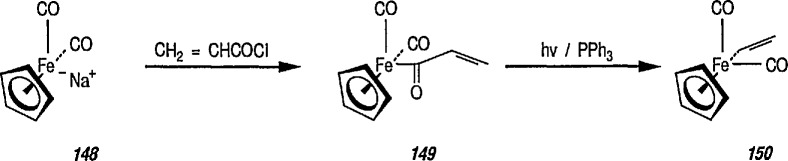


The method [341] was also found useful for a Wittig reaction [340] between phosphoranes and carbonyl compounds for the synthesis of carbon-carbon double bonds. Thus, addition of CH_2_ = P(CH_3_)_3_ to the cation [(η^5^-C_5_H_5_) Fe (CO)_2_ (PPh_3_)]^+^ ${\text{PF}}_{6}^{-}$ (141) in a mixture of tetrahydrofuran and dichloromethane, initially at −78°C and then warming to 20 °C, gave the phosphonium salt 142. Treatment of 142, without isolation, with butyllithium at −78 °C gave the phosphorane 143; on treatment with benzaldehyde this gave α,β-unsaturated iron acyl complex 144 as the simple *E* isomer, albeit in only 25% overall yield. The relatively poor yield of complex 144 by this route may have its origins in the fact that cation 141 is less susceptible to nucleophilic attack than is ${\left[\left(\eta^{5}{-C}_{5}H_{5}\right)\text{Fe}{\left(\text{CO}\right)}_{3}\right]}^{+}{\text{PF}}_{6}^{-}$, due to the presence of the electron-donating triphenylphosphine ligand. Furthermore, nucleophilic addition to cation 141 would be expected to be readily reversible [343] (Scheme 26). Exclusive *E* α,β-unsaturated iron acyl complexes are also formed stereoselectively in high yield via sodium hydride-induced elimination of methanol from the β-methoxy complexes [(η^5^-C_5_H_5_)Fe(CO)(PPh_3_)COCH_2_CH(OCH_3_)R)] (R=H, CH_3_, Et, *n*-Bu, Ph, vinyl, 2-furyl), involving an *O*-methylation-elimination procedure [341], e.g., conversions 145 → 146 → 147. (Scheme 27). Other methods involve reactions of chiral salts with vinyl chlorides followed by photolysis in the presence of triphenylphosphine [341,344,345], conversions 148 → 149 → 150 (Scheme 28).

An alternative synthesis of α,β-unsaturated iron acyl complexes has been advanced [325]; it is a non-stereospecific reaction starting from 3-hydroxy-acyl complexes and proceeding via acetylation and subsequent base-promoted elimination steps.

At present, most commercial applications rely on separating optical isomers from racemic mixtures by such physical processes as fractional distillation, recrystallization, or chromatography [346]. However, ongoing research is opening up prospects for direct asymmetric synthesis of chosen isomers in high optical purity.

Davies’ chiral auxiliaries [60–63] offer stereochemical control of a variety of reactions involving acyl ligands, including enolate and dienolate chemistry, aldol and Diels-Alder reactions, and tandem Michael addition-alkylation reactions. After enantio- and diastereo-controlled reaction, the desired fragment can be liberated under mild conditions.

This technique is applicable to virtually all reactions associated with carbonyl functionality, and can simplify current multistage reaction procedures, giving substantial time saving, e.g., 99+ percent optical purity can be achieved by this direct method [347]. The parent chiral iron acetyl reagent [(η^5^-C_5_H_5_)R(CO)(PPh_3_)COCH_3_] is now commercially available as the (−) *R* or (+) *S* enantiomer or in the (+)(−)-racemate form [347,348] (depicted).

**Figure:**
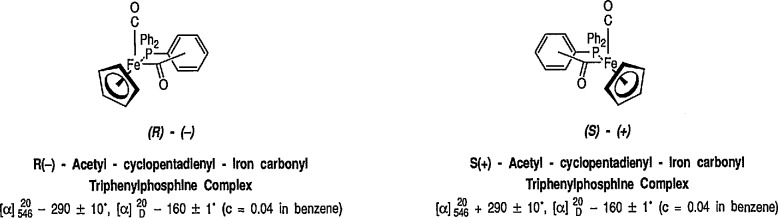


The use of transition-metal-based chirality for asymmetric induction is interesting for two reasons. First, metal-based chirality can occur in a number of geometries inaccessible to organic compounds. Second, steric and electronic perturbation to the inducing chiral center should be achievable with great flexibility in the organometallic system simply by varying the ligands about the metal. Purely organic systems seem inherently less flexible [320].

The results so far accumulated demonstrate the powerful influence that the chiral group [(η^5^-C_5_H_5_)Fe(CO)(PPh_3_)] can have over the stereochemical outcome of reactions that occur under its control; two mechanistic models to explain the factors responsible for the chiral high stereoselectivity have been proposed [320,349]. The most stable conformation for ligands attached to the chiral auxiliary [η^5^-C_5_H_5_)Fe(CO)(PPh_3_)] can be predicted from the NMR analysis [349,350]; this includes recent conformational analysis and x-ray crystal structure of [(η^5^-C_5_H_5_)Fe(CO)(PPh_3_)CH_2_CH_3_] [351] and conformational analysis of [(η^5^-C_5_H_5_)Fe(CO)(PPh_3_)CH_2_CH_3_] (e.g., solvent dependence of conformer populations) [352].

The importance of chiral products has led to a growing interest in asymmetric synthesis including the synthesis of peptides [353] and ferrocenylpyrazolines [354]. For example, three enantiomerically pure α-ferrocenyl-alkylamines were prepared from the natural(−)-menthone via the α-ferrocenyl-alkyl carbocation intermediate; these amines were then used as chiral templates for the stereoselective synthesis of a model compound for peptides [353], The future promises exciting possibilities for the use of stoichiometric chiral auxiliaries, e.g., the chiral template [η^5^-C_5_H_5_)Fe(CO)(PPh_3_)] for asymmetric synthesis. In particular, recent reports have described the self regeneration of chiral auxiliaries [355] so that they are not destroyed during the reactions and may be easily recycled; the paper also reports a new asymmetric synthesis of phenyl alkyl sulfoxides.

### 6.7 Asymmetric Diels-Alder Reactions via Chiral Iron Acyl Complexes

The development of both highly efficient and diastereoselective asymmetric Diels-Alder reactions for the construction of chiral starting materials for organic synthesis has attracted considerable synthetic effort [356,357]. The asymmetric Diels-Alder reaction is a powerful tool for the synthesis of enantiomerically pure, complex molecules.

**Scheme 29:**
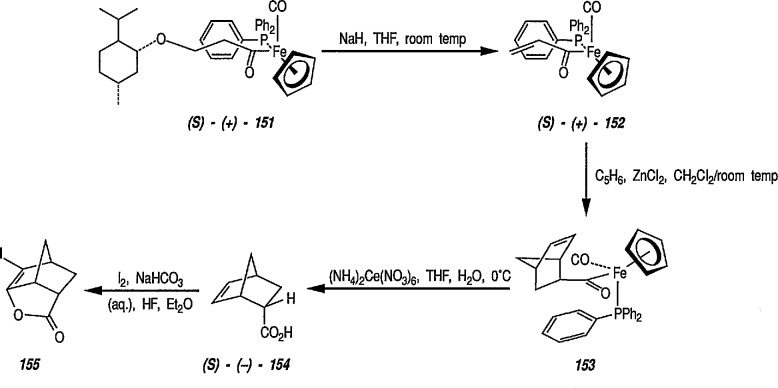


As shown earlier [319,328], *E*-α,β-unsaturated acyl ligands bound to the chiral auxiliary [(η^5^-C_5_H_5_)Fe(CO)(PPh_3_)] undergo highly diastereoselective, tandem Michael additions and alkylations. It was of interest to extend the synthetic potential of α,β-unsaturated complexes to their use as chiral dienophile equivalents in asymmetric Diels-Alder cycloaddition reactions. Recently, Davies and Walker [358] reported such an asymmetric Diels-Alder reaction. Thus, *E*-α,β-unsaturated acyl complex 151 underwent sodium hydride-induced elimination of menthol (THF, room temperature) to give the chiral acrylate dienophile equivalent (S)-(+)-[(η^5^-C_5_H_5_)Fe(CO)(PPh_3_)COCH = CH_2_] (152) $\left[\left(87\%\right),{\left[\alpha \right]}_{D}^{20}+202\left(C,0.11,C_{6}H_{6}\right)\right]$. The Diels-Alder reaction between complex 152 and cyclopentadiene in the presence of one equivalent of zinc chloride at room temperature gave *endo* addition complex 153 as the major diastereoisomer (in addition to two other, minor diastereoisomers). The crude mixture was subjected to ammonium cerium(IV) nitrate oxidation in aqueous THF (0 °C) (decomplexation) to yield, after work-up, preponderantly (2*S*)-(−)-bicyclo[2.2.1]hept-5-ene-2-endo-carboxylic acid (154). The formation of acid 154, whose absolute configuration is known, is consistent with *endo* addition occurring to (*S*)-(+)-(152) in the *cisoid* orientation, the normal reactive conformation of α,β-unsaturated acyl complexes [319], from the face away from the triphenylphosphine ligand. The *endo* enantioselectivity was determined by conversion of acid 154 into the corresponding iodolactone (155) (65% yield). Purification by a single recrystallization gave optically pure $\left(+\right)-155\left\{{\left[\alpha \right]}_{436}^{20}+238.40\left(0.55,C_{6}H_{6}\right)\right\}$. All of the important reaction steps in the conversion 151 to 155 are shown in Scheme 29.

Another new stereoselective Diels-Alder reaction involving α,β-unsaturated iron acyl-difluoride complexes has been reported. A series of Diels-Alder cycloaddition products between dienophiles methacrylate and crotonate derivatives of (ferra-β-diketonato) BF_2_ complexes, e.g., [(η^5^-C_5_H_5_)Fe(CO)]{[H_2_C=C(CH_3_)]CO}BF_2_ and such dienes as isoprene, 2,3-dimethyl-l,3-butadiene, *trans*-2-methyl-l,3-pentadiene, and cyclopentadiene has been synthesized and characterized. Due to the highly asymmetric Fe center within the methacrylate dienophile, diene cycloaddition occurs with unusually high stereoselectivity and regioselectivity [359].

**Scheme 30:**
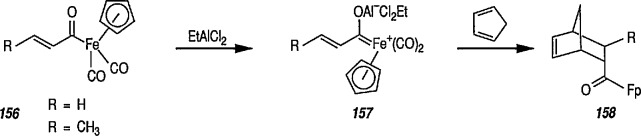


Recently, Diels-Alder reactions between dienes (e.g., cyclopentadiene, isoprene, 3-methyl-l,3-diene) and dienophiles [(η^5^-acryloyl)(η^5^-C_5_H_5_)Fe(CO)_2_] complexes (156) (R = H, R = CH_3_) in the presence of some Lewis acids have been examined, and found to proceed in excellent yields under mild conditions, to give products of high regio- and stereoselectivity [360,361]. Thus, the reaction of 156 (R = CH_3_) (Scheme 30) in benzene at 25 °C with cyclopentadiene in the presence of ethylaluminum dichloride as the catalyst gave the Diels-Alder product (158) in 84% yields. Here, the reacting species, i.e., the aluminum-complexed acyl-metal complex comprising the polarized double bond is best represented by carbene structure (157). In the reaction of the carbene dienophile (157) with dienes, the regiochemistry and stereochemistry observed were consistent with that generally observed in Diels-Alder reactions [362–364]. The preferred mode of addition is endo; particularly, very high endo selectivity was observed in reaction with cyclopentadiene to yield (158) (R = CH_3_) (95:5 endo:exo) while only modest endo selectivity was observed with isoprene (77:23 endo:exo) (81% yield), conversion 159 → 160 (R = CH_3_) [360].

**Figure:**
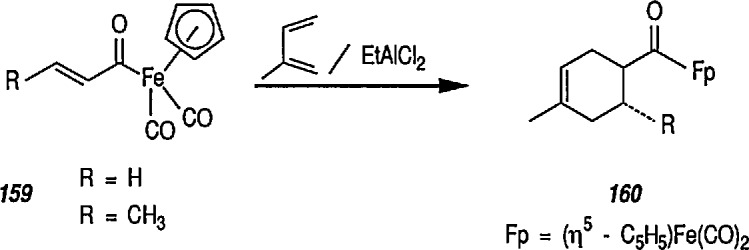


**Scheme 31:**
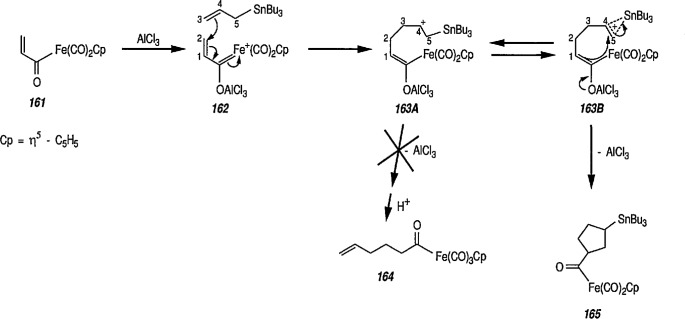


The author extended this study, and examined the reaction of allylstannanes with α,β-unsaturated acyl iron complexes, e.g., 161; a novel [3 + 2] cycloaddition was observed [361]. As shown in Scheme 31, this process, catalyzed by aluminum chloride, does not give the expected 5-hexenoyl species 164, but provides the unexpected five-membered-ring adduct 165. This reaction is an example of a novel and useful method for cyclopentanoid synthesis; numerous preparations were described. The proposed mechanism for [3 + 2] cycloaddition is outlined in Scheme 31 [361]. First, the aluminum chloride complexes with the acyliron, giving the carbene complex 162. The allylstannane then attacks the electrophilic carbene complex at C-2, yielding the intermediate tin-stabilized carbocation 163a ⇆ 163b. Attack by the enolate at C-5 gives the five-membered-ring compound 165. Here, iron donates significant electron density to the enolate in 163, making it more reactive and making ring closure faster than with other enolates.

### 6.8 [(η^5^-C_5_H_5_)Fe(CO)_2_(η^1^-C_5_H_5_)] a Useful Synthetic Equivalent of Substituted 1,3-Cyclopentadienes in Cycloaddition Reactions

**Scheme 32:**
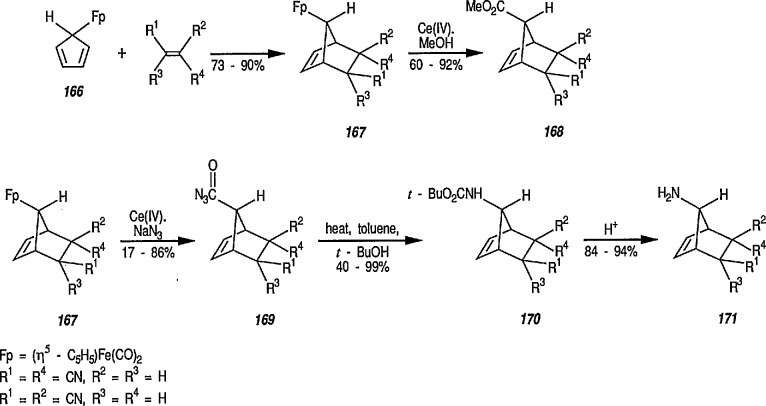


A new, useful approach for the stereoselective synthesis of substituted 2-norbornenes has recently been developed using Diels-AIder cycloaddition reaction. The reaction of [(η^5^-C_5_H_5_)Fe(CO)_2_(η^1^-C_5_H_5_)] (166) with a variety of unsaturated compounds to give cycloadducts 167 in good yield has been reported [365,366]. These reactions all occur regio- and stereoselectively to afford 7-syn-[(η^5^-C_5_H_5_)Fe(CO)_2_] cycloadduct 167 as shown in Scheme 32. Furthermore, stereospecific replacement of the [(η^5^-C_5_H_5_)Fe(CO)_2_] moiety in these adducts by a CO_2_CH_3_ group with retention of configuration to give 168 was found to occur in good yield by oxidation with ammonium cerium(IV) nitrate in methanol saturated with carbon monoxide [365,366]. This two-step sequence, cycloaddition followed by oxidation, renders [(η^5^-C_5_H_5_)Fe(CO)_2_(η^1^-C_5_H_5_)] a synthetic equivalent of methyl l,3-cyclopentadiene-5-carboxylate in cycloaddition reactions. It was subsequently found [367] that, when the oxidation of 167 was performed with ammonium cerium(IV) nitrate (or bromine or chlorine) in acetonitrile containing sodium azide, the product was acyl azide 169 in which the CON_3_ group replaced the [(η^5^-C_5_H_5_)Fe(CO)_2_] group with retention of stereochemistry in good yield. Thermolysis of the acyl azide 169 in a toluene-*tert*-butyl alcohol (2:1) solution at reflux produced, via Curtius rearrangement, the corresponding carbamate 170 in excellent yield; the reaction also proceeded stereospecifically. Selective hydrolysis (*p* -toluenesulfonic acid, acetonitrile, 25 °C, 15 h) of carbamate 170 yielded new 7-*syn* -amino-2-norbornene 171 in 84–94% yield also with retention of stereochemistry (Scheme 32). This reported synthesis of substituted 7-*syn* -amino-2-horbornenes provides an attractive route to these new compounds and substantially expands the known methodology for the synthesis of stereospecific norbornenes.

Some recent reports [368,369] describe several instances in which iron complexes having planar chirality have been utilized with complete stereocontrol as equivalents of cyclohexanone cation synthones. In extension of this study [370], some uses of iron resolved complexes as chiral intermediates to 5-substituted 2-methylcyclohexanones required in terpene synthesis have been described; a general approach when applied inorganic enantiomer synthesis was also outlined.

### 6.9 Stereoselective Syntheses of Coordinated Phosphines

Recent results have shown [371] that metal complexes can be highly effective resolving agents, protecting agents, and chiral auxiliaries for stereoselective synthesis of macrocyclic quadrldentate tertiary arsines. For example, highly selective alkylations of terminal phosphido-metal group (M-PX_2_, where M = Fe, Cr, etc.) are required for syntheses of macrocyclic poly(secondaiy and tertiary phosphines) on metal ions in order to avoid separations of complex mixtures of diastereoisomeric product [372]. This is supplemented by recent findings [373] that asymmetric tertiary phosphido-metal group Fe-PMePh can be generated stereospecifically, first by deprotonation of [(R*,R*),(R*)-(η^5^-C_5_H_5_){1,2-C_6_H_6_ (PMePh)_2_FeHMePh]PF_6_ with potassium-*tert*-butoxide at −90°C, followed by alkylation (iodomethane) to give kinetic products [(R*,R*),(R*)] or [(R*,R*)(S*)] in >99% diastereoisomeric excess. These results auger well for stereo-selective synthesis of poly(secondary or tertiary phosphines) on metal ions.

## 7. Synthesis of Natural Products via Iron Carbonyls

### 7.1 Synthesis of Alkaloid (±)Limaspermine. The Pearson Synthetic Approach via Cyclohexadienyliron Complexes

Iron-stabilized carbocations as intermediates for organic synthesis, particularly the synthesis of natural products and the construction of molecules that might be useful for studying certain organic reactivity phenomena, have been developed for Pearson [21,110,117]. A few examples directed at the synthetic application of dienyl cations that are stabilized by their attachment to an iron(O) moiety, usually the Fe(CO)_3_ group, are discussed next.

**Scheme 33:**
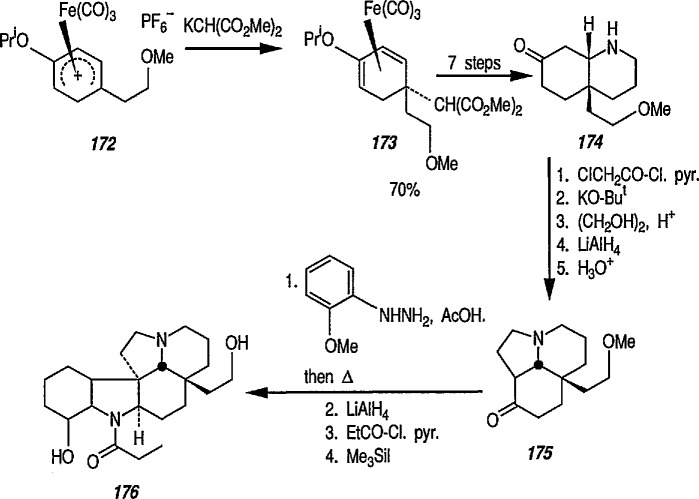


**Scheme 34:**
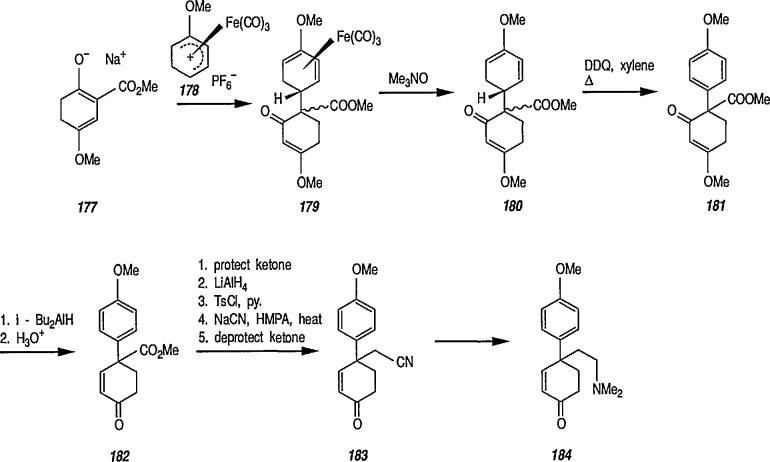


An application of cyclohexadienyliron complexes is exemplified by the total synthesis of the alkaloid (±)-limaspermine 176 (Scheme 33) in the racemic form [110,374]. The synthesis demonstrates successful application of regiocontrol, and illustrates the remarkable stability of the diene-Fe(CO)_3_ unit toward a wide range of chemical transformations, such as decarboxylation and homologation; indeed, the Fe(CO)_3_ allows functional-group interconversions on the side chain that would be troublesome to perform in its absence. The synthesis begins with the readily prepared isopropoxy-substituted (favorable directing effect) dienyl-Fe(CO)_3_ cation 172 (compare Scheme 34). On treatment with a regiospecific nucleophile such as the potassium enolate of dimethyl malonate [i.e., KCH(CO_2_Me)_2_] the cation 172 gave a mixture containing mainly the *cis* isomer 173 (10:1 ratio) from which pure 173 was obtained in high yield by simple recrystallation. Complex 172 was transformed, as shown in Scheme 33, by a multi-step procedure [374] to the decahydroquinoline 174, again illustrating the remarkable stability of the diene-Fe(CO)_3_ unit toward chemical transformations. The decahydroquinoline intermediate 174 was then converted into (±)-limaspermine 176 by the sequence illustrated (conversions 172 → 176). This constituted the first total synthesis of a complex natural product (five rings, four chiral centers) from organoiron precursors. A new synthetic approach to Aspidosperma alkaloids related to limaspermine 176 involving construction of the important quaternary C-20 carbon, via the cyclohexadienyl-Fe(CO)_3_ intermediate, has been advanced [375].

### 7.2 Synthesis of Alkaloid(±)-*O*-Methyljoubertiamine and Other Natural Products via Aryl Cation Equivalents

The addition of carbon nucleophiles to simple cyclohexadienyl-Fe(CO)_3_ cations also provided an opportunity to examine the potential of these complexes as aryl cation equivalents [376] suitable for application to total synthesis. This was demonstrated by the synthesis of alkaloid (±)-*O*-methyljoubertiamine 184 [117,118,377] (Scheme 34). Thus, reaction of sodium enolate 177 with the dienyl complex 178 (readily prepared from anisole) gave the adduct-complex 179 (92% yield). Decomplexation of 179 followed by treatment of the resulting dienol ether 180 with DDQ in boiling xylene generated the aromatic key intermediate 181. The further sequence as shown in Scheme 34 generated enones 182 and 183; earlier [378] the enone 183 had been converted into alkaloid 184, thereby accomplishing a formal synthesis of 184.

**Scheme 35:**
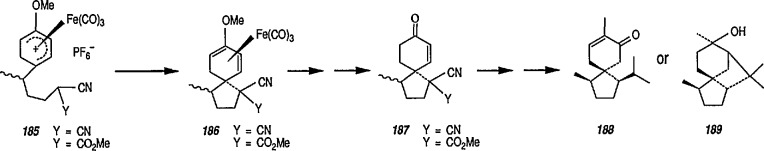


**Scheme 36:**
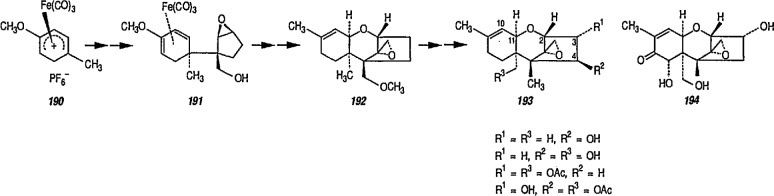


Similar cyclohexadienyl cations were applied in the synthesis (via spirocyclization) of such natural terpene analogs as acorenone (188) or cedrol (189) (conversions 185 → 188 → 189) [117,119] (Scheme 35). The total synthesis of sesquiterpene thrichothecene analogs 193 or 194 in which the initial carbon-carbon bond-forming step involved reaction between stabilized metal enolates and tricarbonyl(4-methoxy)-1-methylcyclohexadienylium)iron hexafluorophosphate electrophile (190) has also been reported [95,120] (conversions 190 → 193 → 194) (Scheme 36). Using cyclic and acyclic tributyltin enolate (instead of lithium enolate and silyl enol ether) for effecting the C–C bond-formation, a new diastereoselective synthesis of sesquiterpene (±) trichodiene or (±) trichodermol has been described [379]. The sesquiterpene hydrocarbon trichodiene is the biosynthetic precursor of the trichothecenes, a class of over eighty fungal metabolites [380].

The foregoing procedure demonstrates that efficient synthesis of complex, *para*-substituted anisole derivatives is extremely simple using organoiron precursors [376], and that the resulting cyclohexadiene-Fe(CO)_3_ complexes can be used in an alternative approach to natural-product syntheses [89,113,381–383], for example, for the synthesis of quassinoid-type natural terpenes having high antitumor activity [384]. The total synthesis of the natural diterpene aphidicolin (active against herpes virus) involves cyclocarbonylation with sodium iron tetracarbonyl [385].

Unfortunately, the use of cyclohexadienyliron complexes such as 172, 185 or 190 in natural product synthesis is limited by the fact that these molecules possess a plane of symmetry, so that the products 176, 188 or 193 were unavoidably obtained in racemic form. A recent work by the Pearson group [385a] offers a possible new approach to non-racemic natural-product synthesis. Thus, addition of chiral *N*-acyloxazolidinone enolates to dienyl-iron complexes (of type 172,185 or 190) or to diene-molebdenum complexes results in asymmetric induction and gives enantiomeric excess as high as 80%. This method offers promise over other methods in that the oxazolidinones give acceptable high enantiomeric excess as well as a recoverable chiral auxiliary. Furthermore, coupling of this synthetic method with previously established manipulations of the resultant π-alkyl-molybdenum and diene-iron complexes [95,120,374,379] provide a valuable tool for asymmetric synthesis of natural products.

### 7.3 Synthetic Applications of Cycloheptadienyliron Complexes to Natural Products

From the synthetic point of view, it is difficult to determine which of several available conformations for polysubstituted cycloheptane derivatives is the preferred, lowest-energy form [386]. However, this circumstance can be overcome via controlled functionalization in the cycloheptane ring [114,121,131,387–389]. For example, methods for functionalization of cyclopheptene and cycloheptadiene derivatives using a transition-metal moiety, e.g., the Fe(CO)_3_ group, as a means of introducing conformational rigidity and achieving stereocontrolled C–C bond-formation has been explored by the Pearson group [95,114,121,122]. The attachment of a transition-metal moiety to an alkenic (or dienyl) ligand offers a unique means of attaining stereospecificity during a variety of chemical transformations and C–C bond-forming processes [89,116]. The cycloheptadienylmetal system is ideally suited for 1,3-stereocontrol, but inspection of the literature reveals that the reactivity of cycloheptadienyl-Fe(CO)_3_ complexes, e.g., 199, bears little resemblance to that of six-membered-ring counterpart. New approaches for functionalization [390,391], or breaking [392], of cycloheptatrienyliron complexes have been reported.

Recent efforts have been made [387–389, 393] at using cycloheptadienyliron complexes as precursor to the stereocontrolled construction of acyclic fragments of the macrolide antibiotics magnomycin B 195 [121] and aglycon tylonolide 196 [121,393]. Similarly, the strategy was designed [388,393] to use cycloheptadienyl-Fe(CO)_3_ complex for the synthesis of the right-hand sections of the 16-membered-ring macrolide antibiotics tylosin 197 and carbomycin B 198 [388,393].

**Scheme 37:**
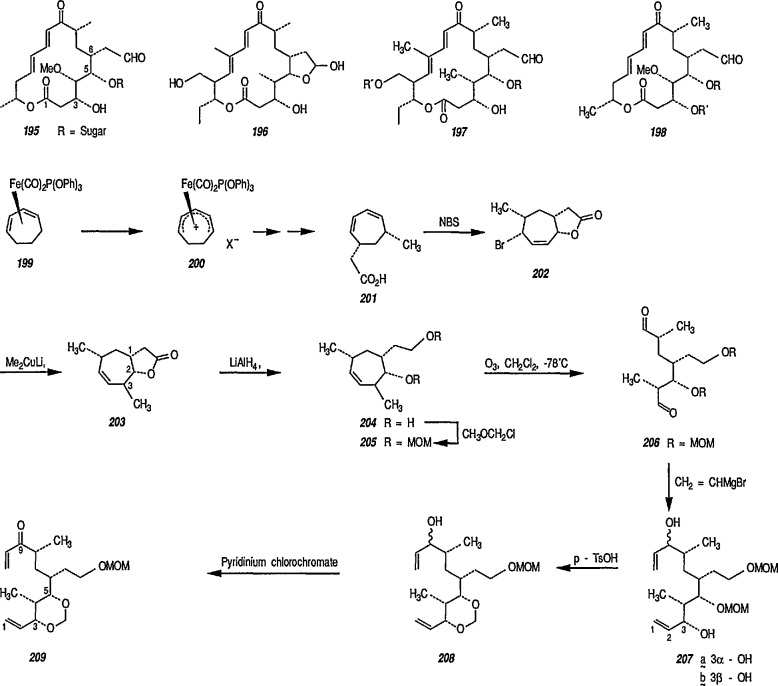


Construction of acyclic fragments of antibiotics 197 and 198 begins with cycloheptadiene 199, which is converted into cycloheptadienyl-Fe(CO)_3_ cation 200. A successive nucleophile addition, followed by demetalation, leads to the racemic carboxylic acid 201, and this, on treatment with *N*-bromosuccinimide (NBS), is converted into the sensitive lactone 202, representing C-3 and C-9 sections of tylosine 197 and carbomycin B 198. Conjugate *anti*-displacement of bromide, followed by ozonolysis, treatment with vinylmagnesium bromide and then with *p*-toluenesulfonic acid, and oxidation, produced enone 209, obtained as a single diastereoisomer representing the C-1-C-11 subunit of tylosine 197; see conversions 199 → 209 (Scheme 37).

The use of organoiron methodology, coupled with manipulation of the product diene, thus provides a potentially flexible approach to macrolide syntheses, and progress in this direction is continuous.

Free-radical coupling-reactions of organoiron complexes as a potential tool for stereoselectivity in synthesis has recently been recommended [394].

### 7.4 Natural-Product Syntheses via the Polybromo Ketone-Iron Carbonyl Reaction. The Noyori Synthesis

Transition-metal carbonyls have been used widely, both as synthetic reagents and catalysts, and the development of significant synthetic methods via such complexes is still continuing [395–397], The involvement of transition metals, and particularly, of organoiron complexes, in synthesis of natural products is a new, challenging field. A current synthetic approach involves either Fe(CO)_3_-substituted intermediates or use of iron carbonyls as specific reducing agents.

#### 7.4.1 Reaction of the Noyori Intermediate with Alkenes and Dienes

A new synthetic methodology using iron carbonyls, e.g., Fe(CO)_5_ or Fe_2_(CO)_9_ (better reducing agent) as reducing agents in the reaction with polybromo ketones to give cyclic ketones has been developed by Noyori [45,46,253,254]. Thus, the iron carbonyl-promoted, cyclocoupling reaction of polybromo ketones and unsaturated substrates provides a powerful tool for the synthesis of five- and seven-membered carbocycles, and many of them are useful intermediates in the synthesis of alkaloids, terpenes, and related natural products [45,251,398]. Mechanistic investigation has provided many lines of evidence for the reduction initiated by two-electron reduction of the dibromo ketone 210 (directly or via oxidative addition of the C–Br bond), to give the enolate 211, which then eliminates the allylic bromine atom to produce the oxyallyl-iron(II) complex [399,400] 212.

**Figure:**
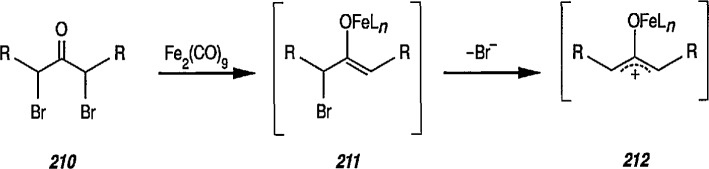


**Scheme 38:**
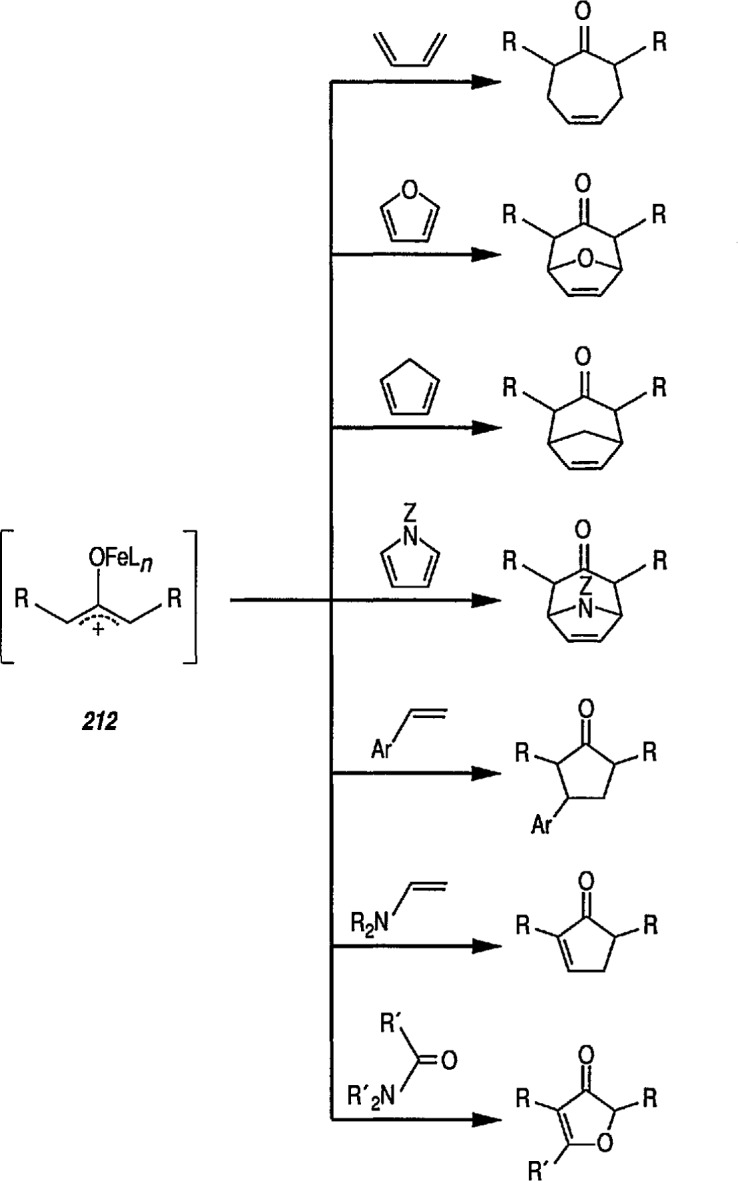


The unique reactivity of this new type of allylic dipolar species, e.g., 212, acting as both a uni- and a bi-functional, three-carbon electrophile, relies heavily on the presence of the central oxygen group. It has been found [45] that parent ketones thus functionalized can undergo cycloaddition with a variety of unsaturated substrates. Scheme 38 illustrates various types of valuable reactions. For instance, 212 underwent [3 + 4] cycloaddition with open-chain dienes, producing substituted 4-cycloheptenones [401]. In addition, the cycloaddition of cyclic dienes including cyclopentadiene [401,402], and heteroaromatics such as pyrroles [401,403] and furans [401,404], gives bridged ketone systems. The reaction with furans proceeds by a concerted process, with the regioselectivity controlled by the frontier molecular orbitals of the two reactants [405]. The oxyallyl cations not only react with dienes but also with certain alkenes in a [3 + 2] manner, producing five-membered-ring ketones [406,407]. The Noyori methodology [408] has recently been extended to synthesis of a series of such natural products as *C*-nucleosides, alkaloids, terpenes, and other classes of complex organic compounds.

#### 7.4.2 Synthesis of *C*-Nucleosides

**Scheme 39:**
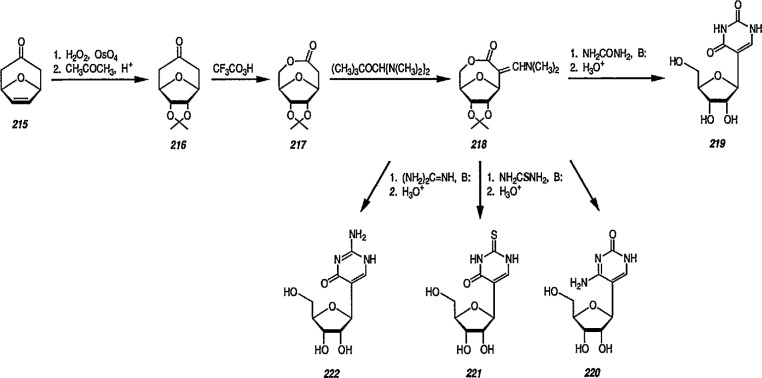


The successful use of *sym*-tetrabromoacetone as a C_3_ unit in the iron carbonyl-aided, [3 + 4] cyclocoupling process has opened a new route to *C*-nucleosides [408]. Thus, the reaction of α,α,α′,α′-tetrabromoacetone 213 and Fe_2_(CO)_9_ in furan gave the adduct 214 in 63% yield, and upon brief treatment with Zn/Cu couple in methanol, compound 214 was quantitatively converted into oxabicyclic ketone 215. When 215 was subjected to osmium tetraoxide-catalyzed dihydroxylation using *tert*-butyl hydroperoxide, followed by acetonation, the oxygen functions were introduced to the double bond solely from the less-hindered side, to give the isopropylidene 216 as a single isomer in 68% yield. Bayer-Villiger oxidation of 216 with trifluoroperoxyacetic acid afforded the lactone 217 in 81% yield. The key compound 217 thus obtained has an adequate *C*-β-glucosyl structure and serves as the precursor of various natural and synthetic *C*-nucleosides. For example, reaction of the optically active lactone 217 with *tert*-butoxyl-bis(dimethylamine)methane produced the α-dimethylaminomethylene lactone 218, a common synthetic intermdiate for pyrimidine *C*-nucleosides, in 91% yield. Thus, condensation of 218 with urea in ethanolic sodium ethoxide, followed by removal of the isopropylidene protecting group, afforded pseudouridine 219 (60% yield). As shown in Scheme 39, the isopropylidiene lactone 218 was converted into pseudocytidine 220, and 2-thiopseudouridine 221 (60% yield), and into chematherapeutically active pseudoisocytidine 222 (70% yield) [409,410]. Thus, the highly chiral *C*-nucleosides were prepared from simple, achiral materials, namely, acetone and furan. Most of the synthetic approaches presented so far are based on introduction of the heterocyclic base into the ribose anomeric center, and do not allow strict stereochemical control [262–266,271,411–413]. Furthermore, because the key [3 + 4] cyclocondensation is applicable to a wide range of polybromo ketones and furans, this approach is capable of preparing various kinds of artificial *C*-nucleoside analogs, particularly those having unnatural carbohydrates possessing alkyl or hydroxyalkyl substituents at appropriate positions [414]. Homo-*C*-nucleosides were obtained by similar synthetic operations [415].

**Figure:**
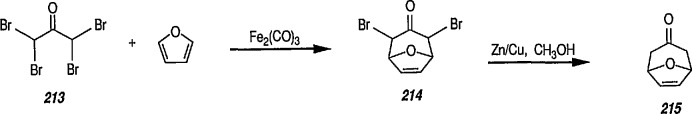


#### 7.4.3 Synthesis of Tropane Alkaloids

**Scheme 40:**
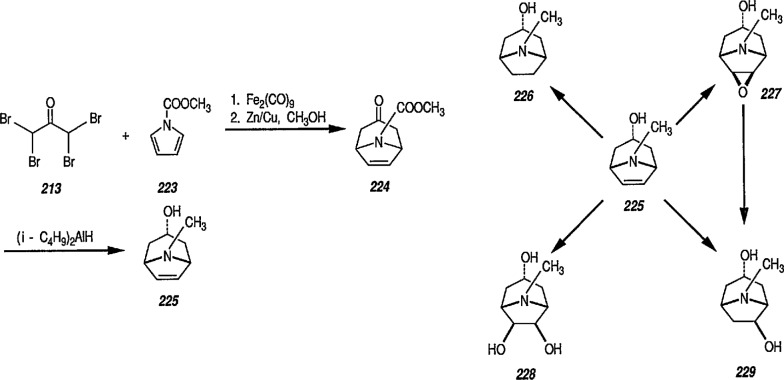


The utility of [3 + 4] cycloaddition reactions was applied for synthesis of tropane alkaloids having the 8-azabicyclo[3.2.1]octane system [416]. Thus, when tetrabromoacetone 213 was treated with Fe_2_(CO)_9_ in the presence of *N*-(methoxycarbonyl)pyrrole 223, followed by the action of a Zn/Cu couple in methanol, the azabicyclic ketone 224 was obtained in 57% yield. Reduction of 244 with diisobutylaluminum hydride gave stereoselectively 6,7-dehydrotropine 225 (having the natural α-hydroxyl function) in 92% yield (α:β ratio 93:7) [417]. The carbamate moiety was reduced to *N*-methyl at the same time. The alcohol 225 is convertible through appropriate reductive or oxidative modification of the double bond into most of the naturally occurring tropane alkaloids, such as tropine (226), scopine (227), tropanediol (228), and teloidine (229) [418] (Scheme 40).

#### 7.4.4 Synthesis of Terpenes

**Scheme 41:**
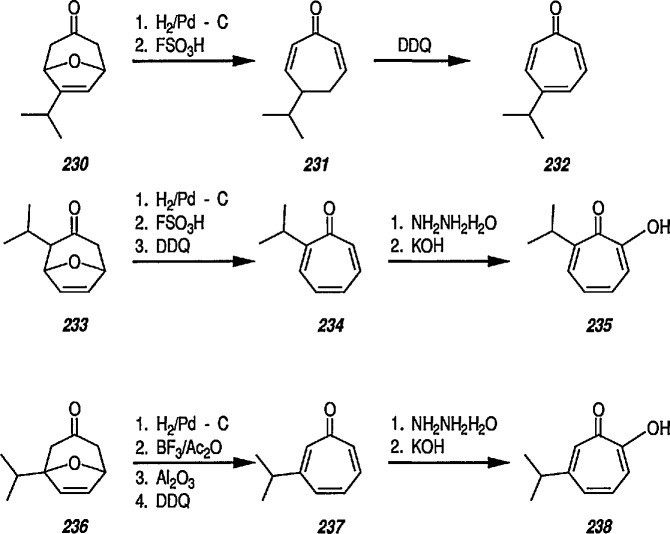


The new method for cyclocoupling was further applied to the syntheses of terpenoids and related compounds. An example is the synthesis of naturally occurring troponids, achieved via a sequence of simple reactions starting from [3 + 4] adducts of the polybromo ketones and furan derivatives [419]. Thus, nezukone (232) was conveniently prepared by using the starting material, compound 230, obtainable from tetrabromoacetone and 3-isopropylfuran, through its hydrogenation on Pd/C, and dehydration with fluorosulfonic acid, giving the cross conjugated dienone 231, and then dehydrogenation using 2,3-dichloro-5,6-dicyano-1,4-benzoquinone (DDQ) [419,420]. In similar fashion, the bicyclic adduct 233 was converted into 2-isopropyltropone 234, and its α-hydroxylation according to the standard method (α-amination with hydrazine hydrate followed by basic hydrolysis) produced α-thujaplicin 235 [410,419]. Hinokitol (β-thujaplicin)(238) was synthesized via a similar reaction sequence involving (236) and (237) as the key intermediates (see Scheme 41). The natural tropolone β-thujaplicin 1X has also been synthesized via Friedel-Crafts acylation of the tropone-Fe(CO)_3_ complex [421]. Terpenes having a bicyclo [2.2.1] heptane skeleton, e.g., (±)-campherenone, are conceived to be biosynthesized by the double cyclization of the appropriate allylic cation. The iron carbonyl-promoted, intramolecular [3 + 2] process provided a chemical analog of this bioconversion [422,423].

More syntheses of terpenes via the polybromo ketone-iron carbonyl reaction have been described [407].

#### 7.4.5 Other Related Syntheses

**Scheme 42:**
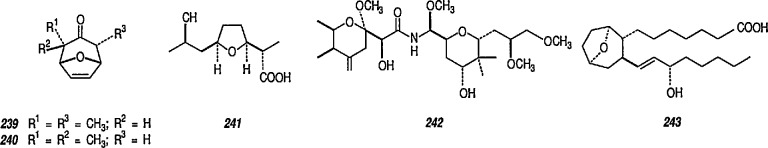


There have also been achieved in syntheses of other natural products and their analogs via the [3 + 4] reaction of the polybromo ketones and furan as the key step. (±)Nonactic acid 241 [409] was synthesized [424] through 239 and 240 [409]. The ketones 239 [409] served for the construction of the right-hand block of (±)-pederin 242 [425]. Preparation of a thromboxane A_1_ analog 243 [426] was accomplished by starting from 215 [409] (Scheme 42).

A cycloaddition reaction between 2-cyanodimethylfumarate and (σ-3-methoxyallyl)(η^5^-C_5_H_5_) dicarbonyliron yielded a cyclopentanoid derivative, used in a synthesis of the antitumor agent sarkomycin [427].

### 7.5 Synthesis of the β-Lactam Antibiotic (+)-Thienamycin via an Intermediate π-Allyltricarbonyliron–Lactone Complex

Although the importance of β-lactam antibiotics has been recognized for many years [330,428–431], the recent discovery of some structurally new types of compounds [432–437] has generated a flood of interest in their methods of synthesis. Of the many novel routes to the azetidinone ring inherent in these systems, the use of iron carbonyl complexes is an attractive and growing area of study [327,329,332,438].

π-Allyltricarbonyliron lactone complexes have been used as novel precursors for the synthesis both of natural β-lactones, e.g., parasorbic acid (a bee pheromone) [439] and β-lactams [440–442]. Indeed, π-allyltricarbonyliron lactone complexes can be the key synthetic intermediates [440–442] to the important lactam antibiotics, such as (+)-thienamycin (255) (having a broad antibacterial activity) [443]. The synthesis of the lactam 255 [442] utilizes the reaction of vinyl epoxides with coordinately unsaturated iron carbonyl species to afford a precursor π-allyltricarbonyliron lactone complex 246 [441]. Preparation of the substituted vinyl epoxide (245) necessary was achieved in 55% yield by reaction of 3,3-dimethoxypropanal with dimethyl-(2-oxopropyl) phosphonate, to give the enone 244, followed by methylenation with dimethylsulfonium methylide [445]. Conversion of 245 into the tricarbonyliron lactone complex 246 was possible by treatment with Fe(CO)_5_ under photolytic conditions. Reactions of the lactone complex 246 with the chiral amine (*S*)-(−)-β-methylbenzylamine, mediated by ZnCl_2_, proceeded slowly, to give two readily separable diastereoisomeric ferrilactam complexes, i.e., 247 and 248 in 29 and 30% yield, respectively. Independent oxidation of the diastereoisomers 247 and 248 with eerie ammonium nitrate gave the *cis*-fused lactams 249 and 250 in 87 and 88% yields, respectively. Ozonolysis of the isopropenyl-substituted β-lactams (249 and 250) proceeded readily, to afford the 3-acetyl derivatives (251 and 252), each in 81% yield. Reduction of the acetyl group in 251 and 252 could be achieved with high stereoselectivity by using potassium tri-*sec*-butylborohydride (K-selectride) in diethyl ether at room temperature, to give *trans*-(erythro-hydroxyethyl) derivatives in good yield. Debenzylation of these intermediates with sodium in liquid ammonia gave the desired enantiomeric (hydroxethyl)-β-lactams 253 and 254 in excellent yields, with optical rotations ${\left[\alpha \right]}_{D}^{22}$ + 11.4° and −10.7°, respectively.

**Scheme 43:**
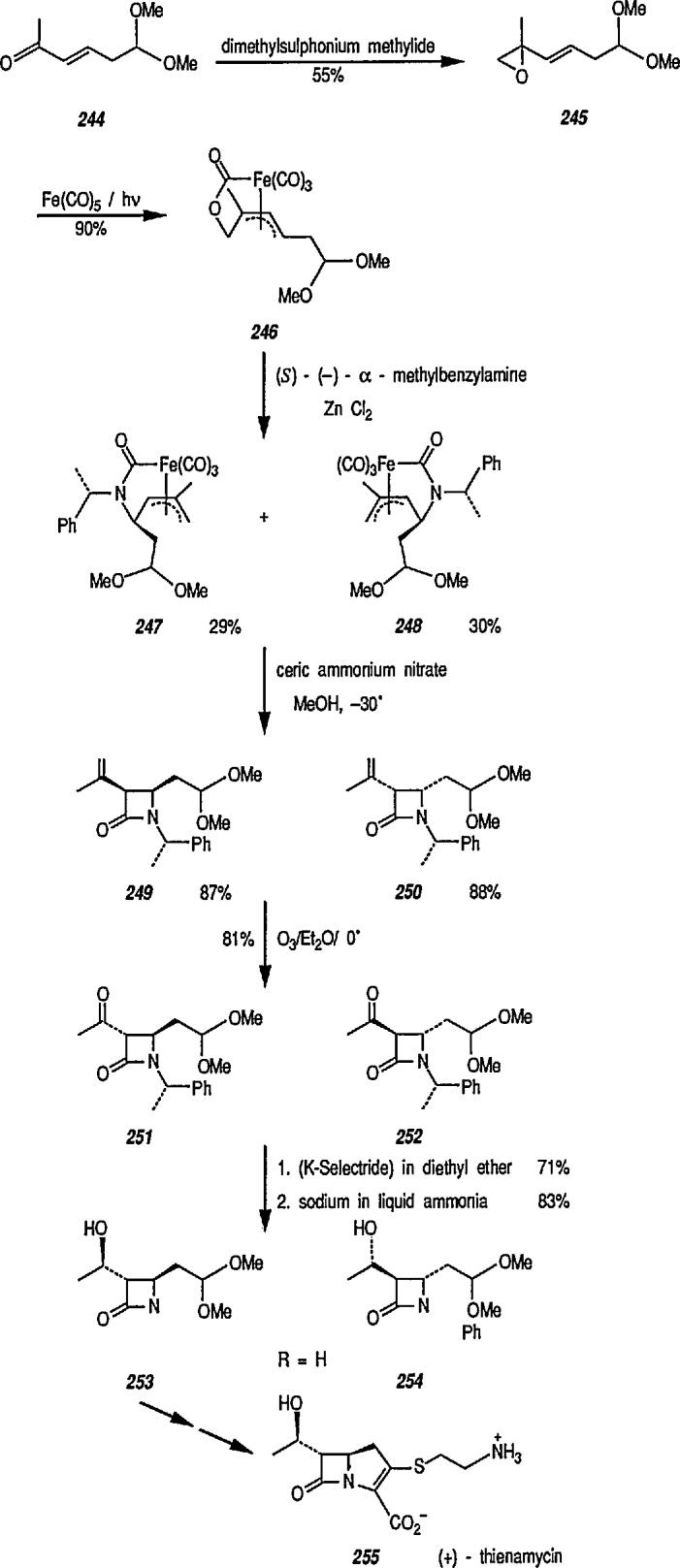


Applying the procedure of Kametani [446], the optically active lactam 253 can be converted in eight steps into thienamycin 255 in its naturally occurring (+)-form; this work, therefore constitutes a formal total synthesis [444] (Scheme 43). The novel route just described is reasonably short, and may be further modified and developed [442] for the synthesis of a wide range of β-lactam antibiotics.

### 7.6 Highly Enantioselective Synthesis of Leucotriene B_4_ and Its 14,15-Didehydro Derivative by the Use of Butadiene Tricarbonyl-Iron Complex

Leucotriene B_4_ (LTB_4_) (261) is the major proinflammatory product of the 5-lipoxygenanse pathway in numerous dieases [447]. Recently, Grée et al. [448] devised a simple, efficient synthesis of the enantiomerically pure polyene alcohol (260) which is a key intermediate in preparation of (261) and its 14,15-didehydro derivative.

**Scheme 44:**
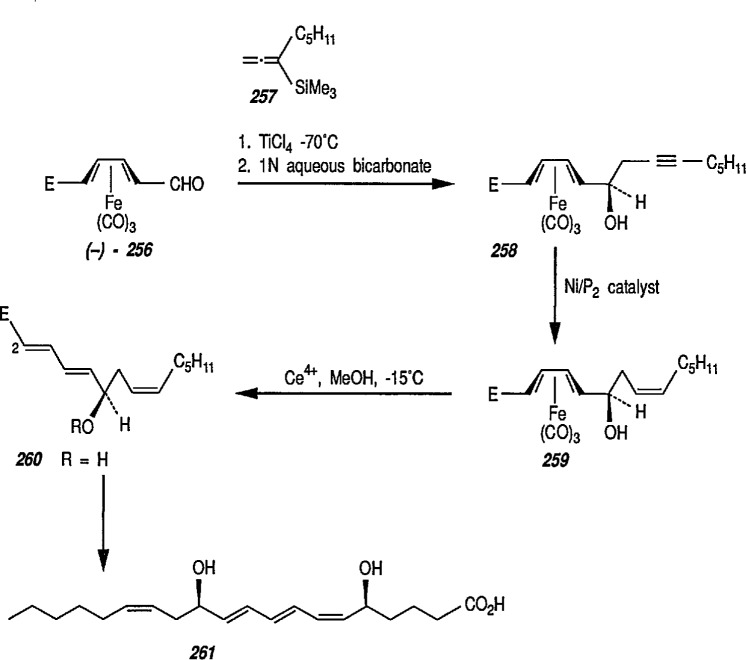


The new approach which starts from the readily accessible chiral complex(−)-256 [449], illustrates some of the key advantages in the use of butadiene tricarbonyl-iron complexes in organic synthesis. The reaction of (−)-256 (of known 1R, 4S absolute configuration [449] with allenyl silane (257) (2 equiv.) in the presence of TiCl_4_ (5 equiv.) at −70°C gives the homoproparyl alcohol (258) (65% yield after chromatography). The reaction is stereospecific (*de* ⩾ 98%) and leads only to the Ψ-endo derivative, with the R configuration at the secondary alcoliol function. Fe(CO)_3_ acts here as an efficient diene-protecting group. Semireduction of (258) proceeds smoothly using Ni/Pt catalyst to give a quantitative yield of (259). Decomplexation (Ce^4+^, MeOH, −15°C) occurs without racemization, leading to the desired key intermediate the polyene alcohol (260). The optical purity of (260) is ascertained by H-NMR using Eu(tfc) shift reagent. The polyene (260) is then transformed to either 14,15-didehydro-LTB_4_ or Leucotriene B_4_ (LTB_4_) (including important labeled LTB_4_) according to the published procedure [450] (Scheme 44). Synthesis of (−)-verbenalol and (−)-epiverbenal via a common chiral iron complex, e.g., 256 has recently been described by the same group [451].

In another recent communication [452] the Grées group reported a new stereoselective synthesis of the *erythro* and *threo* carbonates 263, which are key intermediates for the preparation of (5,6)-DIHETES and Lipoxin A_4_ (polyhydroxylated metabolities of the arachidonic acid cascade with their potent biological properties). Starting from the butadiene-tricarbonyl-iron complex 262, which has been resolved, the multi-step procedure includes a key step of highly diastereoselective osmylation of double bonds vicinal to the organometallic complex (e.g., the dienyl-Fe(CO)_3_ moiety). Interestingly, in each case, the addition of OSO_4_ onto the free double bond occurs *anti* to the Fe(CO)_3_ moiety. The stereochemistry of carbonates has been established by x-ray crystallography. Being very bulky, the Fe(CO)_3_ group in 1,3-dienes is also a good stereodirecting group; the diastereoselectivity of the reactions depending essentially upon the structure and the conformational properties of the starting complexes. The potentialities of the butadiene tricarbonyl iron complexes in synthesis with extension to chiral synthesis appear to be noteworthy, and the topic is of current interest [30,31,227,228].

**Figure:**
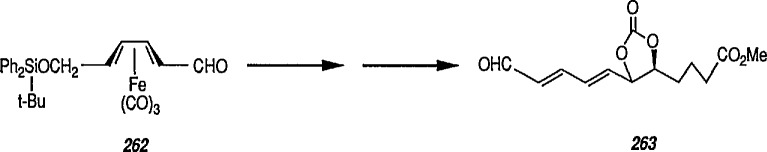


### 7.7 Synthesis of Natural Products and Drugs via Chiral Organoiron Enolates. Use of Enolate from tlie Davies’ Cliiral Auxiliary [(η^2^-C_5_H_5_)Fe(CO)(PPH_3_)]

Chiral enolates are an important class of reagents [304,356,453]. Excellent diastereoselectivity has been achieved with chiral organic auxiliaries [454], and transition-metal centers can also act as chiral adjuvants in enolate reactions. Stereoselective reactions of iron enolate species derived from [(η^5^-C_5_H_5_)(CO)Fe(CO)(PPh_3_)CH_2_R] have been studied extensively [60,62,64,317, 319,341]. Usually the iron chiral auxiliary [(η^5^-C_5_H_5_)Fe(CO)(PPh_3_)] exerts powerful stereochemical control over the reactions of the attached acyl ligand [61,63]. By treating the acetyl complex with butyllithium we can efficiently generate the corresponding enolate, which may be trapped by a variety of allyl halides, and further treatment with butyllithium generates the corresponding *E*-enolates completely stereoselectively. This methodology was successfully applied to the synthesis of enantiomerically pure β-lactams [327,328,347].

**Scheme 45:**
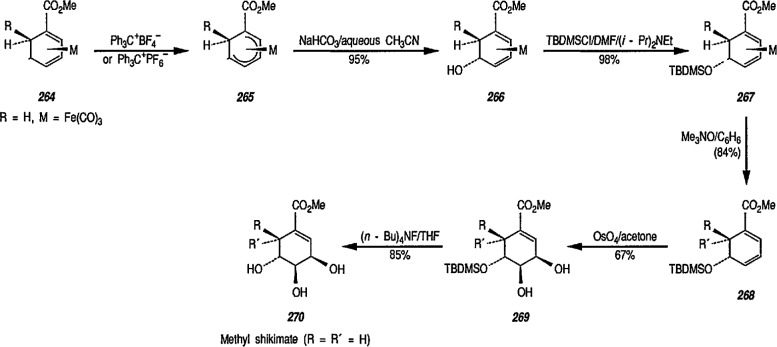


**Scheme 46:**
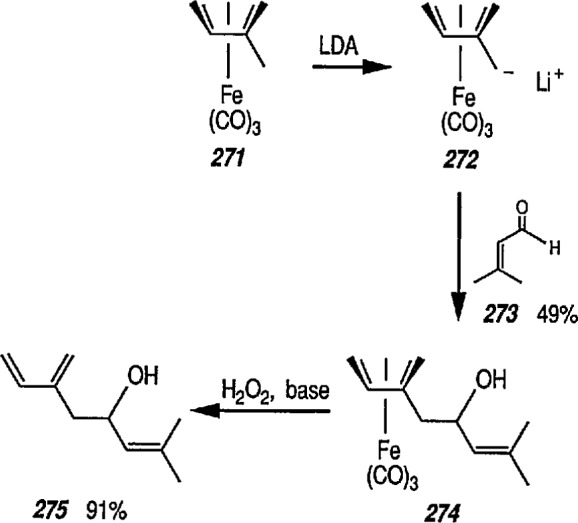


**Scheme 47:**
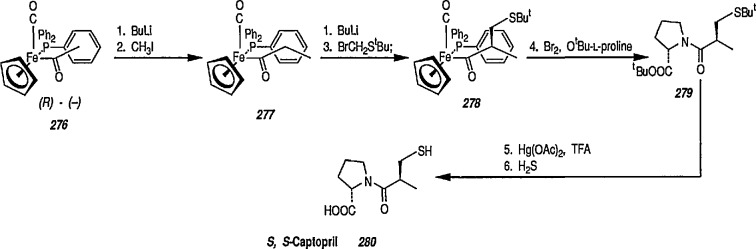


**Scheme 48:**
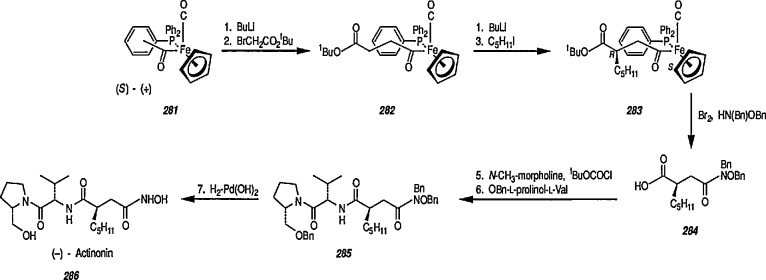


The use of chiral organometallic nucleophiles as intermediates in the synthesis of natural products or drugs demonstrated the practical potential of these synthetic methods [60,62]. The potential of the iron chiral enolate for asymmetric synthesis is illustrated by asymmetric syntheses of (−)-shikimic acid (Scheme 45), of the bark beetle sex pheromone (Scheme 46), of the antihypertensive drug (−)-captropril (Scheme 47), and of the potent collagenase inhibitor (−)-actinonin (Scheme 48).

#### 7.7.1 Synthesis of (−)-Shikimic Acid

The stereochemical synthesis exercised by a complexed transition-metal atom can often mimic the control exercised by enzymes but with a wider range of reaction mechanisms of substrates [445,456]. As compared to the classical synthesis approach, a new concept of superimposed lateral control of reactivity, stereochemistry and structure, by attachment of complexed metal ions to alkene and diene systems, has been discussed at length [457].

A facile synthesis of (−)-shikimic acid (as its methyl ester) using Fe(CO)_3_ as a stereocontrol group has recently been described by Brich et al. [124]. The key intermediate in this enantiospecific synthesis was the resolved 1,3-cyclohexadieneiron complex 264 (R = H) obtained from benzoic acid via 1,4-dihydrobenzoic acid. The derived optically pure cation 265 (R = H) (obtained by a hydride abstraction from 264, compare sec. 3.1) has been shown to react with nucleophiles solely at the 5-oxo position [458]. Reaction of (+)- 265(R = H) in acetonitrile solution with aqueous sodium hydrogen carbonate yielded the alcohol complex (+)-266(R = H). Protection of its OH by reaction with *tert*-butyldimethylsilyl chloride (TBDMSCl) and diisopropylethylamine (to give 267) followed by decomplexation with anhydrous (CH_3_)_3_NO, provided the free diene (+)-268(R = R′= H) in 78% yield from (+)-265(R = H). The conversion of this diene into (−)- methyl shikimate (270, R = R′ = H) was achieved in 67% yield via *cis*-diol 269 (R = R = H) using osmium tetraoxide, followed by fluoride ion to remove the silyl-protected group. The product was identical in properties with (−)-methyl shikimate (Scheme 45). Applying a similar procedure deuterium was incorporated enantiospecifically to give (6R)- or (6S)-methyl 6-dideuterioshikimate. Shikimic acid is a very important biosynthetic intermediate, particularly in biological aromatization reactions.

#### 7.7.2 Synthesis of the Bark Beetle Sex Pheromone

(η^4^-Isoprene)Fe(CO)_3_ (271) can be deprotonated at low temperature to give the isoprene anion equivalent (272), an attractive synthon for isopropenoid natural product synthesis; Semmelhack and Fewkes [459] have then reacted (272) with series of electrophiles. Reaction of (272) with the aldehyde (273) (to give 274), followed by decomplexation with hydrogen peroxide gave the bark beetle sex pheromone 275 in 91% yield (Scheme 46).

#### 7.7.3 Synthesis of (−)-Captropril

The next asymmetric syntheses involve a novel application of enolates from the Davies’ iron chiral auxiliary [(η^5^-C_5_H_5_)Fe(CO)(PPh_3_)]. The steps in the asymmetric synthesis of (−)-captropril (280) involve deprotonation and methylation of the R-(−)-acyl complex (276) to give the propanoyl derivative (277). Further alkylation with bromomethyl *tert*-butyl thioether stereoselectivity generated the new chiral center with the required absolute configuration yielding (278). Oxidative decomplexation with bromine in the presence of the *tert*-butyl ester of L-proline produced double protected (−)-captropril (279). Deprotection with trifluoroacetic acid and mercuric acetate then gave (−)-captropril (280) in 59% overall yield [60,460] (Scheme 47).

#### 7.7.4 Synthesis of (−)-Actinonin

The seven-step asymmetric synthesis of (−)-actinonin (286) (Scheme 48) also demonstrates the use of the iron chiral auxiliary to provide a differentially protected chiral succinate enolate equivalent, as compared to a chiral propionate equivalent in the former case. Alkylation of the S-(+)-acetyl complex (281) with *tert*-butyl bromoacetate generated the corresponding succionyl complex (282). Deprotonation of (282) occurred α to the ester acyl function rather than α to the acyl function, to generate, on addition of pentyl iodide, the corresponding β-pentyl succionyl derivative (e.g., 283) regio- and stereoselectivity with the required absolute configuration at the new chiral center. Decomplexation with bromine in the presence of N,O-dibenzylhydroxyl-amine occurred with concomitant deprotection of the acid function, to give (284). Standard coupling procedures involving a chiral amine derived from prolinal and valine converted the free acid to tribenzyl actinonin (285). Deben2ylation under hydrogenation conditions then yielded (−)-actinonin (286) in 41% overall yield [60,461]. (−)-Actinonin exhibits antibotic activity, anticancer activity and anticollagenase activity. The latter property makes it a potential candidate for the treatment and prevention of arthritis.

#### 7.7.5 Synthesis of (R,S)-1-Hydroxypyrrolidin-3-One

The alternative procedure for the synthesis of alkaloids via cyclohexadienyliron complexes [110,374] (sec. 7.1) has recently been reported by Beckett and Davies [462]; this procedure requires an aluminum enolate derived from the chiral iron acetyl [(η^5^-C_5_H_5_)Fe(CO)(PPh_3_)(Ac)](287). Here in the aldol reaction between the aluminum enolate derived from (287) and BOC-*L*-prolinal (S)-(288), the iron chirality overpowers the latent stereoselectivity inherent in the BOC-L-prolinal to allow, after deprotection and decomplexation, the synthesis of (1R,8S)-1-hydroxypyrrolizidin-3- one (291) (an important class of the plant-derived pyrrolizidine alkaloids).

**Scheme 49:**
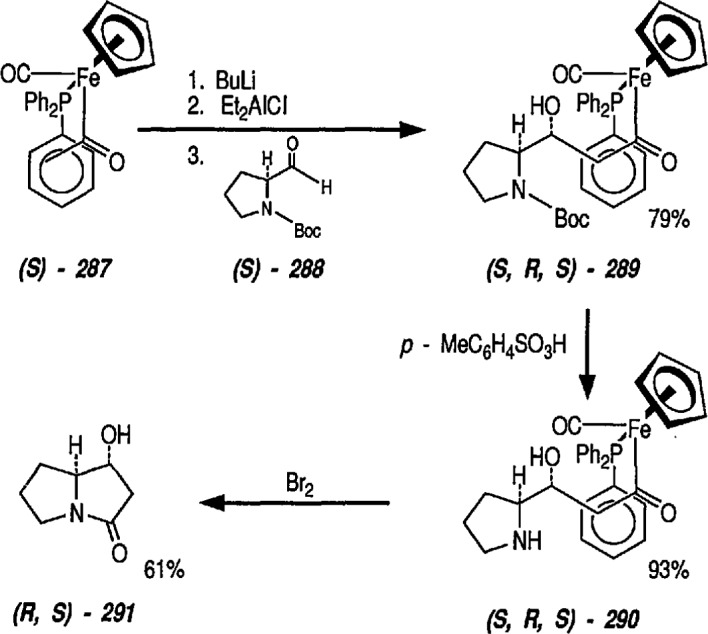


Thus deprotonation of (S)-(287) gave the corresponding Hthium enolate. Transmetallation with diethylaluminum chloride and addition of (S)-(288) gave (S,R,S)-(289) as a single diastereoisomer (> 300:1). Deprotection with toluene-*p*-sulfonic acid gave the corresponding (S,R,S)-β-hydroxy-γ-amino complex (290) which on oxidative decomplexation yielded (1R,8S)-(291) in 61% yield (Scheme 49). The procedure was used for the synthesis of (S,S)-diastereoisomer starting from (R)-(287).

The future promises exciting possibilities for the use of transition metal based chiral auxiliaries for asymmetric synthesis. The ability of chiral auxiliary based reagents to discriminate between the two enantiomers of a racemic substrate allows the selective transformation of only one enantiomer of the substrate to product, leaving the other unaffected. This type of kinetic resolution [60] results in the conversion of racemic compounds into homochiral materials and represents one of the most promising topics for future research into the preparation of homochiral compounds.

### 7.8 Asymmetric Synthesis with Chiral Ferrocenylamine Ligands: The Importance of Central Chirality

The development of synthesis methodology for the diastereo- and enanthioselective formation of C–C bonds derived through the use of catalytic quantities of chiral transition-metal catalysts is today a topic of fundamental importance [5,41,279,283,463–465].

In 1986, Ito and Hayashi reported an elegant synthesis of oxazolines utilizing a gold(I)-catalyzed aldol reaction in the presence of chiral ferrocenylamine ligands that possess both planar and central chirality [466].

**Scheme 50:**
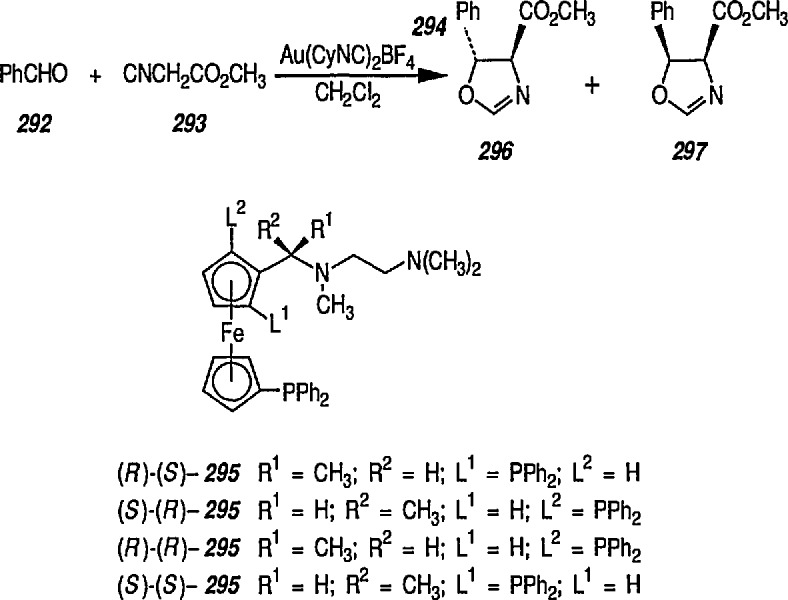


For example, the reaction of benzaldehyde (292) with methyl-α-isocyanoacetate (293) catalyzed by bis(cyclohexylisocyanide) gold(I) tetrafluoroborate (e.g., 294) in the presence of the chiral ferrocenylamine ligand(R)-(S)-295 give a mixture of *trans*- and *cis*-oxazolines 296 and 297, respectively. The trans isomer 296 was the dominant isomer formed in 91% enantiomeric excess (*ee*) [467] (Scheme 50).

Recently, Pastor and Togni [468] examined the effect of varying ligand chirality in the ferrocenylamine side chain, e.g., 295 upon product enantio- and diastereoselectivity in a model reaction of 292 with 293. The results of this study indicate that steric interactions due to the central chirality of the stereogenic carbon atom in the ferrocenylamine side chain play a more important role then previously supposed. Furthermore, the results of this study strongly suggest that planar chirality and central chirality may act in either a cooperative or noncooperative sense. This constitutes the first example in a chiral transition-metal ligand, e.g., ferrocyenylamine (S)-(S)-295 containing both planar and central chirality of internal cooperativity of chirality in the control of product diastereo- and enantioselectivity.

#### 7.8.1 Synthesis of (+)-Corynoline

**Scheme 51:**
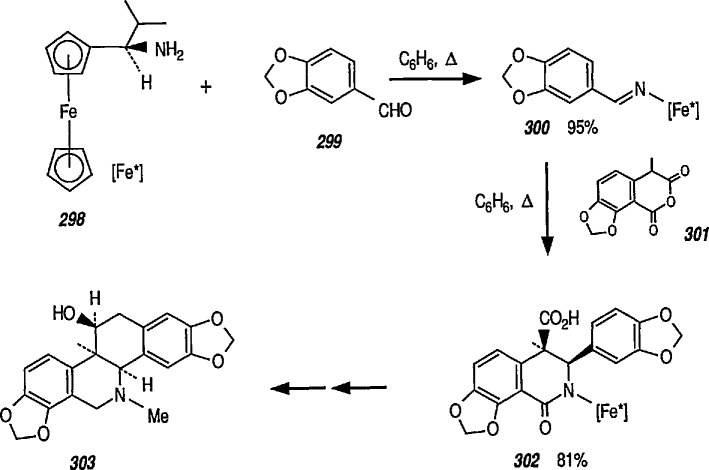


A chiral ferrocenylamine ligand [353] played a key role in another recent chiral application [469]. The key step in the asymmetric synthesis of (+)-corynoline (303) involved the condensation of the chiral 1-ferrocenyl-2-methyl-propylamine Schiff base (300) [obtained by the reaction of the ferrocenylamine (298) (Fe*) with the aldehyde (299)] with racemic homophthalic anhydride (301) to afford the required chiral intermediate (302) in 81% yield; the latter was then transformed into natural (+)-corynoline (303) (Scheme 51). Here the chiral auxiliary influences both the relative and the absolute configuration of two asymmetric centers [469].

A new chiral ferrocenylphosphine ligand with C_2_ symmetry has recently been prepared and used for palladium-catalyzed asymmetric cross-coupling reactions [470].

## 8. Miscellaneous Recent Results

Some highlights of recent applications of organoiron complexes in synthesis are described next.

### 8.1 Organoiron Complexes in Carbon-Carbon Bond-Formation Reactions

#### 8.1.1 Enantioselectivity in Bu_4_N[Fe(CO)_3_NO]–Catalyzed Nucleophilic Substitution of Optically Active Allylic Carbonates with Malonate

Among the various carbon-carbon-bond-forming reactions promoted or catalyzed by transition metals, allylic alkylation has been one of the most aggressively sought after. Accordingly, in recent years, extensive studies have been devoted to the regio- and stereochemistry of these allylic alkylation reactions catalyzed by different metal complexes, including iron [100,471]. Encouraged by good regioselectivity, geometric selectivity, and diastereoselectivity exhibited in Bu_4_N[Fe(CO)_3_NO] (304) – catalyzed allylic alkylation [100], the study has now been extended on the enantioselectivity of this iron-catalyzed reaction. Here the reaction of several optically active allylic carbonates with a nucleophile sodium dimethyl malonate catalyzed by Bu_4_N[Fe(CO)_3_NO] has been examined [472].

Thus, the allylic carbonate (R,E)-305 ${\left[\alpha \right]}_{D}^{25}+89.2$ (C, 1.80, chloroform, 73% *ee*) was allowed to react with 2 equiv of sodium dimethylmalonate in refluxing THF in the presence of 25 mol% of (304) under CO atmosphere for 12 h. Workup followed by flash chromatography on silica gel gave 78% yield off the alkylated products consisting of [1-(E)-styrylethyl] malonate (306) and its regioisomer, dimethyl[1-phenyl-2(E)-butenyl]malonate (307), in a ratio of 93:7. This method was also applicable in reactions of optically active allylic carbonates with a terminal double bond.

**Figure:**
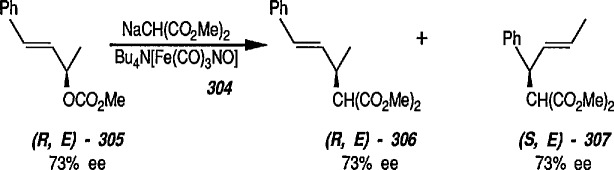


#### 8.1.2 Regioselective Addition of Nucleophiles to Cationic Diiron μ-Vinylcarbyne Complexes

Recently Casey et al. [473] described a new procedure for the synthesis of functionalized diiron μ-alkenylidene complexes. When *p*-tolyllithium was added to a purple suspension of the *p*-tolyl substituted vinylcarbene complex (308) in THF at −78 °C, (308) gradually dissolved to form a red solution from which the alkenylidiene complex (309) was isolated in 49% yield as a red-orange powder. The complex (309) was formed by the regioselective addition of a *p*-tolyl group to the remote vinyl carbon of (308); its structure was established by spectroscopy.

**Figure:**
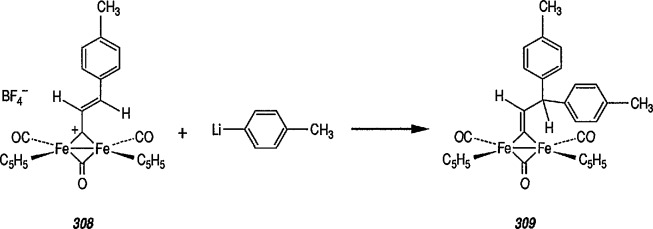


The diiron methylidene complex ${\left[\left(\mu {-C}_{5}H_{5}\right)\left(\text{CO}\right)\text{Fe}\right]}_{2}\left(\mu -\text{CO}\right){\left(\eta -\text{CH}\right)}^{+}{\text{PF}}_{6}^{-}$ forms 1:1 adducts with a variety of heteroatom and carbon nucleophiles such as (CH_3_)_3_N; it also reacts with carbon monoxide to give 1:1 adduct. This adduct then reacted with nucleophiles at the acylium carbon to give a series of useful derivatives such as aldehydes, carboxylic acids, amides etc. [474].

A new procedure for regiospecific lithiation of permethylated (η^6^-tricarbonylchromium) phenylacetyl (tetracarbonyl) iron anion has recently been described [475]; this is a versatile method for the α,α′-ortho-bifunctionalization of hexamethylbenzene.

#### 8.1.3 Applications of Ferrocene Derivatives

A recent survey of ferrocene chemistry has appeared [476]; additions to techniques developed for functional-group interconversion involve some ferrocene derivatives.

**Scheme 52:**
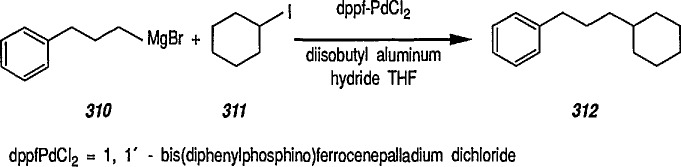


It has been presumed that catalytically formed alkyl-palladium species would undergo destructive beta-hydride elimination too rapidly for use in organic synthesis. However, a recent report [477] shows that 1,1′-bis(diphenyl-phosphine)ferrocene (dppf) ligand effectively suppresses beta-hydride elimination. For example, exposure of a primary alkyl Grignard reagent, such as 310, to a relatively sluggish-acting iodide, such as 311, in the presence of 5 mol percent of the dppf·PdCl_2_ catalyst (67 °C for 16 h) gives the coupling product 312 in 91% yield (Scheme 52). Here, the dppf·PdCl_2_ catalyst effects the cross-coupling of alkyl iodides with alkyl Grignard reagents; and this is a useful method for selective carbon-carbon bond-formation.

Some isocyclopentadiene derivatives of ferrocene have been used as models for study of the mechanism of the π-facial, selective course of Diels-Alder cycloadditions [478]. Synthetic applications of mixed-valence ferrocenes have recently been explored [479].

#### 8.1.4 Application of Iron Heterocyclics

**Scheme 53:**
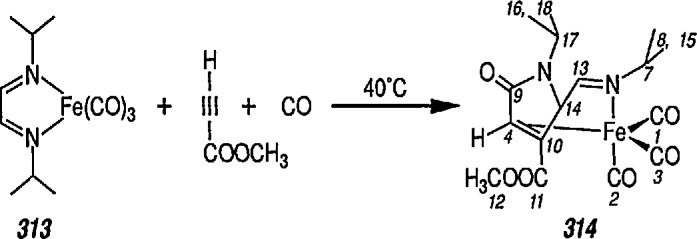


Today, the design of new catalytic transition-metal-mediated carbon-carbon bond-constructions that are of utility in the course of organic synthesis remains an important challenge; this is supported by a variety of new synthetic uses of organoiron complexes. For example, a new 2,2′-bipyridine-Fe(O)-diene complex has been applied for the regio- and chemoselective cross-coupling reaction with various alkenes [480]. In the heterocyclic series, a new reaction of diazadiene-tricarbonyliron complex 313 with the electron-deficient alkynes (e.g., methyl propynoate) in the presence of CO as an additional ligand leads to the formation of (1,5-dihydropyrrol-2-one)-tricarbonyliron complex 314; a bicyclic reaction-intermediate has been isolated and characterized [481] (see Scheme 53).

#### 8.1.5 Use of Optically Active Iron-Alkene Complexes

**Scheme 54:**
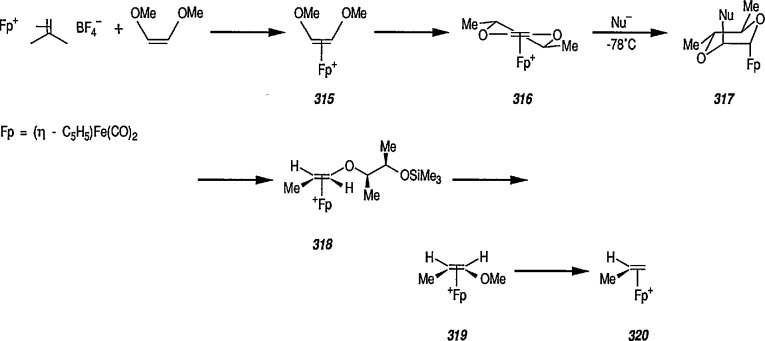


Optically active metal-alkene complexes in which the alkene itself is a center of asymmetry provide unique substrates for asymmetric carbon-carbon bond-formation [14]. Recently, Rosenblum et al. [37–40,482] reported the preparation of the optically active iron complex 315, which serves both as a unique platform for asymmetric C–C bond-formation and for the preparation of optically active alkyl vinyl ether-iron complexes in which the exclusive locus of asymmetry is the alkene center. Complex 316 adds nucleophiles of a broad range of basicities (e.g., NaBH_3_CN, PhMgBr, PhCH_2_SH, etc.) yielding the single, optically active adduct 317. The high regioselectivity observed in the reaction of 316 with nucleophiles may be due to stereoelectronic control of addition. Complex 316 can be prepared from 315 by exchange etherification with ethylene glycol (CH_2_Cl_2_, 0 °C, 0.25 h; Et_2_O, 94%). Complex 317 is then quantitatively converted into (R)-318 (E_480_= +1.34 M^−1^ cm^−1^, 0°), on treatment with Me_3_Si triflate and CH_2_Cl_2_-Et_2_O at −78°C for 10 min. This may be further transformed by reduction (NaBH_4_, −78 °C) and treatment with HBF_4_·Et_2_O (−78 °C) into the first iron-alkene complexes 319 and 320 which owe their optical activity entirely to asymmetry of alkene complexation (see Scheme 54).

Recently, a promising dynamic resolution of vinyl ether-iron complexes has been reported by the same group [40]. Thus, optically active vinyl ether-iron complexes (a prototype of 320) can be readily generated from the racemic ethyl vinyl ether-iron complexes by alkoxy exchange with optically active alcohols. The best results are achieved with (−) or (+)-menthol yielding diastereomeric complexes with ratio 4.0:1 or 3.6:1 respectively.

A series of new, chiral, iron complexes of the type [η^5^-C_5_H_4_CH(Ph)Fe(CO)-(L)X], where (L=phosphine, phosphite, X=acyl, alkyl, halide, hydride), have been synthesized and characterized [48,53].

(η^4^-tropone)Fe(CO)_3_ and (η^4^-isoprene)Fe(CO)_3_ form separable diastereoisomers on replacement of CO by (+)-(neomenthyl)PPh_2_. In the tropone complex, diastereoisomer interconversion occurs by a 1,3-metal shift. The absolute configuration of this isoprene complex has been determined crystallographically [483].

It has recently been shown [585] that (η^4^-1-chloro-2,5-diphenylsilacyclopentadiene)tricarbonyliron complexes undergo nucleophilic displacement at silicon with complete retention of configuration at both the *exo* and the *endo* positions. The substitution is faster at the *exo* than at the *endo* position. The observations were discussed in terms of electronic factors.

### 8.2 New Reactions of Bridged Organoiron Complexes

**Scheme 55:**
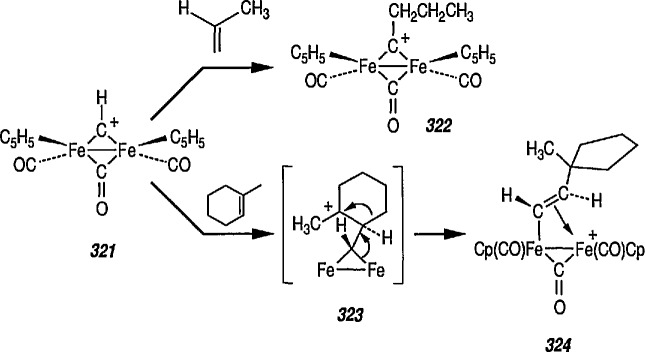


The reaction of the cationic diiron bridging methylidene complex 321 (which is very electrophilic) with activated alkenes has recently been examined by Casey et al. [47,485,486]. The bridging methylidene complex alkenes, 321 adds its C–H bond across the carbon-carbon double bond, to afford μ-alkylidene complexes. For some alkenes, such as 1-methylcyclohexene, *trans*-stilbene, and 1,1-diphenylethylene, which are more sterically crowded and are capable of forming stabilized carbocation intermediates, the formation of bridging alkenyl complexes has been observed, conversions 321 → 322; 321 → 323 → 324 (Scheme 55) (Compare also sec. 8.1.2).

### 8.3 Enzymatic Resolution of Chiral Butadiene Tricarbonyliron Complexes by Pig Liver Esterase

A few 1,3-diene tricarbonyliron complexes have been resolved, almost all by classical means (formation of diastereoisomeric derivatives) [449,457,487–490]. The first example of an enzymatic resolution of an organometallic complex has recently been reported [491]. Thus, the racemic ester (2-ethoxycarbonyl-buta-1,3-dienetricarbonyliron) (325) was hydrolyzed enantioselectively to the corresponding crystalline acid (326) by pig liver esterase (PLE); the reaction reached completion in about 40 h at pH 7. The optical purity of the acid (326) was found to be of 85% *ee* which was raised to > 98% by one recrystallization. The ability of hydrolytic enzymes to recognize chirality of the type exhibited by this complex warrants further exploration of this useful synthetic procedure.

**Figure:**
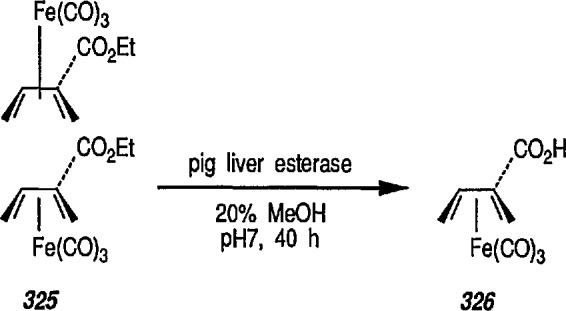


### 8.4 The Stereoselective Reaction of Nonbornyl Aldehydes with Diiron Nonacarbonyl [Fe(CO)_9_]

The reported reactions of aldehydes with iron carbonyl reagents have been limited to α,β-unsaturated systems in which stable π-complexes are formed [492,493]. As recently found [494] the norbornyl aldehydes do not form stable complexes in the presence of Fe(CO)_9_, instead the reaction proceeds stereoselectively to “geminal-faced” esters or alcohols.

Thus, in the presence of Fe(CO)_9_ in refluxing hexane or tetrahydrofuran (THF), norbornane-2-carboxaldehyde (327) (R = H; R = CH_3_) was converted to the *endo, endo* congener (90% isomeric purity) of norbornane-2-ylmethyl norbornane-2-carboxylate (328) (R = H; R = CH_3_) in 54% and 71% yields, respectively, after 48 h. In addition, a minor amount (4–6%) of the reduction product, *endo*-2-(hydroxymethyl)norbornane (329) (R = H) was generated as well, which possessed an isomeric purity of 85%. Although yield enrichments were observed for esters (328) (R = H; R = CH_3_) with a change in solvent, alcohol formation remained approximately the same. These results emphasized the importance of solvent characteristics as a parameter for ester synthesis. It is well known [495] that THF stabilizes iron carbonyl through complexation, and this evidently contributed to the higher ester yields in that medium.

**Figure:**
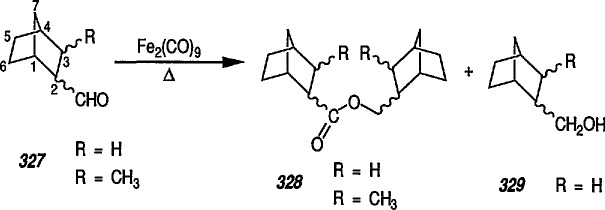


### 8.5 Ring-Opening Reactions

#### 8.5.1 Novel Ring-Opening Reaction of Norbornadiene (Tricarbonyl)Iron

**Scheme 56:**
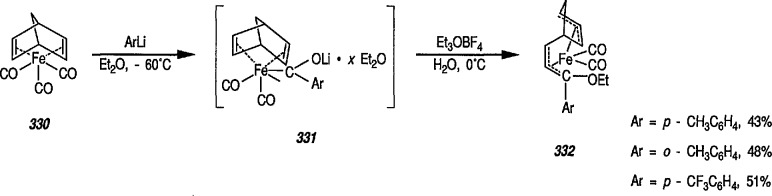


This study describes an activation of nonconjugated cyclic polyene ligands like norbornadiene by iron and reports a novel ring-opening reaction of norbornadiene (tricarbonyl) iron (330) under mild conditions [496]. Thus reaction of (330) with aryl-lithium reagents (ArLi; Ar=*p*,*o*-CH_3_C_6_H_4_; *p* =CF_3_C_6_H_4_) at low temperature (ether, −60°C), followed by alkylation of the intermediate acylmetallates (331) with Et_3_OBF_4_ in aqueous solution at 0 °C, leads to cleavage of the norbornadiene ring, to give novel ring-opened diallyl complexes (332) (Ar=*p*,*o*-CH_3_C_6_H_4_; *p* = CF_3_C_6_H_4_) in moderate yields. This reaction implies that the σ-bonds of cyclic polyene ligand in the complex (330) are activated by the iron atom, resulting in the breaking of a C–C σ-bond and formation of a new one. All structures have been characterized by x-ray analysis (Scheme 56).

#### 8.5.2 Step-Wise Cleavage of 1,1-Bis(Diphenylphosphino)Ethylene Iron Complex

**Scheme 57:**
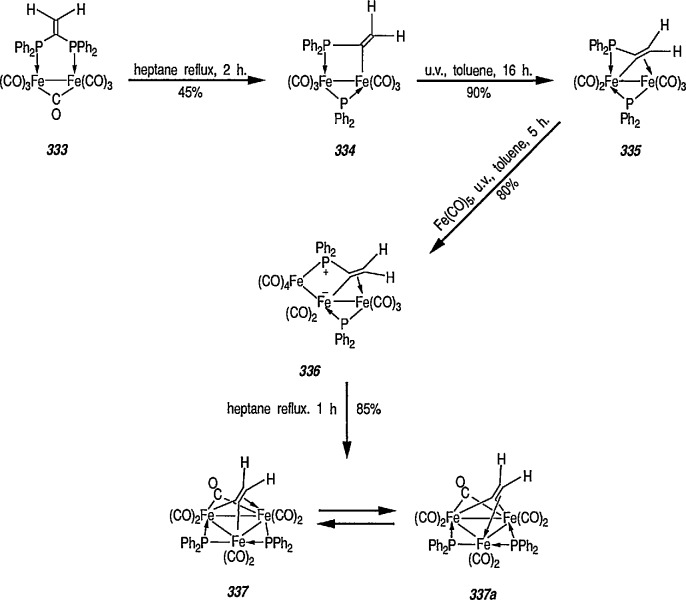


In another recent example, a step-wise cleavage of 1,1-bis(diphenylphosphino)ethylene (333) at di- and tri-iron centers under thermal and photochemical conditions has been reported [497]. In refluxing heptane the complex (333) loses a CO ligand to generate the phospha-allene complex (334), containing an unusual four-membered ring with an exocyclic double bond. The complex (334) is then brought into coordination via photochemically induced loss of a second CO, yielding the α-phosphinovinyl complex (335). Treatment of (335) with Fe(CO)_5_ under uv irradiation results in the insertion of an Fe(CO)_4_ fragment into Fe(1)-P(2) bond to afford (336). In refluxing heptane two molecules of CO are lost from (336) resulting in the formation of the μ_3_-vinylidene complex (3371 ⇆ 337a). Thus, the sequence 333 → 334 → 335 → 336 comprises the step-wise fragmentation of (333) at a di-iron center, for the first P–CCH_2_ bond cleavage and at tri-iron center, for the second. All pertinent structures have been established by x-ray diffraction analysis (Scheme 57).

### 8.6 Reaction of Organosulfur Ligands in Bridged Ironcarbonyls

**Scheme 58:**
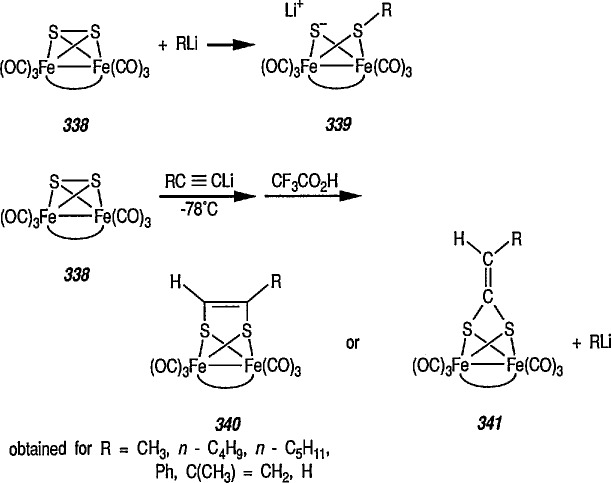


As earlier found by Seyferth et al. [498], the S–S bond of (η-dithio)-bis(tricarbonyl)iron 338 is readily cleaved by organometallic nucleophiles, to give the intermediate lithium thiolate 339 that could be protonated, alkylated, and acylated. However, as recently reported [59], the reaction of 338 with alkynylithium reagent in THF at −78 °C, followed by protonation (CF_3_CO_2_H) gave “closed” 1,2- or 1,1′-dithiolene products, e.g., 340 and 341 (Scheme 58). Formation of the sulfur-bridged products generally depends very much on whether the reaction mixtures were protonated, or not,

A selected recent work on organosulfur-iron complexes includes reactions of lithium bis(η-phenylphosphido)-bis(tricarbonyliron) with organic halides [499], synthesis and reactivity of (η-σ-π-acetylide)(η-alkane- and η-arene-thiolate) bis(tricarbonyliron) complexes [500]; also synthesis of *cis-trans*-Fe(CyNC)_4_(SPh)_2_ [501], chelation of iron (II)dithiocarbamates [502], thiolate, thioether, and thiol derivatives of iron (0) carbonyls [503], reaction of {CpFe(CO)MeCN) = C[SMe_2_]} PF_6_ with NaSMe [504], cyclodextrin sandwiched Fe_4_S_4_ cluster [505], and iron-sulfur proteins containing Fe_2_S_2_ clusters [506,507].

### 8.7 Photoinduced Reactions Involving Ironcarbonyl Complexes

Photochemical reactions of transition-metal carbonyl complexes find frequent application in synthesis and catalysis [508,509]. Interesting chemistry and photochemistry has developed on the basis of the tricarbonyliron moiety [510]. This functional group binds numerous alkenic substrates and can activate them toward nucleophilic attack [34,35]. Photosubstitution reactions of the tricarbonyliron fragment are of synthetic utility due to kinetic inertness of the Fe(CO)_3_ group. Pioneering work by Von Gustorf and his group [511] demonstrated efficient CO substitution when Fe(CO)_3_-(1,3-diene) complexes were excited with ultraviolet radiation. A few new examples from the recent literature are reported next.

**Scheme 59:**
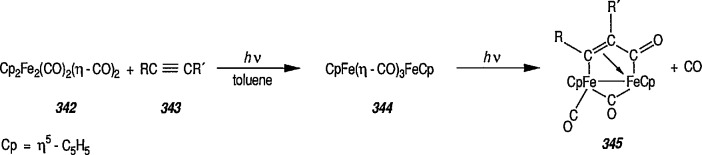


A dinuclear species Cp_2_Fe_2_(CO)_2_(η-CO)_2_ (342, Cp=η^5^-C_5_H_5_) has a rich and diverse photochemistry, as evidenced by the plethora of synthetic and mechanistic studies of it in the literature [508,509,512]. A recent study reports [512] a photochemical addition of alkynes to 342 involving photochemical substitution and insertion reactions and formation of the photoproduct intermediate CpFe(η-CO)_3_FeCp (344) by CO loss. Thus laser flash photolysis of 342 in toluene in the presence of alkyne (e.g., 343) shows the formation of an intermediate (e.g., 344) with a strong absorbance at 515 nm; further reaction of 344 with alkyne yields the insertion product 345 (Scheme 59). The mechanism of this reaction has been explained.

Photochemical substitution reactions of iron trlcarbonyl 1,4-dimethyltetraazadiene (346) and related complexes have also been examined [513]. It has been shown that ligand field excited states generally promote substitution reactions, whereas charge-transfer states are considerably less reactive in this regard. Thus a photosubstitution of CO in tricarbonyliron 1,4-dimethyltetraazadiene (346) (in conversion 346 → 347) proceeds via a dissociative mechanism, in contrast to the corresponding thermal reaction, which is of associative character. Although free tetraazadiene ligands are unknown, the iron carbonyl complex 346 is quite stable. No decomposition of 346 occurred, even after 70 h of refluxing in toluene.

**Figure:**
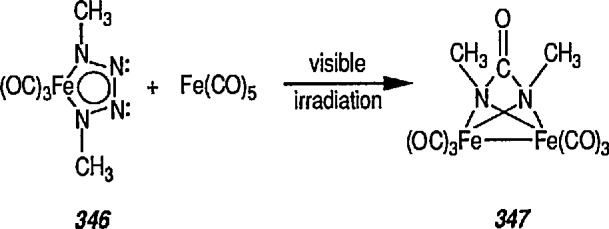


**Scheme 60:**
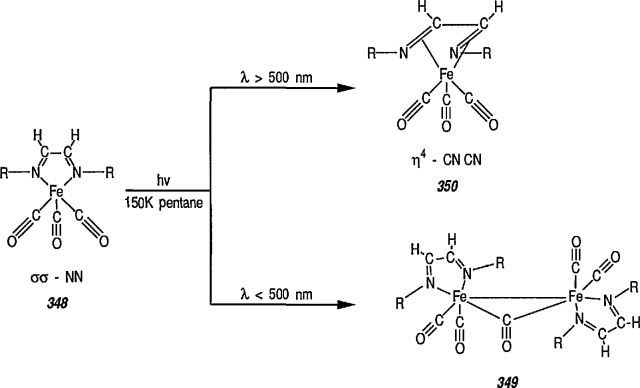


Another recent study showed [514] that photolysis of the organoiron complex 348 at low temperature (in n-pentane at 150 K) can lead to two products depending on the wavelength of irradiation. The main product upon high-energy (λ<500 nm) excitation is a binuclear complex 349 with a bridging CO ligand. Upon irradiation with a longer wavelength (λ>500 nm) the main reaction is a change of coordination from σ,σ-NN to η^4^-CN, C′N′ to give 350 with a retention of the Fe(CO)_3_ moiety (Scheme 60).

Recently Casey and Austin [515] developed a new carbon-carbon bond-forming reaction based on unique photoreaction of the [(η^5^-C_5_H_5_)(CO)Fe]_2_(η-CO)(η-CR) framework. Thus photolysis (366 nm, toluene, 0 °C) of the neutral μ-ethenylidene diiron complex 351 in the presence of ethyl diazoacetate (a trapping agent) produced the μ-allene complex 352 ⇆ 352a in 48% yield; the complex then can be cleaved to free allene. The synthesis of the μ-allene complex 352 required light and was inhibited by carbon monoxide. The use of hydrogen or trialkylsilanes as the trapping agents produces alkenes or vinyl-silanes.

**Figure:**
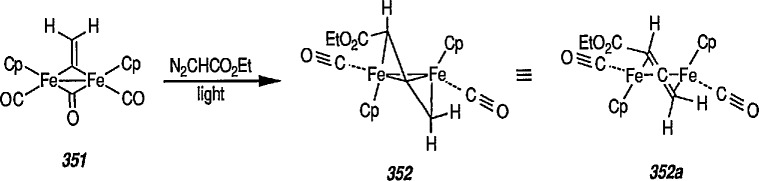


**Scheme 61:**
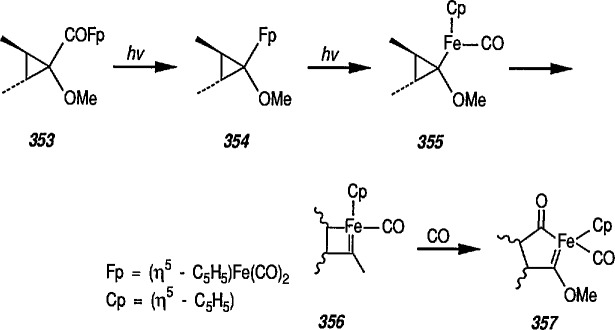


A new, photoinduced, ring expansion, either of cyclopropaneiron [516] or of cyclobutyliron [517,518] σ complexes has recently been described. Thus, photolysis of cyclopropaneiron 353 (benzene solution, N_2_, 450-W Hanovia lamp) gave the rearranged carbene complex 357 via the cyclobutane intermediate 356 (conversions 353 → 357 Scheme 61). The driving force for this rearrangement is a combination of relief of ring strain (357 is less strained than 355) and stabilization of the carbene 357; 357 is stabilized by electron donation from the methoxyl group more than is 355.

Irradiation of 358 (5,6-bismethylene-7-oxabicyclo[2.2.1]hept-2-ene) in methanol in the presence of Fe_2_(CO)_9_ at −20 °C resulted in formation of the cyclopentanone derivative 359 [519]; the latter can be oxidatively demetalated [(CH_3_)_3_NO] to a very interesting, cyclic tetraalkene derivative.

**Figure:**
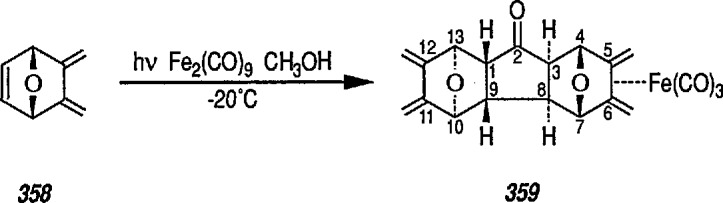


The readily available, chelated bis(silyl)iron complex 360 reacts photochemically with nitriles RCH_2_CN, to give *N*,*N*-bis(silyl)enamines 361 in good yields. Aliphatic and benzylic nitriles give high yields of a Z and *E* mixture of enamines. With dinitriles, the reaction occurs only with the cyano compound, which has an α-hydrogen atom. The procedure constitutes a novel, chemical transformation of nitriles into silylenamines [520].

**Figure:**
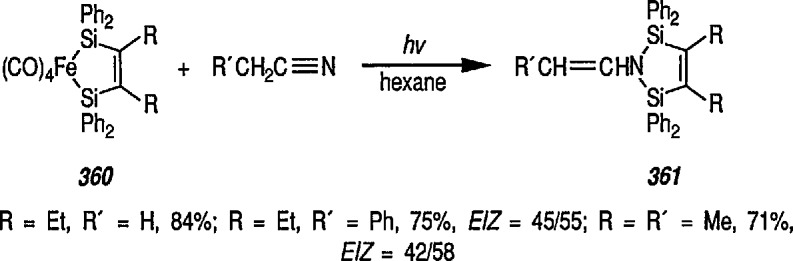


Recently, an interesting, photochemically-induced reduction of nitrosoarenes by Fe(CO)_5_ to give azoxy- and azoarenes has been reported. The mechanism for formation of azoxyarenes apparently involves a ligand exchange to give nitrosoarene-iron complex which on deoxygenation by a CO ligand produces an unsaturated iron-nitrene intermediate. The latter rapidly couples with unreacted nitrosoarene to generate azoxyarene [521] (conversions 362 → 365).

**Figure:**
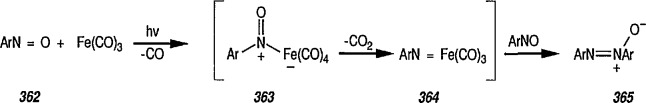


Other reported reactions involve the photoinduced, oxidative addition of an aromatic C–H bond of a distal benzyl group to the Fe center [522], and photoinduced conversion of the (η^5^-C_5_H_5_)Fe(CO)_2_(η^1^-C_5_H_5_) complex into the ferrocene derivative [523]; also the photochemistry of some (η^4^-cyclopentadiene)Fe(CO)_3_ complexes [524], and photochemical insertion of alkynes into the PR bridged clusters, e.g., (η_3_-PR)Fe_3_(CO)_10_ [525] and (η_4_-PR)_2_Fe_4_(CO)_11_ [526] have been reported. The low temperature studies also include photochemical reaction of alkenes with Fe(CO)_5_ [527], photochemical reaction of Fe(CO)_2_(NO)_2_ with 1,3-butadienes [528]; also, photochemistry of [Fe_2_(CO)_7_L](L=2,2′-bipyridine, 1,10′-phenanthroline), causing substitution of CO by nucleophiles [529], and photochemical rearrangement of (η^2^-C_5_Me_5_)Fe(CO)_2_CH_2_SiMe_2_H into (η^5^-C_5_Me_5_)Fe(CO)_2_SiMe_3_ [530,531]. The photopolymerization of heptadeca-2,4-diynylferrocenecarboxylate in the monolayer on a water surface has recently been investigated [532]; the polymerization behavior was found to depend markedly on the molecular packing in the monolayer.

### 8.8 Other Recent Results

#### 8.8.1 Novel Phosphorus-Bridging Iron Carbonyl Complexes

The stereodirection of the phosphine ligand (PPh_3_) in triphenylphosphine-substituted organoiron complexes has been recognized [60,64,192,209, 286–289,293] and importance of the phosphorus atoms in transition metal complexes including organoiron complexes is of current interest [36,41,471,531,533–535]. A series of new iron complexes of phosphinine [537,538], phosphine [539–542] and phosphane [522,543,544] derivatives have been prepared and their reactions studied.

**Scheme 62:**
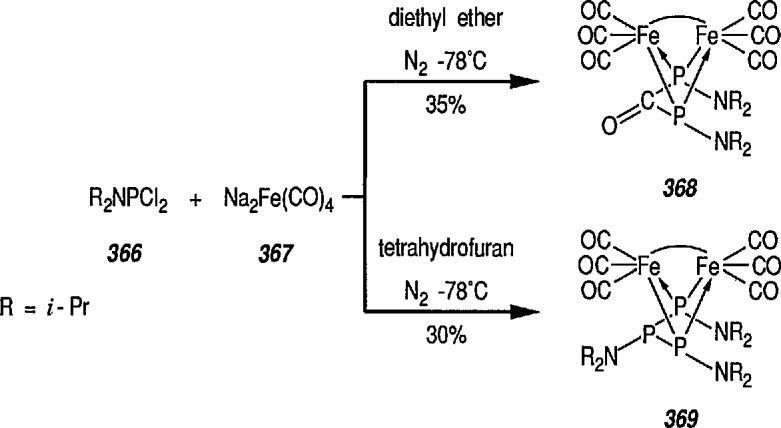


An interest in the chemistry of iron diphosphenes containing phosphorus-phosphorus double bond, e.g., (OC)_4_Fe←RP = PR→Fe(CO)_4_ [42,531,533] has led to numerous studies on their preparation by dehalogenation of RPCI_2_ derivatives with diverse strong reducing agents. Recently, the King group [36] extended this chemistry to reactions of iron carbonyl anions as reducing agents, e.g., Na_2_Fe(CO)_4_ with (dialkylamino)dichlorophosphines, e.g., R_2_NPCl_2_ (R = isopropyl, cyclohexyl) to yield products that may be regarded as iron carbonyl complexes of the diphosphenes. Thus, treatment of R_2_NPCl_2_ (366, R = isopropyl) with Na_2_Fe(CO)_4_ (367) in diethyl ether provided a useful method for the synthesis of the phosphorus-bridging carbonyl derivatives, e.g., the orange complex 368 (R= *i*-Pr) or the orange triphosphine derivative 369 (R= *i*-Pr) in tetrahydrofuran solution. The formation of the complex 369 can be rationalized by the pathway involving the terminal (dialkylamino) phosphinidene complex R_2_NP = Fe(CO)_4_ as a reaction intermediate, which can undergo a redox reaction with $\text{Fe}{\left(\text{CO}\right)}_{4}^{2-}$ in tetrahydrofuran but not in diethyl ether. The structure determined [36] by x-ray analysis of the complex 368 indicates the formation of the Fe-Fe bond bridged by two phosphorus atoms; in structure 369, however, an Fe_2_(CO)_6_ unit (the Fe-Fe bond) is bridged by a triphosphine chain. A reductive elimination of the CO group in 368 (to give 369) was apparently effected in more polar tetrahydrofuran solvent as compared to diethyl ether (Scheme 62).

#### 8.8.2 Addition Reactions of Iron π-Alkynes

**Scheme 63:**
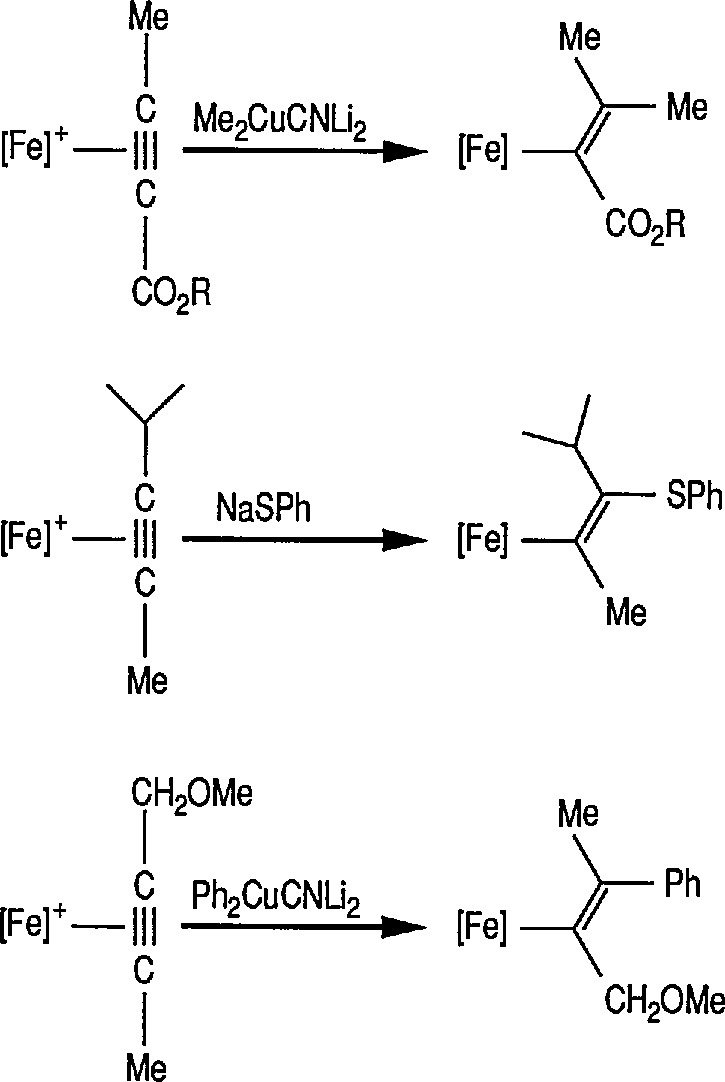


The addition of nucleophiles to unsaturated ligands activated by coordination to transition metals has proven to be useful for the preparation of new organometallic complexes and as a versatile methodology in organic synthesis [14,79]. Despite the considerable interest in this area, addition reactions with η^2^-alkyne complexes leading to (η^1^-alkenyl) metal species had only been briefly studied. Recently, Reger [49] discussed at length nucleophilic addition reactions with cationic iron π-alkyne and related complexes; some typical reactions are shown in Scheme 63.

#### 8.8.3 Some Recent Work on the Chemistry of Ferrocenes and Porphyrins

A new work on iron porphyrins, ferrocenes and related iron compounds has been summarized; among the topics discussed are organic reactions of selected π-complexes [545], stereochemistry of metallocenes [546] and the recent chemistry of ferrocene compounds [547,548]. Other topics of recent interest are: bridged ferrocenes [549], reaction of the isodicyclopentadiene anion with Fe(II)(acac)_2_(Py)_2_ (to yield ferrocene derivatives) [550], preparation of iron-polyarene sandwich compounds [551], and crystal structure of [2]metacyclo[2](1,1′) ferrocenophane [552].

The chemistry of porphyrins, including iron porphyrins, has been discussed in a comprehensive treatise [553] and in recent reviews [554,555]. Recent literature on porphyrins is abundant covering many aspects of biochemistry. Particularly, the reactions catalyzed by iron porphyrin complexes have attracted attention in relevance to the activation of molecular oxygen and oxygen atom transfer to organic substrates, which are processes dependent on cytochrome P-450 in biological systems [556–559]. A recent interest in porphyrins includes iron(III) porphyrins [560], iron AT-substituted porphyrins [561], novel ferrocene bis-porphyrins [562], sterically encumbered iron(II) porphyrins [563], regio- and stereoselective reduction of N(21), N(22)-bridged porphyrins [564], electroreductive alkylation of iron porphyrins [565] and synthesis of (α,β,γ,σ-tetraphenylporphinato)iron(II) [566], and non-heme iron enzymes and proteins [567,568].

#### 8.8.4 Some Recent Pertinent References

Some important recent reviews include: organoiron chemistry [569,570], transition metals in organic synthesis [571,572], transition metals in organic synthesis: hydroformylation, reduction and oxidation [573], and the use of iron (in addition to other transition metals) complexes as catalysts for alkene epoxidation [574].

Recent methodology on chiral separation by high-pressure liquid chromatography has been surveyed [575].

An alkyne oligomerization in the presence of Fe(CO)_5_ (and other metal carbonyls) has been discussed [576].

The transition-metal-mediated cycloaddition reactions of alkynes in organic syntheses have recently been reviewed [577].

The coordinated carbonyl-coupling reactions of iron, including the formation of a complex (TMS)_4_Fe_2_(CO)_8_ have recently been surveyed [578].

A recent book on modern aspects of organo-metallics in organic synthesis has been published [579].

Another book on the logic of chemical synthesis, including topological and stereochemical strategies, has appeared [580].

#### 8.8.5 Other Pertinent Results

Among the various carbon-carbon bond-forming reactions promoted by transition metals, allylic alkylation has been one of the most aggressively sought after. Accordingly, numerous studies have appeared dealing with the chemo-, regio-, and stereoselectivity of coupling between activated allyl substrates (e.g., acetates, carbonates) and nucleophiles, and the application of such reactions in synthesis. Recently, the mechanistic [581] and synthetic [582] aspects of iron-promoted, allylic alkylation have been discussed. Also reported are the iron methylidene complex $(\eta^{5}-C_{5}H_{5}){\left(\text{CO}\right)}_{2}\text{Fe}={\text{CH}}_{2}^{+}$ as an alkylating agent toward coordinated ligands [583], alkylation of tricarbonyl(2-methoxycyclo-hexadienyl)iron^+^ ${\text{PF}}_{6}^{-}$ by substituted anilines [584]; acylation, alkylation, and formation of (η^3^: η^1^-allylacyl)iron complexes [585], and alkylation of the carbide cluster [Fe_4_(CO)_12_C]^2−^ [586].

Other recent carbon-carbon bond-forming reactions including stereoselective reactions are: [Fe(CO)_9_]catalyzed intramolecular dimerization of alkynes [587], novel reaction of limonene-iron (tricarbonyl) [588], synthesis of a donor-stabilized bis(silylene)iron complex [589], and synthesis of a cycloheptatrienyl-bridged iron-iridium complex (η-C_7_H_7_)Fe(CO)_3_Ir(CO)_2_ [590]; also addition of pyridines to the tricarbonyl(cycloheptadienyl)iron(II) cation [591], iron-promoted coupling reaction of homohypostrophene [592], synthesis of alkyl (alkoxycarbonyl) tetracarbonyliron complexes [593], synthesis of a tricarbonyliron complex of 7-azabicyclo [2.2.1] heptadiene derivatives (a nitrene extrusion reaction) [594]; also the reaction of iron(II)-poly(amino carboxylate) complexes with ${\text{CO}}_{2}^{-}$ free radicals (formation of iron-carbon σ-bonds) [595], electroorganic synthesis using Fe(CO)_5_ at atmospheric pressure of carbon monoxide [596], and synthesis of a new type of surface-active cationic iron complexes [597].

New syntheses and reactions of organoiron complexes are recorded in numerous recent publications. Some of them are: synthesis and reactions of η^2^-acrylonitrilecyclopentadienyldicarbonyliron(II) tetrafluoroborate complexes [290,598], synthesis of bis(η^3^-allyl)iron complexes containing basic phosphines [599]; also, synthesis of η^6^-(biphenyl)-η^5^-(cyclopentadienyl)iron(II) hexa-fluorophosphates [600], and synthesis of a new cluster containing CH_3_C = C=CH_2_ triiron-dirhodium ligand [601]. New iron-hydroperoxide reactions (Fenton chemistry) have been recently reported [602].

New reactions including insertion, isomerization, and dimerization are summarized in the following section. The topics selected are: isomerization reactions of terminal iron-alkenyl complexes [603], dimerization of (2,4-dimethylpentadienyl)iron tricarbonyl complexes [604], insertions of cyano- and dicyanoacetylenes into iron-hydride bonds [605], carbonyl insertion reactions and formation of [HFe(CO)_2_]^−^, [(CO)_3_FeCHO]^−^, and [(CO)_4_FeCHO]^−^ [606]; also, carbon dioxide fixation via [(η^5^-C_5_H_5_)Fe(CO)_2_]^−^ metalate Li^+^ or Na^+^ salts [607], reaction of salt $(\eta^{2}-C_{5}H_{5})\text{Fe}{\left(\text{CO}\right)}_{2}^{-}{\text{Na}}^{+}$ with cyclopropylmethyl bromide [608], and reaction of [η^5^-O-dichlorobenzene-(η^5^-C_5_H_5_)iron]^+^ ${\text{PF}}_{6}^{-}$ with phenol [609]. Also covered are reactions of (η^5^-C_5_H_5_)Fe(CO)_2_(η^1^-C_5_H_5_) with bis(tri-fluoromethyl) ketene (to give the 2:1 adducts) [610], reactions of stable mono(alkylthio)carbene complexes of the (η^5^-C_5_H_5_)Fe(CO)_2_ system [611], conversion of a diiron μ-ketone complex into a binuclear cationic complex [612], and reaction of a diiron bridging methylidene complex with an ethylidene complex [613]. Further new reactions are: reaction of [(η^2^-C_5_H_5_)Fe(CO)_2_]_2_(η-C=CH_2_) with HC≡CCN [614], reaction of ${\left[(\eta^{5}-C_{5}H_{5})\text{Fe}{\left(\text{CO}\right)}_{2}\cdot \text{THF}\right]}^{+}{\text{BF}}_{4}^{-}$ with 1,4-dithiane [615], reaction of [Fe_2_(η^2^-C_5_H_5_)(CO)_2_(CNMe)CN(Me)H]^+^ with Ag(I) salts [616], reaction of [Fe_2_(η-C_5_H_5_)_2_ (CO)_4−_*_n_* (CNMe)*_n_* (*n* = O−4) complexes with halogens and mercury(II) salts [617], reaction of Fe(CO)_5_ with *N,N*-diethyl-*S*-ethylcarbamate [618], also, the reaction of Fe_3_(CO)_12_ or Fe_2_(CO)_9_ with 1-diethylaminopropyne [619], the reactivity of [HFe_3_(CO)_11_]^−^ toward alkynes [620] were studied, and thermal decarbonylation of (η^2^-C_5_H_5_)Fe(CO)(PPh_3_)−COR(R = CH_3_,CH_2_SiMe_3_ and CH_2_SiMe_2_Ph) [621].

Complexes obtained by cycloaddition of (η^5^-C_5_H_5_)(CO)_2_Fe(η^1^-CH_2_ CH = CH_2_) with heterocumunlenes such as RN = C=O, RN = S = O or RN = S=NR represent potential precursors to synthesis of heterocyclic organic compounds [622–624].

Other recent interest centered on organoiron compounds involves physical, spectral, and thermodynamic studies. This area of interest covers: reaction of gas-phase iron(I), e.g., FeCO^+^ with alkyne and dienes [625], the gas-phase ion chemistry of the (C_4_H_6_)Fe(CO)_3_ complex [626], the redox chemistry of alkyne-Fe_3_(CO)_9_)(RC_2_R) cluster [627]; also, spectroscopic study of iron tetracarbonyl isocyanide complexes [628], an alkali ion effect on hydride migration in [HFe(CO)_4_)]^−^ [629], potassium permangante oxidation of η^6^-arene (η^5^-C_5_H_5_)iron cations (to yield ketones and sulfones) [630], and spectroscopic and kinetic studies of ligand substitution in the 33e dinuclear radical Fe_2_(CO)_7_(η-PPh_3_) [631].

Other recent studies report on x-ray structure of (*N*-heterocyclic)(η^4^-diene)-dicarbonyliron(O) compounds [632], theoretical study of tricarbonyliron complexes of *para*-quinodimethane [633]; labile benzene-iron complexes; crystal structure of *cis*- and *trans*-FeH1(CO)_2_(PPh_3_)_2_ [634]; also, a carbon basicity scale based on the cation [(η^5^-C_5_H_5_)Fe(CO)_2_(C_2_H_4_)]^+^ [635], and the crystal structure of the novel di-iron-anthracene complex Fe_2_(η, η^3^, η^3^-anthracene)(CO)_6_ [636].

Recent pertinent topics also include a review of organic reactions of π-complexes of transition metals [637]; a review of the photochemical reactions of metal carbonyl complexes of transition metals with conjugated and cumulated dienes [638]; spin-crossover complexes of Fe(II) and Fe(III) [639]; synthesis and reactions of anionic metallocarboxylates of iron, e.g., M[(η^2^-C_5_H_5_)Fe(CO)(PPh_3_)(CO_2_)](M=K, Li) [640]; synthesis of a macrobicyclic iron(III) sequestering agent [641] and construction of models for iron-oxo proteins (e.g., mixed valence iron cluster) [642–644]; the importance of iron-oxo aggregates in biological cell systems [645–647]; organoiron-mediated oxygenation of allylic organotin compounds as a possible chemical model for enzymic lipoxygenation [648]. The interaction of carbohydrates with metals has been of much interest in recent years and progress in this direction is on the horizon [649–653].

**Scheme 64:**
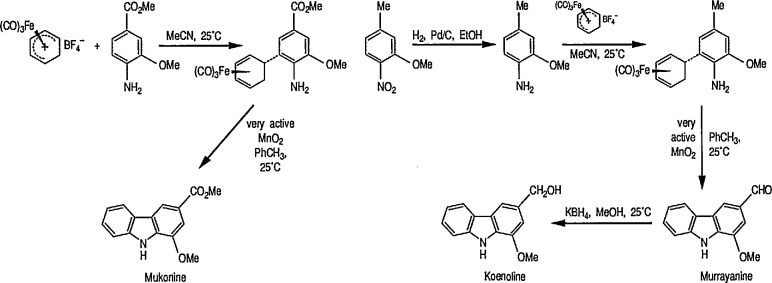
[28]

**Scheme 65:**
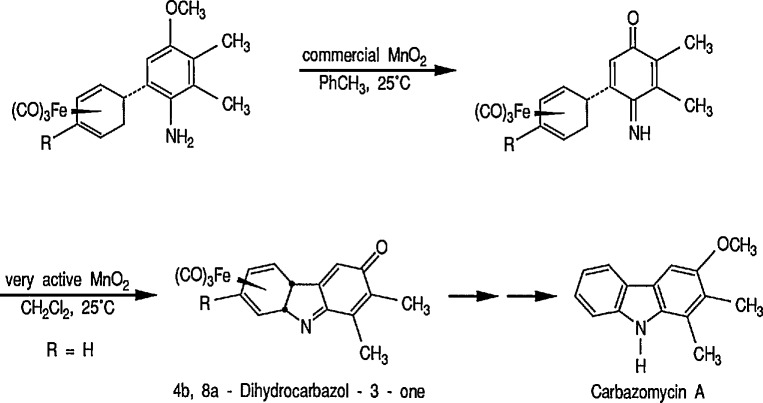
[28]

**Scheme 66:**
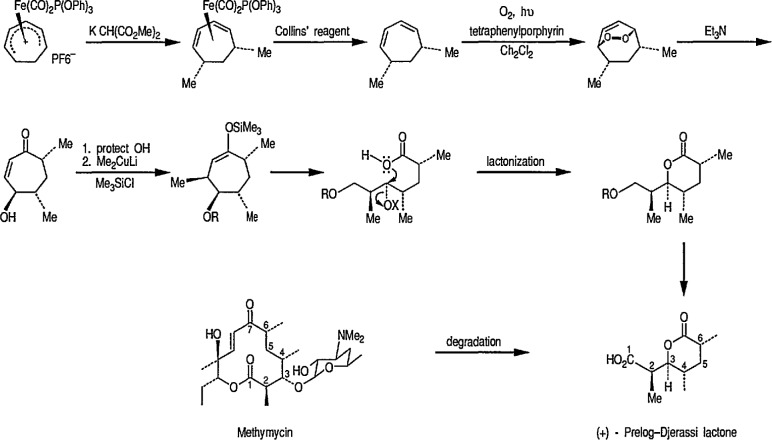
[393]

Some additional methods that may be of interest in the synthesis of natural products are depicted next (Scheme 64–66).

The total syntheses of the alkaloids mukonine, murrayanine, and the cytotoxic carbazole koenoline by an iron-mediated arylamine cyclization with very active manganese dioxide are shown in Schemes 64 and 65; it may be noted that there is a specific action of very active MnO_2_ as compared to commercial MnO_2_ [28].

A successful synthesis of an optically active (+)-Prelog-Djerassi lactone (a degradation product of the macrolide antibiotic methymycin) involves a 13-step procedure, beginning with an iron stereodirecting template attached to 1,3-cycloheptadiene, and shown in Scheme 66 [393].

## 9. Addenda

Selected highlights regarding organoiron complexes from the recent literature are presented next.

### 9.1 Mononuclear Iron Complexes. Iron Carbene Complexes

Electrophilic transition-metal-carbene complexes LnM = CHR (R = H, alkyl, aryl) (M = Fe, Cr) are much less stable than classical. Fisher-type heteroatom-substituted carbene complexes, especially where the organometallic fragment is not electron-rich. For example, the complexes Cp(CO)_2_Fe = CH_2_ and Cp(CO)_2_Fe = CHCH_3_ (Cp = η^5^-C_5_H_5_) are implicated as reactive intermediates. These highly electrophilic species may be stabilized either by substitution of better donor ligands for the π-acid CO ligands or by substitution, at the carbene atom, of hydrocarbon substituents which are effective at delocalizing positive charges [66,654].

Indeed, electrophilic iron-carbene complexes of the general type Cp(CO)(L)Fe = CHR^+^ (L = CO, PR_3_) readily transfer the carbene moiety to alkenes, to form cyclopropanes; and this transfer reaction has recently been applied to enantioselective syntheses of cyclopropanes [226,655–657]. The development of enantioselective methodologies for acyclic, multiple stereocontrol continues to present an important challenge for synthetic chemists [270,280]; and recent reports describe several highly diastereoselective reactions [658–664].

Metal-carbene complexes are also useful reagents for the selective formation of carbon-carbon bonds and the synthesis of organic compounds [167]. The dimerization of carbene ligands, with carbon-carbon double-bond formation, is of special interest for the direct access it provides to functional alkenes displaying specific properties [137,167]. As for carbene-alkene coupling reactions or metal-carbene-initiated alkyne polymerization, the initial step of the carbene ligand dimerization process involves the generation of a vacant site by thermal or photochemical dissociation of a metal-ligand bond. In addition, it has been established that the thermal dimerization does not progress with the release of the uncoordinated carbene species but via a bimetallic intermediate [665].

The chemistry of transition-metal of vinylidene (or alkylidene) complexes has recently attracted considerable attention, and the topic has been extensively reviewed [666]. The vinylcarbene complexes are postulated to be key intermediates in the reaction of carbene complexes with acetylene involving the Dötz reaction [74] and polymerization of acetylenes. These processes sometimes involve “insertion” of carbon monoxide into the carbon-metal double bond of the vinylcarbene complexes, resulting in the formation of coordinated vinylketenes [667]; this reaction has recently been studied in detail for the formation of (η^3^: η^1^-allylacyl)tricarbonyliron [668].

On the other hand, insertion of carbon monoxide into a transition-metal-carbon single bond (or the migration of an alkyl group on a coordinated carbon monoxide molecule) is one of the most important and fundamental reactions affecting carbon-carbon bonds in catalytic or noncatalytic organic synthesis by transition metals [669]. Other recent applications of iron-carbene complexes have been reported [665,670–678] and related carbyne complexes have been discussed [679].

### 9.2 Dinuclear Iron Complexes

Dinuclear transition-metal complexes can be considered to be the simplest model of metal surfaces, and they have the potential, through cooperativity of metal centers, to effect unique transformations of simple organic substrates not possible by a single metal center [680]. Heterodinuclear complexes are particularly interesting in this regard, because each metal may be able to effect a reaction that is not possible for the other, or the two metals in concert could facilitate a transformation unavailable to either metal alone.

The study of reactivity of organic molecules linked to dinuclear transition-metal (e.g., diiron) complexes has received a great deal of attention during the past 10 years. α-Diimine ligands, RN = CHCH = NR, are known to show very versatile coordination behavior, resulting from the possibility of donating from two to eight electrons, via the N lone pairs and the C = N π-electrons, to the metal center to which they are bonded [681,682].

In certain dinuclear-metal complexes, metal carbonyl anions have been used to form metal-metal bonds to complexes containing carbene ligands [683,684]. For example, the reaction of the cationic iron complex FeCp(CO)(NCMe)[C(SMe)_2_]^+^ (Cp = η^5^-C_5_H_5_), which contains bis(thiomethoxy)carbene ligand, with $\text{Co}{\left(\text{CO}\right)}_{4}^{-}$ led to facile replacement of the labile NCMe ligand and formation of a Co–Fe bond [683]. The product contained a bridging η^2^-C, S-coordinated carbene ligand, with a sulfur atom bonded to the cobalt atom.

There has been growing interest in the synthesis and characterization of polymetallic complexes whose metal centers are joined by polyfunctional organic ligands having delocalized π systems [685]. This class comprises diiron, triiron and mixed iron complexes. The series includes a number of arene-bridged iron complexes [686–689]; also iron complexes having allene [690], alkyne [691], ylide [692], ketyl [693], carbyne [694], azoalkane [695], and similar organic bridges [696–705]. The cationic diiron bridging methylidene complex ${\left[\left(\eta^{5}{-C}_{5}H_{5}\right)\left(\text{CO}\right)\text{Fe}\right]}_{2}\left(\eta -\text{CO}\right){\left(\eta -\text{CH}\right)}^{+}{\text{PF}}_{6}^{-}$ is extremely electrophilic, and reacts with CO to give a 1:1 adduct, ${\left[\left(\eta^{5}{-C}_{5}H_{5}\right)\left(\text{CO}\right)\text{Fe}\right]}_{2}\left(\eta -\text{CO}\right){\left(\eta -\text{CHCO}\right)}^{+}{\text{PF}}_{6}^{-}$. This complex, best regarded as a bridging acyhum species, is readily attacked by nucleophiles at the acylium carbon atom [706]. Recently, a new, acid-catalyzed, *trans* to *cis* isomerization of the diiron methylidene complex has been reported by the same group [707].

Organometallic dinuclear complexes have been shown to be able to couple coordinated carbonyls. In this regard, synthetic organic chemists have benefited greatly from the use of these “inorganic” systems to effect reductive carbonyl coupling in which a carbon-carbon bond is created from two carbonyls [708].

### 9.3 Iron Cluster Complexes

The chemistry of metal cluster complexes containing heteroatom-substituted carbene ligands has recently been surveyed [709]; bonding in transition-metal clusters has been discussed [710]. There has been much recent interest in catalysis by metal clusters [711]. The preparation of transition-metal cluster carbonyl complexes has recently received increasing attention because of their potential for CO reduction. In a number of cases, the presence of different metal centers in the same molecular unit enhances the chemistry of the individual species as compared with their mononuclear analogues [712]. The presence of two or more metal centers may lead to unique reactivity as a result of metal-metal or metal-ligand-metal interactions that can readily accomplish otherwise difficult transformations, leading to new catalytic processes [713]. There has been considerable recent interest in the synthesis of clusters that provide a conceptual bridge between organometallic chemistry and the area of inorganic solid-state chemistry and surface chemistry [714]. In series of iron clusters, this class includes alkylidene [715], acetylene [716–718], carbide [719], and phosphaalkyne [720] bridging ligands. Recent work on mixed-metal clusters comprises Fe-Hg [721], Fe-Sn [722], F-Ge [723], Fe-Co [724], Fe-Ru [725], and Fe-Pt [726] clusters. A novel coordination behavior of ynamine ligands RC = CNR_2_ in diiron cluster complexes has been described [727,728]; some of these clusters incorporate carbene ligands [728]. A new cluster chemistry also includes skeletal rearrangement of [Ru_2_Fe_4_(CO)_16_B]^−^ cluster anion [729] and the chirality and optical activity of cluster complexes [730].

### 9.4 Iron-Main Group Element Complexes

Recent advances in the chemistry of mixed main-group element-transition metal complexes or clusters has revealed the important role played by the main-group elements [731–733]. The interactions of the main-group elements, for example, group IVA (silicon, germanium, and tin) or group VA (nitrogen, phosphorus, arsenic, antimony, and bismuth), or group VIA (oxygen, sulfur, and selenium) with iron carbonyls produced a number of new heteroatomic complexes and clusters. Intriguing structures and bonding patterns have been observed in these complexes.

From among the main-group elements, of special interest are complexes incorporating the phosphorus-iron bond [734–736]; this includes the important stereo-electronic properties of phosphorus(III) ligands [737,738].

The separation of phosphorus(III) ligands into two distinct groups identified as pure σ-donor ligands and σ-donor-π-acceptor ligands for the acetyl and methyl complexes (η^5^-C_5_H_5_)FeL(Co)Me has recently been accomplished [738].

Phosphorus compounds having a low coordination number have been the subject of considerable interest ever since they were first described. One of the showpiece examples is *tert*-butyl-phosphaacetylene or 3,3-dimethyl-1-phospha-1-butyne; these can be incorporated into heterocycles, used as new ligands, or cyclooligomerized by Fe_2_(CO)_9_, to yield a new, mixed phosphocyclopentadieneiron complex [739].

The complexation of transition-metal complex fragments to compounds in which phosphorus is involved in multiple bonding has been intensively investigated in recent years. Both terminal (η^1^) coordination, as found for phosphanes, and side-one (η^2^) coordination, as found for alkenes and alkynes, have been observed. A recent report [740] describes the first diphosphaallyl complex in which, in addition to the η^3^ coordination of the tricarbonyliron fragment via the lone pairs of two phosphorus atoms, a further metal atom is bonded.

Transition-metal complexes containing a η^5^-cycIopentadienyl group (Cp) and three monodentate ligands that may incorporate a phosphorus ligand are referred to as “piano-stool” complexes because of their molecular shape. Recently, reactions of cyclopentadienyl complexes have been reported in which one of the monodentate ligands on the transition metal undergoes base-induced migration to the Cp ring: silyl from iron [741], acyl from iron [742,743], and alkoxycarbonyl from iron [744]. Migration of the phosphorus ligand from iron to the Cp ring in complexes of the type (η^5^-C_5_H_5_)(CO)LFeP(O)YZ (L=CO,Y = Z=OEt) has been reported for the first time [745]; synthesis of the four-legged piano stool complexes of iron are also described [746]. The mechanism of phosphine migration from a metal to a carbon site in a trimetallic cluster, e.g., anion [Fe_2_Co(CO)_8_(PR_3_)(CCO)]^−^ has been investigated [747].

Further challenging chemistry involves complexes comprising silicon-, arsenic-, or sulfur-iron σ bonds, groups IVA, VA, and VIA, respectively. Selected from the main group IVA are silicon-iron [748–752], silicon-iron and silicon-germanium [753], germanium-iron [745], and tin-iron [755] complexes. Selected from group VA are nitrogen-iron [756,757], phosphorus-iron [758–764], phosphorus-arsenic-iron [765], and tin-iron [766–768] complexes. Complexes containing iron and main-group elements from group VIA have also attracted considerable interest; these are sulfur-iron [769–786], sulfur-selenium-iron [787], and selenium-tellurium-iron [788] complexes.

#### 9.4.1 Iron-Sulfur Clusters

Many of the electron-transfer processes of cellular biochemistry are mediated by redox en2ymes containing iron-sulfur centers, the two most important being [2Fe-2S] and a cubene-type cluster [4Fe-4S] [789]. These iron-sulfur clusters are widely distributed both in plant and animal tissues, and they have been found to react very readily with nitrite. Thus, vegetative cells of *Clostridium botuUnum*, which contain [4Fe-4S] centers, were found [790] to react with nitrite, destroying the iron-sulfur cluster and simultaneously forming paramagnetic dinitrosyliron complexes of the type [Fe(NO)_2_X_2_]^·+^ (X = cysteinate). Thus, there is a clear association between the ingestion of high levels of nitrite, the formation of cation-radical complexes [Fe(NO)_2_X_2_]^·+^, and the existence of cancerous states in experimental animals [789]. A special recent interest has been placed on the cubane-type iron-sulfur, clusters containing the [4Fe-4S]^+^ core oxidation level [791], the heterometal cubane-type clusters [MFe_3_S_4_L*_n_*_=3–5_]^1− to 4−^ (M = Mo,W) with anionic terminal ligands [792,793], or cationic iron-sulfur clusters [VFe_3_S_4_]^2+^ and [MoFe_3_S_4_]^3+^ [794].

The reaction of CO_2_ to give useful materials is an important chemical reaction having biological implications. And this is now achieved with iron-sulfur clusters. An efficient electrochemical fixation of CO_2_ into formate was recently carried out by using an [Fe_4_S_4_] cubane cluster catalyst bearing a 36-membered methylene backbone, in *N*,*N*-dimethyl-formamide [795].

A recent interest has also been exhibited in the products generated in iron-sulfur cluster self assembly systems; for example, the synthesized cluster [Fe_6_S_6_(PEt_3_)_6_]^+^ has a unique, basket-type of stereochemistry [796].

### 9.5 Oxo-Bridged Iron Complexes

#### 9.5.1 Novel Iron-Based Catalyst for Dioxygenation

The chemisorption of oxygen, and reaction pathways at metal surfaces, as well as the role of surface oxygen, are the topics of a recent survey [797].

A homogeneous, iron-based catalyst, e.g., [bis(2,6-carboxylpyridine)iron(II)] has recently been found [798] to activate dioxygen to ketonize methylenic carbon atoms or dioxygenate acetylenes, arylalkenes, and catechols. For example, the catalyst transforms cyclohexane into cyclohexanone and diphenyl-acetylene into benzil, in what is believed to be the first such one-step process using dioxygen under ambient conditions. For the dioxygenation of unsaturated α-diols, such as catechol, the system parallels the action of catechol dioxygenase enzymes. Hence, the reactive intermediate of the reaction may be a useful model and mimic for the activated complex of dioxygenase enzymes.

#### 9.5.2 Biological and Enzymatic Application of Oxo-Bridged Iron Clusters

Oxo-bridged polyiron complexes (clusters) are usually taken to involve a bridging oxygen atom derived from an oxo ligand, but the term also refers generically to species having bridging OH^−^ or OR^−^ ligands. Oxo-bridged clusters of iron are important structural and functional units of many redox enzymes [799–801]. In recent years, the binuclear iron-oxo center has emerged as a common structural component in the active sites of several metalloproteins [799,802,803]. These centers have important functional roles in hemerythrin [804], ribonucleotide reductase [805], methane monooxygenase [806], and the purple acid phosphatases [802,807], including reduced uteroferrin [808]. The binuclear center in such proteins is known or is postulated to exist in either an oxidized Fe(III)-Fe(III), a reduced Fe(II)-Fe(II), or mixed Fe(II)-Fe(III) form.

In recent years, a steadily increasing number of oxo/hydroxo-bridged clusters of iron(III) have been synthesized and structurally characterized. Complexes of nuclearity Fe_3_, Fe_4_, Fe_6_, Fe_8_, and Fe_11_ have been investigated in considerable detail [799]; the study by the same group includes synthesis of a novel oxo-bridged dinuclear iron(III) complex containing only oxygen-donating ligands [809], and assembling of phosphate ligand-oxo-bridged diiron(III) proteins [810] and asymmetric oxo-bridged diiron(III) complexes [811].

A new class of iron proteins having dinuclear iron centers bridged by oxide and carboxylate groups has emerged [799,802]. Structural and spectroscopic models of the (η-oxo)bis(η-carboxylato)diiron(III) center found in the marine invertebrate respiratory protein hemerythrin have been synthesized with a variety of facially coordinated tridentate ligands [812]. In accord with this trend, a new class of [Fe_2_O(O_2_CR_2_)]^2+^ model complexes having terminal bridging dicarboxylate ligands [813] or a (η-oxo)(η-carboxylate)diiron(III) complex having distinct iron sites [814] have recently been assembled. Recent related studies on metalloproteins and metalloenzymes include the synthesis of the lactoferrin analog [LFe(η-O)(η-CO_3_)FeL]·4.25H_2_O (L=1,4,7-trimethyl-1,4,7-triazacyclononane) [815], the interaction of the *N*-[2-(o-hydroxyphenyl)glycino)ethyl]salicylideneamine iron(III) complex with catechol [816], iron-activated alcohol dehydrogenase [817]; also, the chemistry of ferro- and ferri-verdins derived from microorganisms [818], evidence for water coordinated to the active-site iron in soybean lipoxygenase-1 [819], and a structural study of the molybdenum-iron protein of nitrogenase [820]. Other reported studies are the oxidative cleavage of (η-oxo)iron(III) tetraarylporphyrins [821], cleavage of DNA by a binuclear iron(III)-peroxide adduct [822], and a possible model of hemoprotein-hydrogen peroxide complex [823]; also oxo-bridged iron(III) complexes containing substituted benzothiazole and benzimidazole ligands [824], proton-coupled electron transfer in oxo-bridged clusters of iron and manganese [825], and the geometry of binuclear transition-metal complexes containing two paramagnetic metal ions joined by an oxo bridge [826].

### 9.6 Photochemistry of Organoiron Complexes

The electronically excited-state lifetimes of organometallic compounds in solution are typically very short. Single electron-transfer reactions of organometallic complexes are known to be initiated by their irradiation with visible or UV light in the presence of an appropriate ligand [827,828] or reagent [829,830]. For example, the photochemistry of the cationic [(η^5^-C_5_H_5_)Fe(ArH)]^+^ X^−^ complexes has been explored from both synthetic and mechanistic viewpoints. Their irradiation in a ligand-containing solution leads to release of the arene, and formation of a new, triply ligated iron(II) complex [831]. However, irradiation of similar complexes, e.g., (2-naphthylmethyl)triphenylborate [NpCH_2_B(Ph)_3_]^−^ salts of [η-C_5_H_5_)Fe(ArH)] cations in tetrahydrofuran solution generates the 2-naphthylmethyl free-radical, e.g., NpCH [832].

The photochemistry of iron piano-stool dimers has been studied [833]. Generally, dimers of the type (η-C_5_H_5_)_2_Fe_2_(CO)_2_(η-CO)_2_ have a rich and diverse photochemistry [834,835], and the photochemical insertion (by laser flash-photolysis) of alkynes into those dimers (to yield diironcyclopentenones) has recently been described [836].

Other selected photoreactions of iron complexes include syntheses [837,838], elimination of the CO ligand in (η^5^-C_5_H_5_)Fe(CO)_2_(η^1^-CH_2_C_6_H_5_) [839], α-elimination of carbon (to give metal π-complexes) [840], disproportionation of (η^5^-C_5_H_5_)Fe(CO)_4_ [841], rearrangement of (η^5^-C_5_H_5_)Fe(CO)_2_-substituted oligosilanes [842], substitution of CO ligand in [Fe_2_(CO)_6_(L)P(*n*-Bu_3_)] (L=2,2′-bipyridine) [843], and breaking of the iron-nitrogen bond in [Fe(CO)_3_(*i*-Pr)_2_(1,4-diaza-1,3-butadiene)] [844],

### 9.7 Gas-Phase Reaction of Organoiron Complexes and Iron Ions

Gas-phase inorganic chemistry focuses on the chemical reactions of metal ions and metal clusters and on the study of these species by using modern spectroscopic methods and Fourier-transform mass spectrometry (FTMS) [845]. Gas-phase organometallic chemistry derives its interest from the fact that, by the very nature of the physical isolation, the inherent properties of specific metal ions (or complexes) can be probed in the absence of other disturbing influences. These include ligand and solvent effects, as well as ion pairing, which prevail in the condensed state. The transition metal-ligand bond has been described as being the key to linking organometallic chemistry, surface chemistry, and catalysis. In particular, metal-ligand bond energies are useful in assessing whether a proposed reaction-pathway is energetically feasible. To provide such quantitative data, a growing number of mass-spectrometric techniques have been developed. With regard to the understanding of many catalytic processes, a fundamental problem is concerned with the mechanism of activation of C–H and C–C bonds, which constitutes a crucial step in catalytic cycles [846,847] and which is also observed in the reactions of bare transition-metal ions with organic substrates in the gas phase [848–850].

Recently, several reactions in the gas phase have been reported; these are reactions of iron ions with allenes [851], reaction of 1-alkanols [852] or linear and α-branched aliphatic nitriles [853] with bare iron ions; also, reaction of [FeCH_3_]^+^ with nitrogen-containing species [854], reaction of iron-alkyl complexes (α-hydrogen migration) [855], reaction of phenyl halides with iron ions (formation of oligomer) [856], and protonation and decomposition of [(η^5^-C_5_H_5_)(CO)Fe]_2_(η-CO)(η-C = CH_2_) [857].

### 9.8 New Ferrocene Chemistry. Novel Multiple-Decker Ferrocenes

Organotransition-metal chemistry has, since the discovery of ferrocene in 1951, attained considerable importance in organic synthesis, in both stoichiometric and catalytic processes. In the meantime, classical ferrocene chemistry has also undergone significant transformation and new chemistry has emerged.

Recent interest in ferrocene chemistry concerns a new development of polymetallocenes [858], namely, the one-dimensional stacking of alternating metal and ring. Thus, the versatile chemistry of metallocenes has indeed catalyzed the study of new monomeric, dimeric, and oligomeric metal complexes obtained by sandwiching additional metal-ring combinations.

Addition of a ring, e.g., a double-layered organic π-ligand to ferrocene may lead to various polymeric metal complexes whose π-systems interact strongly, thus allowing efficient metal-metal communication. Three types of double-layered organic π-ligands have been utilized for the construction of columnar polymeric ferrocene complexes; these π-ligands are cyclophanes [859,860], indenophanes [861], and sterically fixed, naphthalene-bridged cyclopentadienyl systems [862], Addition of a metal and a ring to ferrocene leads to triple-decker sandwiches, a new and popular topic of research [863]. The number of valence electrons in triple-decker sandwich complexes is 30 [863]. Addition of one metal and ring to a triple-decker complex leads to tetradecker sandwiches [864]. The number of valence electrons in these complexes varies from 40 to 46. Higher homologs of ferrocenes [863,865,866] and polymetallocenes [859,864] provide exciting challenges.

Several new reactions of ferrocenes and substituted ferrocenes have been reported [867,876], including interesting photochemical conversion of (η^1^-butadinyl)iron complexes to hydroxyferrocenes [877], photolysis of (arene–C_5_H_5_)ferrocene to a novel (C_5_H_5_–Fe) complex [878], and new chiral ferrocenylphosphines having C_2_ symmetry [879].

#### 9.8.1 Pseudo-Ferrocenes, A New Class

Pseudo-ferrocenes are complexes wherein a bridging, cyclic π-ligand (a 6π-electron donor) is arena, fulvalene, or heterocyclic, in a sandwich coordination with the cyclopentadiene ring. Bis(arene) or other mixed-ring sandwich complexes, and substituted open and half-open ferrocenes also belong to this new class.

Over the past several years there has been growing interest in the synthesis of polymetallic complexes whose metal centers are joined by polyfunctional organic ligands having delocalized π-systems [685,880]. The localization of an electron inside a three-dimensional, molecular framework is a key property of electron-reservoir complexes, and this is another topic of recent interest in the ferrocene field [881]. The π-complexation of aromatics, polyaromatics, or heteroaromatics via sandwiching by a transition metal brings about a tridimensionality which should have a dramatic influence on the physical properties, and could open up new synthetic routes. As compared to simple ferrocenes, sandwiched aromatic (e.g., polyaromatic) complexes can have a weaker arene-metal bond, which is useful in catalysis and for any purpose requiring free coordination sites. By applying pseudo-ferrocenes, a new vista of chemistry has recently been discovered. This chemistry includes arene-ferrocenes [882], bis(arene)-ferrocenes [883,884], naphthalene-ferrocenes [885], polyaromatics-ferrocenes [886]; also, heptafulvalene-ferrocenes [887], bis(fulvalene)- and arene-fulvalene-ferrocenes [888,889], cyclooctatetraene-ferrocene [890], (1,2-azaboride)-ferrocene [891], pentaphosphaferrocene [892]; also, tentacled iron sandwich complexes [893], tetracarbonyl ferrate-(η-arene)Cr(CO)_3_ complex [894]; substituted open, half-open, and closed ferrocenes [895], half-open ferrocenes [896], spiro (closed)-ferrocenes [897], a porphyrin-ferrocene-quinone complex [898], and ferrocene-substituted high polymeric phosphazenes [899].

In contrast to the alkali-metal coordination studies of electrochemically reducible redox-active macrocyclic systems, until quite recently little had been reported about the effects of cation binding on the oxidation potential of redox-active macromolecules containing an oxidizable moiety such as ferrocenyl, e.g., ferrocene crown and biscrown ethers [900].

### 9.9 Iron Porphyrins

Iron porphyrins constitute a widely studied class of complexes because of their prevalence in biological systems, notably the iron protoporphyrin IX (heme) proteins [555,901,902]. The extended π-orbital network of these macromolecules also leads to applications in electronics and solid-state chemistry. Particularly, porphyrin derivatives play a crucial role in many chemical and biological electron-transfer reactions [901]; these include iron(IV) porphyrin as a model for the peroxidases [903].

Metalloporphyrins can also mimic enzymes in reactions. As recently reported [904], the protonation of transition-metal porphyrin hydrides has yielded the first known dihydrogen complex of a metalloporphyrin and a system that performs some functions of hydrogenase enzymes.

Several aspects of metalloporphyrin chemistry, including the active-site structure and reactivity, have recently been investigated. The success of these studies often depends upon the unique steric properties of the porphyrin employed. Steric control at the metal site has been facilitated by the use of capped, single- and double-strapped [905,906], bis-pocket, picket-fence [907], and basket-handle [908] type porphyrins. In many cases, the polarity and hydrogen-bonding properties of the porphyrin substituent stabilize the coordination of an axial ligand, such as dioxygen, by the metal ion [905,906]. A new porphyrinatoiron complex, 5,10,15,20-tetrakis(2,6-bispivaloyloxyphenyl)porphyrinatoiron(II), has been synthesized; axial-base ligation was sterically depressed by the four ester groups on each side of the porphyrin, and a stable dioxygen adduct was formed reversibly at 25 °C in toluene [909].

Recent applications of iron porphyrins cover fields of biochemistry [910,911], medicine [912,913], and chemistry [914–917], including asymmetric hydroxylation by a chiral iron porphyrin [918].

The reactions of metalloporphyrins have also received particular attention in relation to biological systems, including cytochrome P-450, cytochrome C, or cytochrome b_5_ [556,920]. Because reactions take place as for the substrate and/or the reagent coordinated to the metal on a rigid macrocycle of porphyrin, notable stereospecificities are expected, and, in fact, results of some investigations into stereochemical aspects have been used in exploiting metalloporphyrins as cytochrome P-450 model systems [556,919,921,922]. An iron porphyrin unit functions as the redox site in many electron-transfer enzymes. A sub-section of this class of enzymes has the iron with either one or two axial imidazole ligands; in the cytochrome C group, the porphyrin is attached to the peptide chain by sulfur bridges between cysteine residues and the two porphyrin vinyl groups [923]. Recently, the reaction between the cytochrome-C-derived heme octapeptide microperoxidase (MP) and hydrogen peroxide leading to the formation of hypervalent iron-oxo-porphyrin cation-radical [924], the generation of the Fe(III)-OEP-H_2_O_2_ complex (OEP = octaethylporphyrinato) [925], or of the Fe(III)TPP-H_2_O_2_ adduct (TPP = tetraphenylporphyrinato), all important in electron-transfer in a novel synthetic membrane analog for cytochrome C [926], have been reported.

### 9.10 Additional Recent Results

Some important recent reviews include organoiron chemistry, an annual survey for the year 1988 [927], enantioselective catalysis with metal complexes [928], multiple stereocontrol using organometallic complexes as applied in organic synthesis [929], and new synthetic applications of cyclopropanes containing C_5_- building blocks [930], There are new books on stereoselective synthesis [931], metal-DNA chemistry [932], and electron-transfer between metal centers, including biological systems [933].

#### 9.10.1 New Carbon-Carbon Bond-Forming Reactions. New Seven-Membered Ring Iron Complexes

Among the various carbon-carbon bond-forming reactions promoted by iron, functionalization of a seven-membered ring via (cycloheptatriene)iron tricarbonyl has been one of recent synthetic interest. Thus, deprotonation of (cycloheptatriene)Fe(CO)_3_ provides a species that can be considered to be the antiaromatic cycloheptatrienide anion stabilized by a transition-metal fragment [934]. This deep-red organometallic anion can be considered in terms of two limiting bonding alternatives, one of which delocalizes the negative charge in the ring as an allylic carbanion, and one that places the charge density on the metal [935]. Thus, the (η^5^-C_7_H_7_)Fe(CO)_3_ anion has been used to prepare a number of heterobimetallic complexes linked by either a metal-metal bond or through the seven-membered ring [936]. This anion is also an attractive substrate for the regio- and stereocontrolled elaboration of seven-membered rings, particularly because of its reactivity with organic electrophiles and their derivatives (e.g., acid chlorides [935] or in the synthesis of natural products [929]. The reaction of the cycloheptadienyl cation $\left(\eta^{5}-C_{7}H_{9}\right)\text{Fe}{\left(\text{CO}\right)}_{3}^{+}$ with KI [to give (η^5^-C_7_H_9_)Fe(CO)_2_I] [937], and the chemistry of the related (η^3^-pentadienyl)iron tricarbonyl halides [938], have been studied. Also, an optically pure(tropone)iron tricarbonyl [939] useful for the synthesis of natural products [940] has been prepared. The synthesis and separation of the [(tropone)Fe(CO)_2_(+)-neomenthyldiphenylphosphine] diastereoisomer of 6S planar chirality has recently been accomplished [941].

Other new carbon-lengthening methods are the reaction of KHFe(CO)_4_ with an excess of ethyl acrylate in ethanol (to give ethyl propanoate and diethyl 4-oxopimelate) [942], the coordination of the dinitrogen complex η-N_2_[Fe(CO)_2_L_2_]_2_ (L=P(OMe_3_); Pet_3_) with diphenylketene diphenyl-*p*-tolylketene imine to form η^2^-C,O ketene compounds Fe(CO)_2_L_2_Ph_2_C_2_O(L=P(OMe)_3_; Pet_3_) [943], the reaction of iron(II) acetylide complexes with diphenylketene, diketene, and 1,3-dicyano-1,3-butadiene to produce the corresponding [2 + 2] and [4 + 2] cycloadducts [944], the reaction of an electrophilic iron carbene complex [(η^5^-C_5_H_5_)(CO)_2_Fe = CHAr]^+^(Ar = p-C_6_H_4_OMe) with nitrosoarenes O = N-Ar′ (Ar′ = C_5_H_5_, p-C_6_H_4_NMe_2_) or azobenzene PhN = NPh (to cause insertion of the ArN = X moiety into the Fe = CHAr bond, by formation of a nitrene complex) [945]; also, insertion of isocyanates into M–H bonds is illustrated by the reaction of (η^5^-C_5_H_5_)HFe(CO)_2_ with *t*-BuNCO to give (η^5^-C_5_H_5_)Fe(CO)_2_[C(O)NH(*t*-Bu)] [946] or by formation of pentakis(trifluoromethyl isocyanide)iron, Fe(CNCF_3_)_5_ [947]; also, the synthesis of (methyl-2-butenoate)iron complexes by the reaction of (η^5^-C_5_H_5_)Fe(CO)_2_Na with methyl-4-chloro-2-butenoate) [948].

Other recent work involves an interesting transformation of the cluster Fe_3_(CO)_10_(η^3^-PR) into two carbene-functionalized clusters under the conditions of Fischer-type carbene synthesis. The first cluster thus obtained contains a terminal carbene ligand R′COR″, whereas the carbene ligand in the second cluster coordinates to two metal atoms via the carbene atom, and to the third iron center through the oxygen atom of the R″O substituent [949].

Other recent studies examined rearrangements of cyclopropanes σ-bonded to iron [950], iron-mediated diene activation [951], σ,π complexes of benzene, e.g., (η^6^-C_6_H_6−_*_n_*Fp*_n_*)Cr(CO)_3_(Fp = (η^5^-C_5_H_5_)Fe(CO)_2_ and *n* =2 or 3) [952], synthesis of [(η^5^-C_5_H_5_)(CO)_2_FeCH_2_]_3_CH complex and the study of hydride transfer from carbon-hydrogen bonds therefrom [953]; also, catalytic alkylations of allylic carbonates in the presence of nitrosylcarbonyliron complexes [954], the electrochemical oxidation of (η^5^-C_5_H_5_)(CO)_2_FeMe in acetone [955], *N*-analogues of metal acetylacetonates:bis(1,2,6,7-tetracyano-3,5-dihydro-3,5-diimino-pyrrolizinido) iron(II) [956], metalladiboranes of the iron group: K[Fe(CO)_4_(η^2^-B_2_H_5_)] [957], and fluorine-substituted ferracyclopentadiene complexes with an unprecedented fluorine bridge between boron and carbon [958].

#### 9.10.2 Tripodal Polyphosphine Metal Complexes

Over the past decade, tripodal polyphosphines have proved to be useful and versatile ligands in inorganic and organometallic chemistry [959–961]. Recently, transition-metal complexes of tripodal polyphosphines have begun to attract interest because of their potential as catalysts in several homogeneous reactions, including (a) hydrogenation of alkynes, alkenes, and organic nitriles, (b) hydroformylation and isomerization of alkenes; (c) polymerization of alkynes, (d) oxidation of inorganic and organic substrates, and (e) synthesis of vinyl ethers from terminal alkynes and carboxylic acids [962]. The principal reasons explaining why tripodal polyphosphine ligands participate in such a wide range of catalyst systems can be summarized under six main headings [962,963]; (i) excellent bonding ability, (ii) strong *trans* influence, (iii) formation of stable complexes in a variety of metal oxidation states, (iv) great control of the stereochemistry, (v) adaptability to many different coordination numbers and (vi) high nucleophilicity of the metal centers.

There is ongoing interest in other iron complexes incorporating diphenylphosphino ligands. Thus, the nitrosyl dimer [Fe(NO_2_)Cl]_2_ in the presence of 1,2-bis(diphenylphosphino)ethane (dppe) yields the complex [Fe(NO_2_)Cl]_2_ (η-dppe); this is the first structurally characterized binuclear complex where dppe constitutes the single bridge between two metallic centers without the presence of a metal-metal bond [964].

It is now recognized that the ease of carbon—phosphorus bond cleavage at metal centers is dependent upon the hybridization of carbon, following the order: P–C (sp) > P–C (sp^2^) > P–C (sp^3^) [965]. Examples of P–C (sp^3^) cleavage are rare, but the unprecedented cleavage of both of the P–CH_2_ bonds in an η-alkylidene iron complex has recently been reported [966]. Thus, treatment of [Fe_2_(CO)_6_(η-CO)(η-Ph_2_PCH_2_PPh_2_) with ethyl diazoacetate under UV irradiation gives [Fe_2_(CO)_6_(η-CHCO_2_Et)(η-Ph_2_PCH_2_PPh_2_), which, on heating, undergoes P–CH_2_ bond cleavage and C–C bond formation, affording CH_2_ = CH(CO_2_Et) and [Fe_2_(CO)_6_(η-PPh_2_)_2_].

Transition-metal oxophilicity [967] and steric factors [968] have emerged as crucial parameters that determine the relative stability of mono- and bi-dentate acyl coordination modes, and these factors have recently been examined in relation to a solution structure of the (PPh_2_Me)_2_Fe(CO)η^2^-C(O)Me)I complex, in which there was found facile alkyl ↔ η-acyl equilibrium [969]. Activations of H–X bonds by transition-metal complexes are key steps in many catalytic functionalizations of C–C multiple bonds. Accordingly, N–H bond activation by metals [970] may play a key role in some catalytic alkene hydroamination [971] pathways. The facile activation of amide (RCONH_2_)N–H bonds by iron phosphine complexes, e.g., *cis*-FeH_2_ (dmpe)_2_ (dmpe) = 1,2-bis(dimethylphosphino)ethane, and FeH(C_6_H_4_PPhCH_2_CH_2_PPh_2_)(dppe)(dppe = 1,2-bis (diphenylphosphino)ethane, has recently been described [972].

#### 9.10.3 Novel Charge-Transfer Complexes and Ferromagnets

Donor-acceptor (DA) interactions in molecular solids provide to a large extent the foundation of the extensive interest in these materials [973,974]. The present goal has been the better to understand structure-function relationships in these materials, which may result in new concepts for rational modification of structural and electronic properties that are relevant to possible electronic applications [975]. The behavior of the organometallic DA solids recently studied [976] demonstrated that the design of donor-acceptor complexes need not invoke components having planar molecular structures, as face-to-face stacking of π-networks is not required for charge-transfer behavior.

One-dimensional (1-D) charge-transfer complexes frequently exhibit unusual optical, electrical [973], and, as recently discovered, unusual cooperative magnetic properties [977]. For example, the reaction of decamethylferrocene, Fe(C_5_Me_5_)_2_, and 7,7,8,8-tetracyano-*p*-quinodimethane, TCNQ, gives three major products of various stoichiometry, conductivity, and magnetism [978]. Replacement of [TCNQ]^·−^ by [TCNE]^·+^ (TCNE^·−^ = tetracyanoethylene) led to the similarly structured decamethylferrocenium tetracyanoethenide [Fe(III)(C_5_Me_5_)_2_]^·+^ [TCNE]^·−^, which has been characterized as being a bulk ferromagnet [979]. The search for ferromagnetic organic compounds and polymers, however, maintains academic interest [980]. Recently, a novel, crystalline, ferromagnetic, inorganic-organic host-guest complex has been synthesized by a procedure wherein magnetite (Fe_3_O^4^) was sequestered by bis(pyridoxylidenehydrazino)phthalazine at ambient temperature and neutral pH [981].

#### 9.10.4 Recent Physical, Spectral, and Catalytic Studies of Organoiron Complexes

Few iron complexes have been found to be efficient hydrogenation catalysts [982]. However, a homogeneous iron(II) system that brings about the selective reduction of terminal alkynes to alkenes has recently been reported [983]. Thus, terminal alkynes are selectively hydrogenated to alkenes by the iron(II) catalyst precursors [(PP_3_)FeH(N_2_)BPh_4_] and [(PP_3_)FeH(H_2_)BPh_4_] in tetrahydrofuran at 1 atm of H_2_ at 20 to 66 °C [(PP_3_=P(CH_2_CH_2_PPh_3_)_3_].

As determined by mass spectrometry, for nitriles containing four to seven carbon atoms, the “anchored” transition-metal ion Fe^+^ exclusively activates the C–H bond of the terminal methyl group by oxidative addition [984]. Other areas of interest include mechanism in the hydrometalation and hydrogenation reactions of (η^5^-C_5_H_5_)Fe(CO)_2_H with conjugated dienes [985], conformational studies of five-membered chelate rings in iron(III) complexes [986], reactions of hydroxyl radical with 2,2′-bipyridyl iron(II) complexes [987], and dynamics of spin-state interconversion of iron(III) complexes in solution as a function of the pressure [988]; characterization of the first stable iron-methylene complex ${\left[\left(\eta^{5}-C_{5}M_{5}\right)\text{Fe}\left(\text{dppe}\right)\left(={\text{CH}}_{2}\right)\right]}^{+}{\text{BF}}_{4}^{-}\left(\text{dppe}=p,p^{\prime }-{\text{Ph}}_{2}{\text{PCH}}_{2}{\text{CH}}_{2}\text{PP}\right)$ [989], the structure of iron(II) molecular hydrogen complexes containing monodentate phosphine ligand [990], characterization of ethyl oxalyl tetracarbonyl iron anion [(CO)_4_FeCOCO_2_Et]^−^ [991], structure of a triangular cluster [Fe_2_(CO)_8_(η-AuPPh_3_)] [992], structural characterization of iron(III) complexes of chlorin and isobacteriochlorin macrocycles [993], the first fully characterized hexaminoiron(III) complex [994], and the molecular structure of η^3^-Bi[Fe(η^5^-C_5_H_5_)(CO)]_3_ [995]. Other recent interests encompass the carbon-13 NMR spectrum of solid iron pentacarbonyl [996], axial-equatorial exchange of carbonyl groups in [HFe(CO)_4_] anion in the solid state as determined by carbon-13 NMR spectroscopy [997], the charge distribution in bimetallic organoiron complexes by ^57^Fe Mössbauer spectroscopy [998], electrical conductivities of *s*-tetrazine-iron complexes [999], and intramolecular Kharasch cyclizations of the alkenic trichloromethyl substrates in the presence of the FeCl_2_[P(OEt)_3_]_3_ catalyst, to afford five- or six-membered-ring exo-closure products, e.g., trichlorocyclopentane or trichlorocyclohexane derivatives [1000].

Additional pertinent, recent references include: a new book on organometallic chemistry of transition elements [1001], spectroscopic studies of the mixed-valent [Fe(II), Fe(III)] forms of the non-heme iron protein hemerythrin [1002], a survey of tetraphenylporphyrins and metallotetraphenylporphyrins [1003], carbon monoxide insertion into iron-carbon bonds of σ-alkyl porphyrins [1004], and the gas-phase chemistry between Fe^+^-benzyne and alkenes [1005].

The solubilization and mediation of iron uptake by aerobic microbes occurs via the production of low molecular weight ligands that have very high affinities for ferric ion [1006,1007]. It has been shown that the microbial transport process generally involves a recognition of the geometry and chirality at the metal center of the coordination complex (siderophore). The characterization of the coordination chemistry of the siderophores and the mechanisms by which they deliver iron to the microbial cell is the topic of a recent study [1008]. Iron uptake and inhibition studies with a number of siderophores, semisynthetic siderophores, and synthetic siderophore analogues have demonstrated remarkable stereospecificity and enantioselectivity of the microbial receptors [1006,1007].

### 9.11 Addenda to Addenda

Selected work from the most recent literature on organoiron complexes is presented next.

#### 9.11.1 New Carbon-Carbon Bond-forming Reactions

In a series of diiron complexes, the diiron methylidene complex ${\left[C_{5}H_{5}\left(\text{CO}\right)\text{Fe}\right]}_{2}\left(\eta -\text{CO}\right){\left(\eta -\text{CH}\right)}^{+}{\text{PF}}_{6}^{-}$ is interesting in its relation to CH groups bound to metal surfaces. It is also an important intermediate in the synthesis of a wide range of hydrocarbyl-bridged diiron compounds [1009]; it adds its CH bond across the carbon-carbon double bond of simple alkenes in a hydrocarbation reaction, to produce a new series of (η-alkylidyne) diiron complexes [1010]. Because of the central importance of these diiron methylidene complexes to the new chemistry of η-alkylidene, η-acylium, and η-nitrilium complexes, the mechanism of its formation has been studied in detail [1011].

Dinuclear iron η-acetylide complexes, e.g., [FP_2_(C≡C)-R]BF_4_ (R = H,Ph); FP = (η^5^-C_5_Me_5_)Fe(CO)_2_ [1012], in which the acetylide ligand is bound to one metal center in a η^1^ fashion (π-bonded) and to the other metal center in a η^2^ fashion (π-bonded), have attracted much attention as a model for surface-bound species. Oscillation of the bridging acetylide ligands between two metal centers in a manner reminiscent of a windshield wiper often results in fluxional behavior, and this is observed for the diiron acetylide complex.

Interest in metal-bound vinylketenes as reaction intermediates [1013–1015] led to facile preparation of (vinylketene)tricarbonyliron(O) complexes [1016], and the synthesis of (vinylketeneimine)tricarbonyliron(O) complexes [1017].

Phosphaacetylenes have been successfully cyclooligomerized in the presence of suitable metal complexes [1018,1019]. The degree of cyclooligomerization, and the structure of the resulting metal complexes, seem to depend mainly upon the nature of the metal used. An example of a novel cyclodimerization of phosphaalkyne (e.g., 3,3-dimethyl-1-phospha-1-butyne) in the presence of nonacarbonyliron, Fe(CO)_9_, at 100 °C (to give 2,4-di-*tert*-butyl-tricarbonyl-1,3-diphosphacyclobutadineiron, 63% yield) has been reported [1020].

Electrophilic carbene complexes [M=CHR]^+^ are key intermediates in single and double carbon-carbon bond formation [66,1021,1022]. The classical route to carbene complexes [M = CHR]^+^[M = Fe,Cr] involves the ionization of α-alkoxyalkyl derivatives, M–CH(OR′)R which are generally prepared from alkoxycarbene complexes by hydride reduction or by using organolithium reagents [1023]. Thus, the bimetallic complex [(η^5^-C_5_Me_5_)Fe(CO)_2_{η-η^1^,η^6^-CH(OMe)CH_2_C_6_Me_5_}Fe(η-C_5_H_5_)][PF_6_], which is readily prepared by an electrophilic carbene-alkene coupling reaction, is specifically converted into either pentamethyl-η^6^-vinyl benzene or a related dinuclear (η-η^1^-η^6^-vinylarene) complex. This procedure offers access to new π- and σ,π-vinylarene complexes (e.g., single and double carbon-carbon bond formation, respectively) via iron-carbene intermediates [1024].

Syntheses involving the use of metal atoms on a preparative scale are of growing interest. The starting point of some new strategies is the generation of highly reactive intermediates, from, for example, atomic iron and arenes. They then can be transformed into clusters, π complexes, organoboron and phosphorus cages, and organic cycloadducts [1025].

In the photochemically generated tricarbonyliron complex of 9-fluorenylidene (tetramethylpiperidine)borane, the metal complex fragment is bonded in an η^4^ fashion to a *cis*-borabutadiene unit, thereby strongly perturbing the aromaticity of one benzene ring. Consequently, the η^4^ complex reacts readily with two-electron donors, to afford η^2^ complexes [1026].

Additional carbon-carbon bond formations are: synthesis of [Fe_2_(CO)_6_(η-C(OR)H)(η-CR′ = CR′H)]^−^ anions (R = Me, Et; R′ = Ph, H) (at a dinculear iron center) [1027], synthesis of a new *tert*-butylnitroso complex [CpFe(CO)(PPh_3_)N(O) *tert*-Bu]^+^ (Cp=η^5^-C_5_H_5_) [1028], synthesis of a new bis(phosphino)methanideiron complex Cp(CO)Fe(Ph_2_PCH PPh_2_) [1029]; also preparation of derivatives from doubly-bridged diiron complexes of the type [(η-η^2^-ROC(1)S)Fe_2_(CO)_6_(η-SMe)] (via ligand-exchange reactions) [1030], synthesis of cycloheptatriene complexes (η^3^-C_7_H_7_)Fe(CO)_3_EPh_3_ (E = Sn, Pb) [1031], and synthesis of benzophenones through carbonylation of aryl iodides catalyzed by the Fe(CO)_5_-CO_2_(CO)_8_ system [1032].

#### 9.11.2 Stereoselectivity and Asymmetric Synthesis

Cyclic and acyclic (η^4^-polyene)Fe(CO)_3_ complexes continue to attract attention as intermediates, particularly for asymmetric syntheses [31,122]. For such η^4^-triene complexes as (tropone)- or (cycloheptatriene)-Fe(CO)_3_, interest has centered particularly on the regio- and stereoselectivity of reactions at the uncoordinated double bond [20–21]. (Tropone)Fe(CO)_2_(PR_3_) complexes are attractive candidates due to their enhanced reactivity toward electrophiles [1033] or regiospecificity of the reactions of the derived dienyl salts with nucleophiles [1034]; furthermore, there is a possibility of using a chiral phosphine as a center of induction and resolution [1035]. The synthesis, structure, reactivity, and diastereoisomer separation of (tropone) Fe(CO)_2_L complexes (L=PR_3_, (+)-neomenthyldiphenylphosphine) have been described. Here, the changes in reactivity induced by phosphine substitution in (tropone)Fe(CO)_3_, and the application of (+)-neomenthyldiphenylphosphine as a new resolving center for this chiral complex, are highlighted [1036].

In the development of synthetic methodology that preferentially leads to the formation of a single enantiomer of a targeted chiral compound, of particular importance are carbon-carbon bond-forming reactions whose diastereo- and enantioselectivity are derived through the use of catalytic quantities of chiral transition-metal catalysts [41,257,279,283,464, 1037–1039]. For example, an elegant synthesis of oxazolines utilizing a gold(I)-catalyzed aldol reaction in the presence of chiral ferrocenylamine ligands that possess both planar and central chirality has been reported [466,467,470]. The mechanistic aspects of this interesting, carbon-carbon bond-forming reaction, using chiral ferrocenylamines (the Hayashi-Ito catalyst) have been studied in considerable detail [468,1040]. The stereochemistry of the metallocenes, and especially of ferrocene derivatives, has been a topic of considerable interest for many years [41,546]; the ferrocene derivatives have also been used as chiral ligands in asymmetric, catalytic processes [464,1041]. Diastereo-isomeric 1,2,3-trisubstituted ferrocenes have been synthesized via introduction of sulfur and phosphorus substituents into the ferrocene nucleus in chiral 1-(*N*,*N*-dimethylamino)ethylferrocene by stereoselective lithiation and reaction with electrophiles [1042]. An asymmetric synthesis took a big step forward because of the discovery of a cheap method for catalytic chiral epoxidation of unsubstituted alkenes. The new reaction creates two dissymmetric carbon atoms in one step [1043].

The ability of esterases and lipases to produce optically active alcohols by the kinetic resolution of racemic esters is well established [1044]. A new approach to the en2ymatic resolution of racemates via stereoselective transformations catalyzed by enzymes in organic solvents constitutes a new chapter in modern synthetic methodology [1044–1050]. The enantioselective esterification of chiral alcohols using lipases in organic solvents [1047], and inter-esterification reactions involving chiral acyl units and achiral alcohols, have been described [1048]. A number of racemic primary and secondary alcohols were successfully resolved following acylation (e.g., transesterification) by using *Pseudomonas* lipase as an asymmetric catalyst in organic solvents [1045]. An indicative example is the enzymatic resolution of 1-ferrocenylethanol. This organometallic compound is decomposed by water, thus making the conventional methodology of resolution in aqueous media impossible; in contrast, enzymatic transesterification in benzene [1051] or *tert*-butyl methyl ether [1052] results in facile resolution.

#### 9.11.3 Ferrocene and Ferrocene Derivatives

During this decade, there has been considerable interest in electron-transfer, chain-catalyzed organometallic reactions, i.e., reactions of organometallic complexes catalyzed by electrons or electron holes [1053–1055]. The intimate mechanism of the two-electron transfer (ET) process has attracted the interest of theoreticians and experimentalists for many years [1056–1057]. Two ET systems are useful redox mediators for energy conversion devices that require multi-electron steps. For example, the x-ray crystal structure of the model mixed-arene ferrocene ${\left[{\left({\text{FeC}}_{5}{\text{Me}}_{5}\right)}_{2}\left(\eta^{2},\eta^{10}-\text{biphenyl}\right)\right]}^{+}{\text{PF}}_{6}^{-}$ showed a 37-electron configuration, indicating that two-electron transfer proceeded with structural reorganization and stabilization in the second electron-transfer [1058]. This supplements a study [1059] on electron-transfer chelation of dithiocarbamate complexes, e.g., [Fe(η^5^-C_5_R_5_)(η^1^-SC(S)NMe_2_) (CO_2_)](R = H, Me) catalyzed by ferrocenium salts [Fe(Cp_2_)]^+^(PF_6_)^−^. Another related study involving ferrocenes reports [1060] that, although most carbanions react with ${\left[\text{Fe}{\left(\eta^{6}-C_{6}{\text{Me}}_{6}\right)}_{2}\right]}^{2+}{\left({\text{PF}}_{6}^{-}\right)}_{2}$ by electron-transfer without C–C bond formation, functionalization of the mesitylene (C_6_Me_6_) ligand can proceed via protection by a hydride; in this fashion, activation of a single aromatic ligand in a complex can be achieved.

A large number of interesting, cage hydrocarbons have been synthesized, and some attempts have been made to entrap a metal ion or a small neutral atom into the cavity of such cage hydrocarbons. A series of macrocyclic ferrocenes as molecular pivots has been developed, including superferrocenophane [1061], ferrocene-cryptands [1062], and inclusion of ferrocene in β- or γ-cyclodextrin (cyclodextrin host-guest complexes) [1063]. A review of macrocyclic, receptor molecules includes a discussion on ferrocenyl ionophores and ferrocene-cryptand complexes [900]. An interest in macrocyclic (host) ferrocenes continues, with the synthesis of ferrocene-dicoronands, as possible biological regulators (e.g., mimicking of allosteric interactions) [1064].

A series of decaphenylmetallocenes has been extended by the synthesis and characterization of decaphenylferrocene having a novel, zwitterionic structure; the compound is readily protonated, to give [Fe(η^5^-C_5_Ph_5_){(η^6^-C_6_H_5_)HC_5_Ph_4_}]^+^X^−^ [1065]. Additional syntheses of ferrocenes include the synthesis of ferrocene-containing carbamates [1066], of 2,3-dimethylindole-ferrocenes [1067], and of cynichrodenylferrocenyl carbinol [1068]; also, of isocyanoferrocenes [1069], of azaferrocenes [1070], of 1,1-bis(tributylstannyl)ferrocene [1071], of ring-substituted ferrocenes [1072], and possibly, of a phosphorus analogue of ferrocene [1073].

#### 9.11.4 Porphyrins and Cytochrome Enzymes

The family of heme-containing enzymes called cytochrome P-450 are important in the degradation of xenobiotic agents and in the biosynthesis of steroids, and the mechanism of their inactivation has been an area of extensive research [1074–1076]. The many oxidative transformations performed by cytochrome P-450 on substrates can be classified into four main types: alkane and arene hydroxylation, heteroatom oxidation, dealkylation of heteroatoms, and alkene epoxidation [1076,1077]. Cytochrome P-450 enzymes, comprising iron porphyrins, constitute an extraordinarily versatile class of biological oxidation catalysts [556,1078,1079]. In their function as monooxygenases, they are responsible for the metabolism of endogenous, as well as exogenous, lipophilic substrates. For monooxygenase activity, molecular oxygen is bound by the heme iron(II). In a sequence of redox processes, the bound *oxygen* is cleaved, with formation of water and a high-valent oxoiron complex, believed to be Porph^·+^ Fe(IV) = O (a cation-radical). This oxoiron complex subsequently transfers its oxygen atom to the complexed substrate. A porphyrin-bridged cyclophane complex has been prepared in order to mimic monooxygenase activity and to serve as a supramolecular catalyst for the hydroxylation of polycyclic arenes. In methanol, such arenes as anthracene, acenaphthylene, and phenanthrene are firmly complexed in the non-polar interior of a cyclophane complex (M = 2H). In 2,2,2-trifluoroethanol in the presence of iodobenzene, an iron(III)-cyclophane complex (M = FeBr) catalyzes the oxidation of acenaphthylene to acenaphthen-1-one (65% yield) [1080].

In regard to references [553 and 556], a useful addition concerning cytochrome C is in order. Cytochrome C, a component of the respiratory chain, is an electron-transfer heme protein having a molecular weight of 12,500; the topic has been reviewed [1081], and recently discussed [1082, 1082a]. Cytochrome C exhibits a visible spectrum characteristic of heme-containing proteins (the Soret absorbance maximum is at 410 nm; *E* = 1.05 mol^−1^cm^−1^). This protein is unusual, in that the heme prosthetic group is covalently attached to the protein backbone via thioether linkages involving cysteine residues, whereas histidine serves as an axial ligand to the ferric ion. Possible pathways for electron transfer from histidines 33 and 39 to the heme in cytochrome C have been discussed [1082, 1082a].

Photodynamic therapy is an experimental cancer treatment modality that effectively destroys cancer cells by interaction of light with a photosensitizing dye, presumably to form singlet oxygen [1083]. Some porphyrins have been shown to be particularly effective sensitizers in this regard. Several ether-ester linked oligomers containing between two and six hematoporphyrin units have been found to be biologically active and to show significant tumorcidal activity [1084,1085]. This study has now been extended to include some deuteroporphyrins, particularly the synthesis and use of regioselective methyl- and vinyl-deuterated hemins [1086].

Heme proteins participate in a diverse array of biochemical phenomena, including oxygen transport (hemoglobin) and oxygen activation (cytochrome P-450 enzymes). Recently, the self-assembly of a new porphyrin complex has been accomplished [1087,1088]; this novel analog of heme proteins has a hydrophobic groove that is a potential site for substrates. Synthesis of the complex, a template-driven, self-assembly process propelled by noncovalent interaction, begins with formation of an inclusion complex between a tetraaminoporphyrin and two cylindrical, O-methyl-substituted cyclodextrin species. Upon addition of sodium tetraphenylboron, a supramolecular complex forms that has eleven separate units: one porphyrin, two encapsulating cyclodextrins, two sodium ions, and six tetraphenylborones situated about the periphery. A hydrophobic groove between the cyclodextrins, lined with fourteen methyl groups, circumscribes the metal-binding site of the porphyrin. Replacement of the cyclodextrin moieties with other hosts may be useful in controlling substrate specificity (e.g., the size, shape, and hydrophobicity of the groove).

Chemists have designed a synthetic, five-part molecule that undergoes multistep electron-transfer to convert the energy of light into chemical potential, as in photosynthesis [1089]. The pentad consists of a carotenoid polyene (C), two porphyrin moieties (P), and two quinone groups (Q) linked linearly (C–P–P–P–Q–Q). The excitation of one form of the pentad by light yields the charge-separated state [C^·+^–P–P–Q–Q^·−^], having a lifetime of 55 μs. Irradiation of another form of the pentad gives a similar charge-separated state having a longer lifetime (340 μs). More than half of the initial excitation energy is preserved in the charge-separated states. Thus, it appears that there is no *a priori* reason why the essential features of photosynthetic energy conversion cannot ultimately be reproduced successfully by use of man-made molecular devices.

Substrate activation by cytochrome P-450, and monosubstituted hydrazine reactions with myoglobin and hemoglobin, have revealed the formation of a-bonded alkyl- or aryl-iron(III) porphyrin complexes [556,1090]. The potential biochemical relevance of organometallic complexes has led in large part to the synthesis of σ-bonded alkyl- or aryl-iron(III) porphyrins. There are three primary routes for synthesis of the alkyl- or aryl-iron(III) porphyrins; these are (1) reaction of iron(III) porphyrins with a source of carbanions, (2) combination of an alkyl or aryl radical with an iron(II) porphyrin, and (3) reaction of iron(I) porphyrin anions with a source of carbocations, typically alkyl or aryl halides. The synthesis and characterization of the first paramagnetic alkyl- or aryl-iron(III) porphyrin complexes has been described [1091].

A variety of one-side-hindered iron porphyrins has been synthesized and studied [905–909]. The discrimination of binding between carbon monoxide and oxygen in model hemes may lead to a straightforward explanation of the natural regulatory mechanism. So far, there is only one demonstrated example of a model compound that exhibits a tilt of the CO relative to the heme that is normal in the solid state of the pocket porphyrin [1092]. In the series of hybrid, basket-handle porphyrins, the effect of a decrease in the length of the aliphatic chain spanning one side of the porphyrin is not to increase the degree of the tilt of the bound carbonyl but rather to increase the degree of ruffing of the porphyrin core [1093]. As a ^57^Fe NMR study shows [1094], in the hybrid, basket-handle porphyrins, the ruffing leads to large changes in the iron-*d*-orbital energies that may be important in understanding ligand binding in heme proteins and models. Studies on porphyrins include the synthesis of S/N, S/O mixed ligand complexes of iron(III) tetramesityl-porphyrin [1095], and synthetic and structural studies of a sapphyrin, a 22-π-electron pentapyrrolic “expanded porphyrin” [1096]; also, insertion of iron into porphyrins [1097], gable porphyrin metal complex as a double recognition model [1098], and fixation of amino acids with bifunctional metalloporphyrin receptor [1099]. Molecular recognition by chromoionophores and porphyrins is a new chapter in the chemistry of macro-molecules. Molecular recognition, a “magic” expression in modern organic chemistry, circumscribes the already well known, and, for biochemical processes, particularly important phenomenon that certain molecular structures match and interact with each other. An example is the agonist-receptor interaction. The spectrum of receptor model compounds has been extended rapidly in the past few years, for example, by cleft structures, which are readily accessible by reaction of the Kemp tricarboxylic acid with benzene-1,3-diamine (or its dimethyl derivative). Derivatives of the Kemp triacid are useful as scaffolds for molecular recognition and as probes for stereoelectronic effects at carboxyl oxygen [1099a].

Other studies on iron porphyrins, including synthesis and spectral characterization, are iron(III) porphyrin-promoted aerobic oxidation of sulfur dioxide [1100], fluoride ion-promoted oxidative cleavage of (η-oxo)iron(III) tetraarylporphyrins [1101], electrochemical and spectral characterization of stable iron(IV)tetrakis-5,10,15,20-(*N*-methyl-4-pyridyl)porphyrin in aqueous solution at room temperature [1102], reaction of dioxygen with paramagnetic (S = l/2) tetraarylporphyrin Fe(III) CH_2_CH_2_ dianion [1103,1104]; electrochemical characterization of intermolecular nitrosyl transfer between iron and cobalt porphyrins [1105], theoretical study of the electron density in iron(II) porphyrin bis(water) [1106], binding sites for nitrite binding in iron(III) porphyrinates [1107], instability of the nitrite-iron(III) porphyrinate system [1108]; synthesis and characterization of low-spin bis(imidazole) (tetraphenyl-porphyrinato)iron(II) complexes [1109], synthesis of five-coordinate mercaptoiron(II) porphyrins [1110], x-ray absorption spectral study of ferric, high-spin hemoproteins [1111], synthesis and optical properties of conformationally constrained trimeric and pentameric porphyrin arrays [1112], and related porphyrin pyrrole sequencing by mass spectrometry [1113].

#### 9.11.5 Biological and Biochemical Applications

Proteins and nucleic acids carry out specific functions, such as molecular recognition, information transfer, and catalysis, which depend in detail on the structure of each macromolecule. Cleavage of DNA or transfer RNA by metal chelates is an important new approach to characterizing structural features of nucleic acids and their complexes in solution [1114–1118], because chain scission by reactive oxygen species occurs close to where the redox-active metal complex is bound. Likewise, cleavage of a protein by metal complexes bound at a particular site could give information about the folding of the polypeptide chain [1119], or help in deducing the major secondary, and even tertiary, structure features in DNA and tRNA molecules [1117].

Specific cleavage of a protein has been achieved by introducing a metal-binding site at one position in a polypeptide chain. Thus, the iron chelate [Fe(II)-1-(p-bromoacetamido)-EDTA] attached to a residue on the protein bovine serum albumin was treated with H_2_O_2_ *L*-ascorbate at pH 7.0 and 25 °C, to give three peptide fragments which together account for the entire polypeptide chain [1118]. Cleavage of DNA or tRNA by the Fe(EDTA)^2−^ chelate is apparently mediated by hydroxyl radicals [1116,1120], in contrast to the action of other transition-metal complexes [1118].

Interaction of bleomycin (an antitumor antibiotic), Fe(II), and limiting oxygen, or bleomycin, Fe(III), and H_2_O_2_ with the hexamer d(CGCGCG) (a hexa-nucleotide) results in the production of an oxidatively damaged sugar lesion, 2-deoxy-4-pentulose, that is produced stoichiometrically with respect to free nucleic acid base release. The implications of these results with respect to the mechanism of nucleic base release in bleomycin-mediated DNA degradation have been presented [1121].

Isopenicillin N synthase (IPNS) is a non-heme, Fe(II)-dependent enzyme found in β-lactam antibiotic-producing microorganisms that catalyzes the formation of isopenicillin N from δ-(L-α-aminoadipoyl)-L-cysteinyl-D-valine (ACV) [1122]. Unlike the reactions catalyzed by the Fe(II)-containing dioxygenases, which incorporate the elements of dioxygen into their substrates [1123], the two oxidative ring closures of ACV, forming β-lactam and thiazolidine rings catalyzed by IPNS, result in the complete four-electron reduction of 1 equivalent of dioxygen to 2 equivalents of water [1122]. Here, spectroscopic evidence for the metal-binding site of Fe-IPNS, and the nature of its endogenous ligands, was presented [1124].

Transferrins are globular proteins (*M*_r_=80,000) acting as physiological iron carriers involved in the biosynthesis of hemoglobin and other iron-containing proteins [1125]. These double-site proteins show remarkable metal-binding properties that have led to the characterization of several metal-transferrin derivatives, both tripositive and dipositive metal ions [1126].

Nickel as a trace element in biology occurs in several enzymes; for example, [NiFe)-hydrogenase enzymes contain mononuclear nickel sites and Fe–S clusters [1127–1128], and, in a [NiFeSe]-hydrogenase, a selenocysteinate residue is bound to nickel [1128]. Work related to [NiFe]-hydrogenases has been described [1129].

Self-assembly is a process by which organized supramolecular structures are spontaneously generated from their component molecular parts in high yield and specificity. Prominent biological examples [1130] are double-helix formation of nucleic acids [1131] and self-assembly of viral protein coats, or highly organized supramolecular entities (for example, membranes, ribosomes, and multicomponent enzyme complexes) from relatively simple subunits [1132], In addition, it was proposed the self-assembly phenomenon might have been an essential component in the molecular events that comprise protobiogenesis [1130].

A new synthesis of the singly-bridged double cubane cluster [(Fe_4_S_4_Cl_3_)_2_S]^4−^ tetraanion, involving coupling of two simple unfunctionized [Fe_4_S_4_Cl_4_)^2−^ dianions has been reported [1133]. This cubane cluster cannot, however, be prepared by well developed “spontaneous self-assembly reactions” [1134–1136], and thus its elaboration must be executed by use of a carefully controlled coupling reaction. Many other “spontaneous self-assembly reactions” that include formation of polynuclear metal complexes, multidentate ligands, and bridging ligands are well known; the topic has been discussed [1137] and reviewed [1138–1140].

#### 9.11.6 Miscellaneous Recent Results

New additions to the chemistry of organoiron complexes are the following: reaction of [(η^5^-C_5_H_5_)(CO)Fe{η-C(CF_3_) = C(CF_3_)SMe}_2_Fe(CO)(η-C_5_H_5_)] with [Fe_3_(CO)_12_] (to yield di- and tri-nuclear iron-alkyne complexes) [1141], synthesis of FeCH(SR)COC(CO_2_Me) = C(CO_2_Me)S(CO)(PR_3_)_2_ complexes [1142], synthesis of the novel –η^2^-carbido complex [(TPP) Fe = C=Re(CO)_4_Re(CO)_5_] [1143], and synthesis of (η^5^-C_5_H_5_)Fe(L)S_2_CR(L=Ph_2_PCH_2_PPh_2_; R = Me, Ph) [1144], a new chemistry of iron-methylene complexes ${\left[\text{Fe}\left(\eta^{5}-C_{5}{\text{Me}}_{5}\right)\left(\text{CO}\right)\left(L\right)\left(={\text{CH}}_{2}\right)\right]}^{+}{\text{BF}}_{4}^{-}\;\left(L=\text{CO},{\text{Ph}}_{3}\right)$ [1145], Friedel-Craft acetylation of (CO)_2_(NO)(η^5^-C_5_H_4_)CH_2_(η^5^-C_5_H_4_)Fe(η^5^-C_5_H_5_) [1146], reaction of di-*tert*-butylcyclopentadienyllithium Li[η^5^-C_5_H_3_(C Me_3_)_2_-1,3] with FeCl_2_ [1147], reaction of the dianion [(η-Te)_2_Fe_2_(CO)_6_]^2−^ with metal halides [1148], reaction of α,β-unsaturated aldehydes complexed at the C = C bond with Fe(CO)_3_L units (L=CO, L = P(OMe)_3_), with stabilized phosphoranes [1149]; also synthesis of [Fe(diars)(Lα)(CO)_2_(CH_3_)]^+^ (diars=*o*-phenylenebis(di-methylarsine) [1150], synthesis of bis(iron)η^1^-O:η^1^-O+) formate complexes, and their heterobimetallic analogs [1151], iron-promoted nitrene-extrusion reactions in 7-azanorbornadiene derivatives [1152], and regio- and diastereoselectivity in the rearrangement of cationic iron(II) η-1-(1-methylcycloalkyl)methylidenes [1153].

Other work included the synthesis of heterobimetallic Fe(III)-Mn(II) complex (containing a heptadentate polyimidazole ligand) [1154], formation of iron thiolate complexes, for example, FeH(RS)(DMPE)_2_, and Fe(RS)_2_(DMPE = 1, 2 [DMPE = l,2-bis(dimethylphosphino)enthane] [R = Ph-, CH_3_CH_2_-, (CH_3_)_3_C-] [1155], photolysis of alkoxy-substituted disilanyliron complexes, e.g., CpFe(CO)_2_SiMe_2_Me(OM_e_)_2_(Cp = η^5^-C_5_H_5_) [to afford novel, donor-stabilized bis(silylene)iron complexes] [1156], and thermal- and light-induced spin transition in the iron(II) spin-crossover complex [Fe(1-methyl-1H-tetrazole)_6_] (BF_4_)_2_ [1157]. Other selected topics include synthesis of the chiral cluster Fe_3_(CO)_9_[η^3^-B(H)C(H)CMe)](B = boracyclopropene) [1158], iron carbonyl complexes of isobenzofulvene [1159], iron-trichloride-phosphine adducts [1160], and protonation of FeP(CH_2_CH_2_CH_2_PMe_2_)_3_H_2_ with alcohols [1161]; preparation of the complex (NEt_4_)[Fe_2_(CO)_8_(η-AuPh_3_)] [1162], preparation of Fe_2_^+^-benzyne ion in the gas phase [1163], rearrangements of cyclopropanes σ-bonded to iron [1164], iron-catalyzed oxidation of bis(imidazol-2-yl)methane [1165], and synthesis and characterization of a new T-form hemoglobin model [1166].

Other studies reported on the resonance Raman spectra of iron(II) porphyrins [1167], electronic and steric effects in Fe(CO)_2_L(η^4^-benzylidene-acetone) [1168], the crystal structure of Fe_3_(CO)_10_(CNBu^t^)_2_ [1169], the crystal structure of the dimer {K_2_Fe(C_2_O_2_S_2_)_2_NO·H_2_O}_2_ [1170], and an ESR study of the spin-cross over complex [Fe(2-aminoethyl)pyridine]Cl_2_·C_2_H_5_OH [1171]; the dynamics of molecular hydrogen in the complex *trans*-[Fe(η^2^-H_2_)(H)(PPh_2_CH_2_-CH_2_PPh_2_)_2_]BF_4_ (in the solid state) [1172], rotation of the cyclopentadienyl ligand in the Fe–Fe complex (η-CO_2_)[FeCp(CO)]_2_ (*C*_p_ = ca 70% η^5^-C_5_H_5_) (in the solid state) [1173], the ESR spectrum of the Fe_2_(CO)_8_^−^ radical [1174], reaction of hydroxy radicals with Fe(II) complexes, e.g., [Fe(2,2′-bipyridyl)_3_]^2+^ and [Fe(2,2′-bipyridyl)_2_(CN)_2_] [1175], and the x-ray crystal structure of iron dithiocarbamates, e.g., Fe(η^5^-C_5_Me_5_)(η^1^-SC(S)NMe_2_)(CO)_2_ and Fe(η^5^-C_5_Me_5_)(η^2^-S_2_CNMe_2_)(PPh_3_) [1176]. Further structural studies and reactions included a structural study of disilanes bridging two [(η^5^-C_5_H*_n_*)Fe(CO)_2_] groups (*n* =4,5) via both iron atoms or via both cyclopentadienyl ligands [1177], the crystal structure of the heteroatomic trinuclear cluster Fe_2_Os(CO)_12_ [1178], the binding and activation of halocarbons by iron(II) complexes; the halocarbon coordinated via σ-donation of a halogen lone pair with retention of their carbon-halogen bonds [1179]; electron mobility in electroactive polymer films of iron complexes of the type [M(v-bpy)*_n_* (L)_m_] (M = Fe, Ru; v-bpy=4-vinyl-4′-methyl-2,2′-bipyridine; L=CN) [1180], iron(III)- induced cleavage of cyclic allylic hydroperoxides to dicarbonyl compounds [1181], and site-specific cleavage of proteins using the iron chelate of ethylenediaminetetraacetic acid (EDTA) [1182,1183].

#### 9.11.7 Additional Miscellaneous Results

Some pertinent reviews include: reactions of carbon dioxide catalyzed by transition-metal complexes [1184], oxo- and hydroxo-bridged diiron complexes in regard to a chemical perspective on a biological unit [1185], a classification of organometallic complexes [1186], and non-enzymatic, asymmetric transformations involving symmetrical, bifunctional compounds [1187].

Some pertinent books are concerned with the following topics: organometallic chemistry [1188], carbon dioxide activation by metal complexes [1189], carbyne complexes [1190], structure and reactivity [1191]; organometallic radical processes [1192], mechanisms of inorganic and organometallic reactions [1193], and spectroscopic properties of inorganic and organometallic compounds [1194].

Some pertinent books on biotransformations treat the following topics: enzyme catalysis processes [1195], dynamics of proteins and nucleic acids [1196], enzymes as catalysts in organic synthesis [1197], biotransformations in preparative organic chemistry [1198], biomineralization [1199], and bioseparations [1200]; a dictionary of biochemistry and molecular biology [1201], and metalloproteins, their chemical properties and biological effects [1202].

Other selected topics are summarized next. A study [1203] may serve to supplement an earlier report [1089] in providing a better understanding of the mechanism of electron transfer in photosynthesis. The fluorescence found in certain neutral porphyrin dimer complexes may help clarify the nature of the lowest excited state of the bacteriochlorophyll “special pair,” the primary electron donor in the photosynthetic reaction center [1203].

Other pertinent studies included hydrogen-atom transfer vs electron transfer in iron(III) porphyrin catalyzed benzylic oxidations [1204], electrocatalytic hydroxylation of alkanes and identification of a fluoroiron(V) porphyrin intermediate [1205], and modulation of interprotein electron-transfer energetics by heme-ligand variation [1206].

Oxygen transfer to a nucleophilic addend typically involves the cleavage of a relatively weak oxygen-oxygen or metal-oxygen a-bond [1077, 1207]. Chiral oxaziridines provide synthetically useful reagents for the asymmetric transfer of an oxygen atom to a variety of substrates such as sulfides and sulfoxides; the mechanism of oxygen transfer has been discussed [1208].

Electron transfer in biological systems (e.g., proteins) and electron relays that are bound to an enzyme (to give enzyme electrodes) is a topic of current interest [1209]. This comprehensive topic also includes electron transfer between porphyrin-bound Fe^2+^ centers and histidine-bound [Ru(NH_3_)_5_]^3+^ ions [1210,1211], electrical properties of enzyme proteins modified via ferrocene-ferricinium carboxylate electron relays [1212], biosensor electrodes based on ferrocenes [1213], biosensors in medicine [1214], chemical sensors [1215], and the interfacial electrochemistry of promoter-modified electrodes for rapid electron transfer of cytochrome C [1216].

The bleomycins are glycopeptide-derived antitumor antibiotics believed to exert their therapeutic effects via DNA degradation [1121,1217]. Bleomycin-mediated DNA degradation requires O_2_ and a redox-active metal ion such as Fe, Cu, or Mn [1218,1219]. Fe-bleomycin cleaves DNA by initial abstraction of H-4′ from 2-deojty-D-ribose [1218–1220], by a putative high-valent iron-oxo species; the primary kinetic isotope-effect for the cleavage reaction is *K*_H_*/K*_D_ = 2.1 – 4.0 [1220]. Extensive mechanistic studies of DNA degradation, including examination of O_2_- vs H_2_O_2_-supported activation of Fe-bleomycin, have been addressed and discussed [1220–1222].

Other sequence-specific oxidative cleavages of DNA by designed metalloproteins have been reported [1223].

A kinetics study of how the tyrosine free-radical generated by an Fe(III)_2_ subunit can abstract a H-atom from C-3′2H of *E. coli* ribonucleotide reductase has been reported [1224]. The subunit has been found to be similar to an η-oxo-bridged Fe(III)_2_ site of hemerythrin [1225].

The non-heme iron monooxygenase system from *Pseudomonas oleovorans* was examined for possible octene epoxidation. There was no evidence for the involvement of an iron-carbene species in the mechanism of octene epoxidation by *P. oleovorans* monooxygenase [1226].

Porphyrins that contain metal-oxo bonds have been extensively studied as models for the active sites of the heme protein of the peroxidases, catalases, and cytochrome P-450. Catalases decompose hydrogen peroxide to afford oxygen and water, whereas peroxidases oxidize organic and inorganic substrates via reaction with peroxides and other oxidants. The active sites of both enzymes are porphyrin cation radicals that contain ferryl (Fe = 0) bonds. Cytochrome P-450 [556,1227], which hydroxylates a variety of organic molecules (e.g., hydroxylation of C–H bonds) via an oxygen atom transfer mechanism, reduces molecular oxygen, to generate a ferryl intermediate. Studies on this putative iron-oxo species are difficult, and efforts have been directed to synthetic iron porphyrins as models for the heme porphyrins [1228]. Resonance Raman spectroscopy has been used extensively to characterize the metal-oxo bonds in ferryl porphyrins [1229,1230]. It is now generally recognized that binding of ligands to heme proteins is regulated by multiple free-energy barriers [1231].

Other work includes the catalytic [1232] and photophysical [1233] properties of porphyrins or tetraarylporphyrins [1234] anchored to synthetic vesicles, and ^1^H-NMR studies of iron(III) porphyrins [1235].

Asymmetric synthesis has emerged as a rich and rapidly developing area of chemistry, combining elements of organic synthesis, molecular recognition, metal coordination chemistry, and catalysis. Of the various strategies for exploiting the pool of chiral compounds available, catalytic asymmetric induction offers the distinct advantage of chemical amplification of the asymmetry of the catalyst.

The discovery that iron porphyrins will catalyze alkane hydroxylation and alkene epoxidation in the presence of such oxygen donors as iodosylbenzene [1236] has provided an opportunity to use synthetic porphyrins for modeling the oxygen-transfer reaction of cytochrome P-450 [1237]. Chiral metalloporphyrins have been shown to mediate catalytic, asymmetric oxygen-transfer, to afford optically active epoxides from prochiral alkenes [1238]. Similar behavior has been reported for chiral “basket-handle” porphyrins [1239]. Regioselective epoxidation [1240] and hydroxylation [1241] have also been achieved with membrane-spanning metalloporphyrins encapsulated in synthetic vesicles. Here, the synthesis and characterization of new vaulted porphyrins with a chirotropic binaphthyl bridge has been described. The iron(III) and manganese(III) derivatives of this porphyrin have proved to be robust catalysts for alkene epoxidation, sulfoxidation, and asymmetric hydroxylation [1242].

Additional new information on ferrocene includes: synthesis and oxidation of ferrocene-capped cobalt clathochelates [1243], synthesis of a linear polymer ω-ferrocenecarboxamido-α-methoxypolyethylene oxide [1244]. The synthesis and structure of [1] ferrocenophanes containing Ti, Zr, and Hf in the bridge [1245], and the synthesis of a ferrocene with a pentaarsacyclopentadienyl ligand [1246]; alkoxymethylation of ferrocenylalkenes [1247], the influence of steric hindrance on the lithiation of ferrocenylalkylamines [1248], the dynamics of ferrocene in a thiourea inclusion matrix [1249], and the coadsorption of ferrocene alkanethiols on gold films [1250] have also been studied.

#### 9.11.8 Additional Results

Metal-catalyzed oxidation of organic compounds is an expanding area of organic chemistry, with many applications in industrial processes [1251,1252]. Macrocylic metal complexes, in particular metalloporphyrins, have attracted attention as mild aerobic catalysts in oxidation reactions [1253].

The synthesis and characterization of the “picnic basket” porphyrins, which have a rigid cavity of variable dimensions on one face of the porphyrin ring have been described [1254]. The picnic basket system was designed to effect catalytic, shape-selective oxygenations [1232,1255] and thus to mimic the enzyme family cytochrome P-450.

Of the various oxidation reactions catalyzed by cytochrome P-450 enzymes, benzylic hydroxylation is a particularly favorable process. Important examples include toluene [1256], the antioxidant BHT [1257], the analgesic alkaloid morphine [1258], and the carcinogen dimethylbenz[a]anthracene [1259]. For these otherwise diverse substrates, it might logically be assumed that benzylic hydroxylation [1204] is favored over other possible reactions because of resonance stabilization of benzylic intermediates and the transition states leading to their formation [1260].

The bleomycins constitute a family of antitumor antibiotics that are considered to elicit their chemotherapeutic effects via degradation of chromosomal DNA [1217]. Studies carried out in cell-free systems using isolated DNAs have indicated that DNA degradation involves metallobleomycins that are activated in the presence of dioxygen and subsequently bind to and degrade substrate DNAs [1261]. Bleomycin-mediated DNA degradation is sequence-selective, as a study on the interaction of bleomycin with a methylated DNA oligonucleotide indicated. The extent of DNA cleavage by Fe-bleomycin A_2_ can be diminished substantially in proximity to (5)-methylated cytidine residues [1262].

Iron tyrosinate proteins are now recognized as a distinct class of non-heme proteins, that includes transferrins, the purple acid phosphatases, and a number of aromatic ring-cleaving dioxygenases [1263,1264]. These proteins all contain high-spin Fe(III) characterized by a *g* = 4.3 EPR signal and a strong absorption band in the 400–600 nm range (*e*_m_ = 20000–40000 m^−1^cm^−1^) due to a phenolate oxygen (a tyrosine ligand) to iron(III) charge-transfer (CT) transition [1265].

The enzyme lipoxygenase plays an important role in polyunsaturated fatty acid metabolism in plants and animals. In circulating blood cells, lipoxygenase inaugurates the biosynthesis of the leukotrienes, a family of mediators of medically relevant biological activities [1266]. The enzyme has received considerable research attention from chemists in recent years. Lipoxygenase catalyzes a stereospecific autoxidation reaction and has been used in the preparation of synthetically useful chiral moieties [1267]. The enzyme also contains an unusual non-heme iron atom in an as yet unidentified structural environment [1268]. The role of the iron atom in catalysis has become the focus of studies of the mechanism of action of lipoxygenase [1269, 1270].

A new iron(III) catalyzed degradation of aliphatic aldehydes to their lower homologues, with implications for lipid peroxidation chemistry, has been described [1271].

Efforts have been directed towards developing oxide-bridged Fe carboxylate chemistry, particularly high nuclearity [799] Fe carboxylates Fe_6_, Fe_8_, Fe_16_, and Fe_16_ M(M = CO,Mn) [1272,1273].

The mutual interaction of aromatic rings of aromatic amino acid residues in proteins and peptides has been discussed [1274]. Incorporation into peptides and proteins of amino acids having a covalent link between their aromatic rings would give the stabilization of the tertiary structure of a protein, with interesting biological consequences. The 1,1′-disubstituted ferrocenyl amino acid [1,1′-ferrocenylbis(alanine)] may be considered to be an analog of two aromatic amino acids having conformationally locked side-chains, and its synthesis (to give optically active material) has been described [1275].

Chiral recognition to the extent of 30:1 has been observed in the reaction between the homochiral iron acyl complex [(η^5^-C_5_H_5_)Fe(CO) (PPh_3_)COCH_2_OCH_2_Ph] and racemic 1-phenylethyl bromide [1276].

Reagents that react specifically with protein chains and deoxyribonucleic acid (DNA) are extremely useful in chemistry and biology. A few biological cleavage reagents are based on iron-EDTA complexes. The substitution and redox reactions of ethylenediaminetetraacetic acid (EDTA) and related polyamino carboxylate complexes of Fe(II) and Fe(III) have increasingly received attention for the sequence-specific cleavage of DNA [1277–1279]. In addition, a number of mechanistic studies have been reported on the $\text{Fe}\left(\text{II}/\text{III}\right)/H_{2}O_{2}/{\text{HO}}_{2}^{-}$ system and for related polyamino carboxylate complexes [1280,1281].

The ability to bind and sever double helical DNA at a particular sequence of bases in the presence of a vast number of other sequences of bases is one of the central challenges in molecular biology. Restriction enzymes carry out such a reaction, but most restriction enzymes, useful as they are, recognize only four to six DNA base pairs, although a few recognize eight base pairs. Progress has been made in recognition by the pyrimidine oligonucleotide of thymine-adenine base pairs in duplex DNA [1282] and in designing of oligonuceotides capable of binding alternate strands of DNA [1283]. Coupled with this study, research on yeast chromosome advanced toward a practical method of producing sequence-specific, DNA-cleavage reagents targeted toward a large number of potential binding-sites. It has been shown [1284] that a synthesized 20-base pyrimidine oligonucleotide (EDTA-Fe)_2_ reagent cleaves the yeast chromosome into two fragments that are about 230,000 and 110,000 base pairs in length. Thus, the reagent binds to, and severs, the chromosome of the almost 14 million base-pairs of DNA that make up the yeast genome.

The ability of chemical reagents selectively to cleave peptides and proteins at defined sequences can greatly facilitate studies of protein structure and function. Protein-cleavage reagents permit sequence analysis of large or blocked proteins, functional analysis of protein domains, and structural analysis of receptors; the could also lead to the development of new therapeutic agents. A new EDTA reagent has been found effective in cleavage of calmodulin, a calcium receptor protein that plays an important role in cellular regulation. The reagent consists of the iron chelator EDTA covalently bound to the calmodulin antagonist trifluoroperazine -EDTA (TFE). In the presence of Fe(II) and oxygen, TFE oxidatively cleaves the protein to produce six major cleavage fragments. The cleavage reagent only cleaves calmodulin that has bound four calcium ions [1182].

A similar cleavage reagent targets the protein streptavidin, a protein that binds biotin. Here, EDTA is attached to biotin, which then delivers redox-active copper or iron to the binding sites of the protein. In the presence of oxygen and Fe(III) or Cu(II), the biotin-EDTA reagent (in which the two groups are closely linked) cleaves streptavidin in a manner that suggests that the cleavage reaction occurs close to the biotin site of the protein. Biotin completely inhibits the cleavage reaction [1183].

Purple acid phosphotases are important iron-containing, non-heme proteins that catalyze the hydrolysis of activated phosphoric esters [1285,1286]. A new synthesis of Zn(II)Fe(III) and Fe(II)Fe(III) complexes containing an (η-phenoxo)bis(η-diphenylphosphato)-dimetal(II,III) core as a model complex for an active site of purple phosphatase has been reported [1287].

The role of conformational changes of cytochrome C that occur during electron transfer with its in vivo reaction partners has attracted some attention [1288]. Here, the rate of conformational changes of cytochrome C during electron transfer, determined by double potential step chronoelliptometry, has been reported [1289].

Chiral ferrocene derivatives are highly useful ligands for homogeneous asymmetric catalysis [1290] and peptide synthesis [1291]. They have been prepared by using classical resolution techniques [1292] and subsequently by enzymatic kinetic resolution [1293]. An efficient method for the synthesis of (*R*)-1-ferrocenylethylamine and 1-ferrocenylethyl acetate via highly diastereoselective hydride reduction of the corresponding imine has been reported [1294].

There is broad agreement that the carbon dioxide content of the atmosphere and the earth’s climate are closely linked. Carbon dioxide is the most important of the “greenhouse” gases, which also include methane and chlorofluorocarbons. An intriguing scientific hypothesis is that the iron content of some large expanses of ocean may actually determine the biological productivity of those waters. Consequently, “fertilizing” the ocean with iron might be able to control, in part, the level of carbon dioxide in the atmosphere [1295].

One of the proposed models [1296] incorporates the notion of a “biological pump,” that is, photosynthetic uptake of CO_2_ by the chlorophyll-containing marine microorganisms known as phytoplankton, and subsequent removal of carbon to the deep ocean when the remains of the phytoplankton sink away from the surface. When the pump is functioning efficiently, atmospheric CO_2_ levels are low, and vice versa.

Ferritin is a protein that is widespread in nature, including bacteria, plants, and animals, and its function is usually associated with iron storage, e.g., biomineralization of iron [1199,1297].

Although the presence of metalloporphyrins in fossil fuels has been recognized for more than fifty years, it is only recently that iron porphyrins have been satisfactorily identified in coals and lignites [1298]. In recent years, there has been extensive study of the paramagnetically shifted H-NMR spectra of iron porphyrins, especially in relation to hemoprotein structure [1299,1300].

#### 9.11.9 Pertinent Books and Reviews

A book on the chemistry and biochemistry of *N*-substituted porphyrins has been published [1301]; it reviews, and critically evaluates, the field of *N*-substituted porphyrins and their metal complexes; among the topics discussed are spectroscopic properties, and inhibition of ferrochelatase and cytochrome P-450.

A book on the spectroscopy of iron porphyrins and heme proteins has been published [1302].

Another book, on organic transformations, has appeared [1303]; the volume (1160 pages) covers a wide area of interest to the organometallic chemist, including, for example, new synthetic methods, enantioselective catalysts, and metal-promoted coupling reactions.

A useful addition to the library of the organometallic chemist is a book on reactions of coordinated ligands [1304]; it discusses reactions of coordinated CO_2_ and *N*,*N*-dialkylcarbamates, hydrolysis and condensation reactions of *O*- and *N*-bonded ligands, reactions of coordinated phosphorus and sulfur ligands, and other topics.

New aspects of organic chemistry have been discussed in a monograph [1305].

Pertinent reviews include metallocenes as reaction intermediates [1306], main-group metallocenes (recent developments) [1307], porphyrin-quinone compounds as synthetic models of the reaction center in photosynthesis [1308], enzymatic catalysts in organic synthesis [1309], from natural cleomycins to man-designed cleomycins [1310], molecular recognition and metal ion template synthesis [1311], transition-metal templates as guides for cycloaddition [1312], a new approach for natural-product synthesis using main-group organometallic reagents [1313], advances in catalytic asymmetric reactions promoted by transition-metal complexes [1314], stereoselectivity of intermolecular, free-radical reactions [1315], phosphaalkynes and phosphaalkenes [1316], transition-metal complexes of unsaturated carbenes [1317], heterometallic, sulfide-bridged dusters of transition elements [1318], and highly reactive intermediates from condensation reactions of iron, cobalt, and nickel vapors with arenes [1319].

#### 9.11.10 Other Miscellaneous Results

Additional new work included addition of halogencarbons to alkenes in the presence of [Fe_2_(CO)_4_(η-C_5_H_5_)_2_] [1320], use of [Fe(CO)_3_NO]^−^ for the carbonylation of primary, secondary, and allylic halides [1321], a new synthesis of azaferrocene, (η^5^-C_4_H_4_N)(η^5^-C_5_H_5_)Fe [1322], synthesis of Fe_2_(CO)_6_(η-CRCR^1^COEtH) complexes (R = R^1^ = Ph; R^1^ = Ph,Ri^1^ = Me) [1323], 1,3- dipolar cycloaddition to the C=N–Fe fragment [1324], rearrangement of η^5^-C_5_H_5_(CO)_2_Fe-η^1^-homoallylidene to η^5^-C_5_H_5_(CO)_2_Fe-η^1^-allylidene [1325], reaction of allene episulfide with Fe_2_(CO)_9_ [1326], reaction of [bis(dimethylgermyl)alkane] iron tetracarbonyls with carbonyl compounds [1327], and synthesis of a new organic conductor, bis(ethylenedithio)tetratiafulvalene-iron oxychloride [1328].

Other studies reported on the photochemical insertion of alkenes into the S–S bond of cluster [(CO)_3_FeS]_2_ [1329], insertion of a methylene group into the Te–Te bond of Fe_2_(CO)_6_(η^2^-Te_2_) [1330], the molecular structure of the binuclear complex Fe_2_(CO)_6_(η-PhNC(O)C_6_H_4_NH) synthesized from Fe(CO)_6_ and azobenzene [1331], formation and properties of a NiFe_3_S_4_ cluster in *Pyrococcus furiosus* ferredoxin [1332], x-ray structure of cluster Au_2_Fe_2_Ir(η^4^-C_2_Ph)(CO)_7_(PPh_3_)_3_ [1333], synthesis of a “cascade-type” quest organotin(IV) coordination polymer [(Me_3_Sn)_4_Fe(CN)_6_·2H_2_O·C_4_H_8_O_2_] ∞ [1334], reaction of carbido cluster Fe_5_C(CO)_15_ with metallic As and Bi [1335], and x-ray diffraction study of a pyramidal cluster [Fe_5_C(CO)_12_(PMe_3_Ph)_3_ [1336,1337], the crystal structure of the mixed-valence 1′,l‴-dibenzylbiferrocenium cation [1338], substitution reactions of the solvent-coordinated acyl complexes, e.g., (η^5^-C_5_H_5_)(CH_3_COCH_3_)FeCOMe^0,+^ involving thioethers [1339], and MO theory into the problem of the Fe–Fe bond in Fe_2_(CO)_9_ [1340]. Other selected topics include kinetic and photochemical studies of ${\text{FeC}}_{5}H_{6}^{+}$ in the gas phase [1341], gas-phase reactions of Fe^+^ with aromatic compounds [1342], binuclear iron(III) complexes with squarate as a bridging ligand [1343], synthesis of macrocyclic tetraamide five-coordinate iron(IV) complex [1344], of boron-containing macrocyclic iron(II) complexes [1345], of octahedral hydrido stannyl complexes of iron [1346], and of a Fe_4_S_4_ cubane-type iron-sulfur protein analog [1347]. Other topics covered are: acetolysis of ferrocenylmethyl benzoate [1348], mixed arene ferrocenes [1349,1349a], pyrrolyl iron complexes (octamethyl-1,1′-diazaferrocene) [1350], monoacetylferrocene thiosemicarbazone complexes [1351], ferrocene-containing cryptands [1352], picket fence porphyrins [1353], chiral “single-armed” porphyrins [1354], crystal structure of BiFe(CN)_6_-4H_2_O [1355], crystal structure of *N*-methyl-substituted η-OXO diiron(III) tetraphenylporphyrin [1356], x-ray structure of iron dimer [(CH_2_COCH_2_)(CO)_3_Fe]_2_ [1357], crystal structure of [Fe_2_(CO)_6_(η-PPh_2_(ηCPh = CPhH) [1358], synthesis of a tripledecker 2π-ligand RPFe_2_(CO)_6_ [from cluster Fe_3_(CO)_10_(η^3^-RP)] [1359], reaction of *O*-fluorolithiobenzene with dicarbonyl (η^5^-cyclopentadienyl) iodoiron [1360], photo-induced redox reaction of $\text{Fe}\left(2,2^{\prime }-\text{bipyridine}\right){\left(\text{CN}\right)}_{4}^{2-}$ [1361], and high-affinity iron-chelating agents for clinical use [1362]. Other pertinent articles treat the study of the iron-phosphorus bond (e.g., ligand effects) [1363], four-legged piano-stool structures (a theoretical study) [1364], structural study of bis(thiocyanato)bis(2,2′-bi-2-thiazoline) iron(II) complexes [1365], ET reactions of Fe(II) and Fe(III) bis(oxime-imine) complexes [1366], reaction of iron-alkene ions with chlorobenzene in the gas phase [1367], magnetic characterization of [Fe(C_5_Me_5_)_2_]^·+^[TCNQI_2_]^·−^ (electron-transfer complexes) [1368], and MO analysis on the dimer (CO)_3_Fe(η-CO)_3_Fe(CO)_3_ and related tetranuclear clusters [1369]. Other studies included that of conversion of 2,5-dihydrothiophene 1,1-dioxide into highly functionalized (η^4^-buta-1,3- diene) tricarbonyliron (0) complexes [1370], an efficient method for studying biosynthetic oxidation by using cytochrome P-450 inhibitors [1371], oxidation of aldehydes by an iron(III) porphyrin complex-*m*-chloroperbenzoic acid system [1372], formation of iron acetylides and diacetylides *via* complexes of molecular hydrogen [1373], an electron-transfer-chain-catalyzed chelation of the dithiocarbonate ligand in Fe(η^5^-C_5_Me_5_)(CO)_2_ [1374], metal complexes of macrocyclic polyamines [1375], structural studies of Fe_4_S_4_-siroheme and related heme proteins [1376], reactions of dimethylamine with multiply charged ions of cytochrome C [1377], observation of the Fe(II)-O_2_ stretching Raman band for cytochrome oxidase compound A [1378], and some new chiral, analytical techniques [1379].

## Figures and Tables

**Table 1:** Cyclopropanation of alkenes [209] with $\left[\left(\eta^{5}{\text{-C}}_{5}H_{5}\right){\left(\text{CO}\right)}_{2}{\text{FeCH}}_{2}S^{+}{\left({\text{CH}}_{3}\right)}_{2}\right]{\text{BF}}_{4}^{-}\left(53\right)$^a^

Alkene	Cyclopropane	Conversion of Alkene (%)	Yield of Cyclopropane (%)
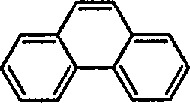	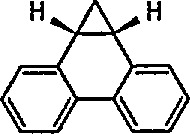	38	82
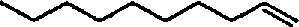	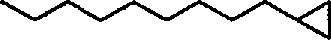	81	70
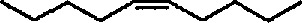	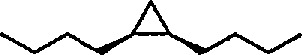	59	87
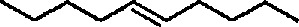	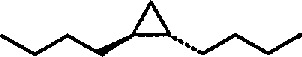	49	67
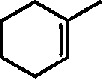	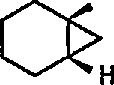	90	26
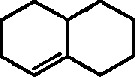	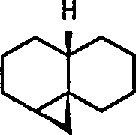	70	52
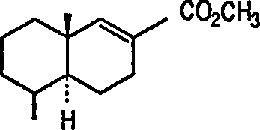	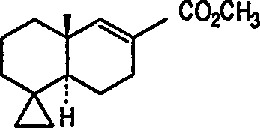	100	86
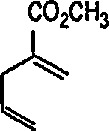	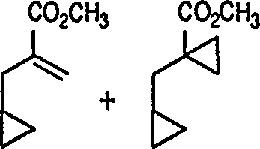	99	62 + 5
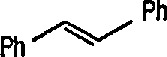	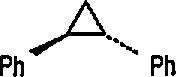	62	64
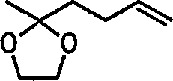	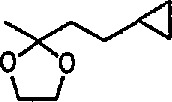	100	22

a Unless othenwise indicated, these reactions were conducted under a standard set of conditions employing 2 molar equiv. of 53 and 2M solution of alkene in trailing 1,4-dioxane at reflux for 12 h.

**Table 2:** Synthesis of β-lactams from β-amino iron acyls [329]

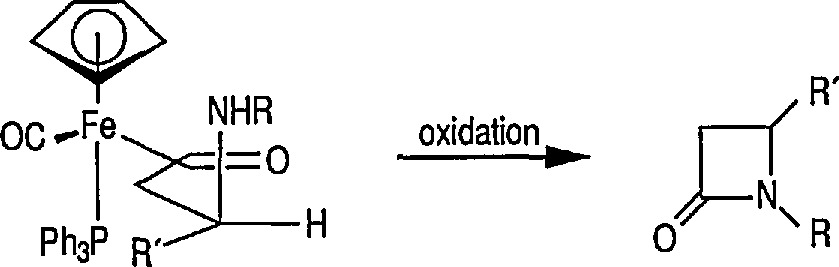			

R′	R	Reaction Conditions	β - lactam yield, %
Ph	Ph	I_2_/CH_2_Cl_2_/PhNMe_2_/−42 °C	80
Ph	η - Pr	I_2_/CH_2_Cl_2_/PhNMe_2_/room temp	70
Ph	η - Pr	Br_2_/CS_2_/−42 °C	82
Ph	CH_2_Ph	Br_2_/CS_2_/−78 °C	79
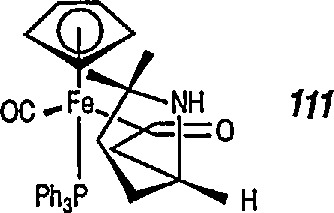			
		Br_2_/CS_2_/−45 °C	56
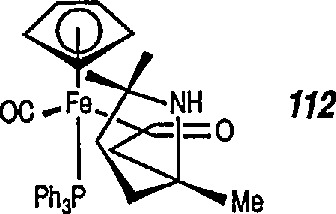			
		I_2_/CH_2_Cl_2_/Et_3_N	62